# Unprecedented organelle genomic variations in morning glories reveal independent evolutionary scenarios of parasitic plants and the diversification of plant mitochondrial complexes

**DOI:** 10.1186/s12915-022-01250-1

**Published:** 2022-02-16

**Authors:** Yanxiang Lin, Pan Li, Yuchan Zhang, Delara Akhter, Ronghui Pan, Zhixi Fu, Mingqing Huang, Xiaobo Li, Yanlei Feng

**Affiliations:** 1 College of Pharmacy, Fujian University of Traditional Chinese Medicine, Fuzhou, 350122 Fujian China; 2 MOE Laboratory of Biosystem Homeostasis and Protection, College of Life Sciences, Zhejiang University, Hangzhou, 310058 Zhejiang China; 3 Institute of Crop Science, College of Agriculture and Biotechnology, Zhejiang University, Hangzhou, 310058 Zhejiang China; 4 Department of Genetics and Plant Breeding, Sylhet Agricultural University, Sylhet Division 3100, Sylhet, Bangladesh; 5 ZJU-Hangzhou Global Scientific and Technological Innovation Center, Zhejiang University, Hangzhou, 310027 China; 6 College of Life Science, Sichuan Normal University, Chengdu, 610101 Sichuan China; 7 Key Laboratory of Growth Regulation and Translational Research of Zhejiang Province, School of Life Sciences, Westlake University, Hangzhou, 310024 Zhejiang China; 8 Institute of Biology, Westlake Institute for Advanced Study, Hangzhou, 310024 Zhejiang China

**Keywords:** Convolvulaceae, *Cuscuta*, Plastid genome, Mitochondrial genome, *ccmFc*, Horizontal gene transfer

## Abstract

**Background:**

The morning glories (Convolvulaceae) are distributed worldwide and produce economically important crops, medicinal herbs, and ornamentals. Members of this family are diverse in morphological characteristics and trophic modes, including the leafless parasitic *Cuscuta* (dodders). Organelle genomes were generally used for studying plant phylogeny and genomic variations. Notably, plastomes in parasitic plants always show non-canonical features, such as reduced size and accelerated rates. However, few organelle genomes of this group have been sequenced, hindering our understanding of their evolution, and dodder mitogenome in particular.

**Results:**

We assembled 22 new mitogenomes and 12 new plastomes in Convolvulaceae. Alongside previously known ones, we totally analyzed organelle genomes of 23 species in the family. Our sampling includes 16 leafy autotrophic species and 7 leafless parasitic dodders, covering 8 of the 12 tribes. Both the plastid and mitochondrial genomes of these plants have encountered variations that were rarely observed in other angiosperms. All of the plastomes possessed atypical IR boundaries. Besides the gene and IR losses in dodders, some leafy species also showed gene and intron losses, duplications, structural variations, and insertions of foreign DNAs. The phylogeny reconstructed by plastid protein coding sequences confirmed the previous relationship of the tribes. However, the monophyly of ‘Merremieae’ and the sister group of *Cuscuta* remained uncertain. The mitogenome was significantly inflated in *Cuscuta japonica*, which has exceeded over 800 kb and integrated massive DNAs from other species. In other dodders, mitogenomes were maintained in small size, revealing divergent evolutionary strategies. Mutations unique to plants were detected in the mitochondrial gene *ccmFc*, which has broken into three fragments through gene fission and splicing shift. The unusual changes likely initially happened to the common ancestor of the family and were caused by a foreign insertion from rosids followed by double-strand breaks and imprecise DNA repairs. The coding regions of *ccmFc* expanded at both sides after the fission, which may have altered the protein structure.

**Conclusions:**

Our family-scale analyses uncovered unusual scenarios for both organelle genomes in Convolvulaceae, especially in parasitic plants. The data provided valuable genetic resources for studying the evolution of Convolvulaceae and plant parasitism.

**Supplementary Information:**

The online version contains supplementary material available at 10.1186/s12915-022-01250-1.

## Background

Convolvulaceae Juss., also known as the morning glories and bindweeds, is a large family belonging to the order Solanales of the eudicots. It contains approximately 1900 species from 59 genera [1]. Members of the family include crops (e.g*.*, sweet potato), vegetables (e.g., water spinach), medicinal plants (e.g., dried seeds of dodders and vines of *Erycibe*), ornamentals (e.g., morning glory, cypress vine, and moonflower), and tough weeds (e.g., dodders). Convolvulaceae is cosmopolitan and exhibits a rich diversity of morphological characteristics comprising herbs, shrubs, trees, climbers, and leafless parasitic plants *Cuscuta* (dodders).

In the past decades, species in Convolvulaceae were classified into 12 tribes and six clades using several DNA markers [2–5]. With great advances in genome sequencing technologies, plastid genomes (plastomes) of many plant taxa were completed. However, the plastome studies of Convolvulaceae mainly focused on *Ipomoea* [6–8] and dodders [9–12]. Many genera of this family were neglected, leaving the phylogenetic relationship untested with larger datasets and the positions of the ‘Merremieae’ and Cuscuteae unresolved [4, 13]. Additionally, only two mitochondrial genomes (mitogenomes) in this family were known, one is recently published *Evolvulus alsinoides* [14] and the other is *Ipomoea nil*, which was obtained from the whole genome sequencing project [15].

On the other side, organelle genomes in Convolvulaceae exhibited multiple peculiar characteristics. For example, the intron deletion of *rpl2* in Convolvulaceae plastomes was a synapomorphy and a unique event in Asteridae [3]. The *infA* has been lost from the plastome, and inverted repeat (IR) boundaries have been altered in *Ipomoea* and dodders [8, 12]. The mitogenome of *I. nil* is only 266 kb in length, which is much smaller than most known mitogenomes in angiosperms. Moreover, annotation of the mitogenome (GenBank ID: AP017303) reveals a very unusual *ccmFc* gene, with fragments located in three different positions. Further studies are required to reconcile these findings. Plastomes of dodders, like many other heterotrophic plants (such as [16, 17]), exhibited smaller size, reduced genes, variable structure, and elevated nucleotide substitutions [10, 11, 18]. The nuclear genomes of dodder also experienced massive gene losses [19, 20]. In contrast, the evolutionary pattern of their mitogenomes remains unexplored.

To unlock the secrets of organelle genomes of Convolvulaceae, we assembled 22 mitogenomes and 16 plastomes. Our collections covered five of the six clades and eight of 12 tribes, representing one of the most comprehensive samplings of this group. We unveiled the unusual organelle genome variations and depicted their evolutionary trajectories. Mitogenomes in parasitic dodders have evolved towards different directions, either acquiring large amounts of foreign DNA or retaining a small size. Unusual mutations were detected in the mitochondrial *ccmFc* in all family members, and omics data were employed to confirm the gene fission, splicing shift, and coding expansion.

## Results and discussion

### Plastome variations in Convolvulaceae

**Fig. 1:**
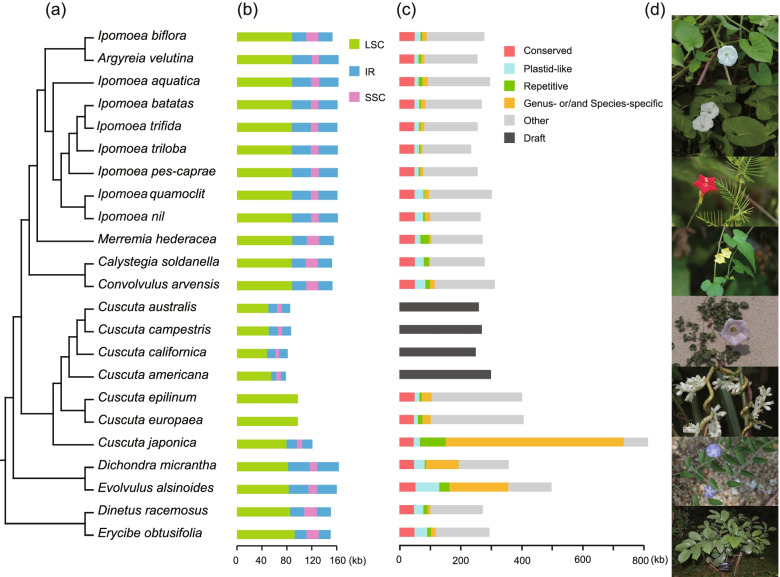
Sampling, plastome and mitogenome content, and phenotypic diversity of Convolvulaceae. **a** Samplings of this study. Their relationship was referred to the plastid CDS tree in Fig. 5. **b** Plastome size and structure. LSC long single-copy region, IR inverted repeat region, SSC short single-copy region. **c** Mitogenome size and proportions. The total lengths of bars represented the length of each mitogenome, with different colors indicating the proportion of each sequence type. The four species from *Cuscuta* subgenus *Grammica* only had draft sizes determined without the proportions quantified. **d** Morphology of *I. biflora*, *I. aquatica*, *I. quamoclit*, *Merremia hederacea*, *Calystegia soldanella*, *Cu. japonica*, *Evolvulus alsinoides*, and *Erycibe obtusifolia* (from up to bottom)

**Fig. 2:**
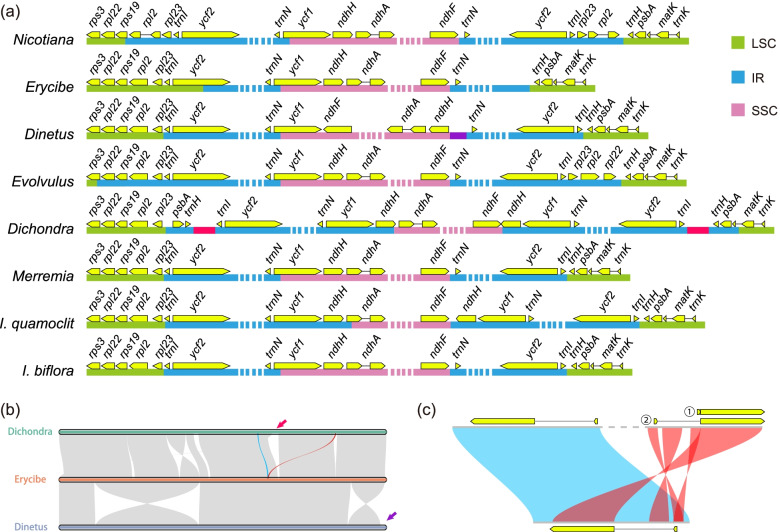
Plastome variations in Convolvulaceae. **a** IR boundary changes. *Calystegia* and *Convolvulus* and other *Ipomoea* spp. were not shown since they are similar to *I. quamoclit* in structure. The purple and pink segments located in *Dinetus* and *Dichondra* indicate foreign insertions. **b** Plastomes (last IR removed) of *Dichondra* and *Dinetus* have experienced inversions. The pink and purple arrows showed the position of the foreign insertions, which had no homologs with other plastomes. The red ribbon exhibited the position of (**c**). **c** Dispersed repeats in *Dichondra* may create another copy of *rpl16*. The first exon was duplicated twice. Therefore, the second copy of *rpl16* may have two potential combinations (labeled with numbers 1 and 2)

Our assemblies include at least 12 new plastomes, among which six genera were sequenced for the first time (Additional file 1). We also assembled four dodders for the first time, two from the subgenus *Cuscuta* (*Cu. epilinum* and *Cu. europaea*), and two from the subgenus *Grammica* (*Cu. americana* and *Cu. californica*). These new plastomes showed similar features to the known ones in *Cuscuta*, including the losses of genes, introns, and IR regions (Fig. 1b; Additional file 1) [12, 21]. In the leafy species, the plastid DNA sequences or the gene content did not differ much, and the main distinction came from the variation of the IR regions (Figs. 1b and 2a; Additional file 1). IR regions expanded or shrank in different directions and resulted in different architectures—all the species in this family were atypical in IR boundaries (Fig. 2a). *Dinetus* and *Dichondra* contained some inversions, and they were the only two leafy species that lost the gene *rpl23* (Fig. 2b; Additional file 1). The *Dichondra* plastome also had some unusual duplications, which could create another copy of *rpl16* (Fig. 2b, c, blue and red ribbon represented the original and second copy of *rpl16*, respectively). *Rps16* and *rpoC1* in *Evolvulus*, and *rps16* and *ycf3* in *Dichondra* have experienced intron losses. *InfA* was lost in Convolvulaceae, and BLASTP searches in Convolvulaceae nuclear peptides have confirmed the transfer to nucleus, like many other species [22].

*Dinetus* and *Dichondra* had a *ca.* 1.5 and 2.2 kb foreign DNA inserted into the short single-copy (SSC) region and the IR region, respectively (Fig. 2a, b, purple and red bars and arrows). These two foreign sequences had no homologs in Convolvulaceae mitogenomes, nor were they similar to any sequences in NCBI *nt* database (no significant hits were yielded from BLASTN searches), so their origins were mysterious. Foreign DNAs in plastomes were mainly known to be transferred from mitogenome, such as in Apiaceae [23–25], Apocynaceae [26], Anacardiaceae [27], Orobanchaceae [28], and Poaceae [29–32], while transfers from other sources were rare [25]. The foreign insertions in *Dinetus* and *Dichondra* plastomes could represent two additional examples of non-mitochondrial origin.

### Mitogenome variations in Convolvulaceae

We assembled mitogenomes of 22 species in the family Convolvulaceae, making this one of the few studies comprising a large number of new and complete mitogenomes in plants (Fig. 1c, Additional file 2). Complete mitogenomes were obtained for 18 of these, including all leafy species, *Cuscuta epilinum*, *Cu. europaea*, and *Cu. japonica*, while only drafts were obtained for the four dodders from subgenus *Grammica* (*Cu. americana*, *Cu. australis*, *Cu. californica*, and *Cu. campestris*) due to large amounts of repeats. Mitogenomes in Convolvulaceae are mostly around 300 kb in length, which is shorter than most angiosperms, including their close relatives Solanales. The gene content of Convolvulaceae mitogenomes is similar to other angiosperms (Additional file 2). The “core” genes are well preserved except *ccmFc* (see below). Compared to Solanales, *rpl2* and *sdh3* were missing in all family members investigated. The *rps7* was only found in *I. biflora*, and phylogenetic analysis indicated that it was closest to Rosales (bootstrap value 99% with *Morus notabilis* and *Cannabis sativa*; Additional file 3, *rps7*). Therefore, the *rps7* most likely have been lost in the common ancestor of Convolvulaceae and *I. biflora* re-gained it by horizontal gene/DNA transfer (HGT). Mitogenomes of the *Grammica* were also the most degenerated in the family—they lost more genes, similar to their plastomes. *Cu. epilinum*, *Cu. europaea*, and *Cu. japonica* showed no significant differences in gene content (Additional files 1 and 2). Most mitochondrial genes in dodders had increased substitution rates, but none was from HGT (Additional file 3).

**Fig. 3:**
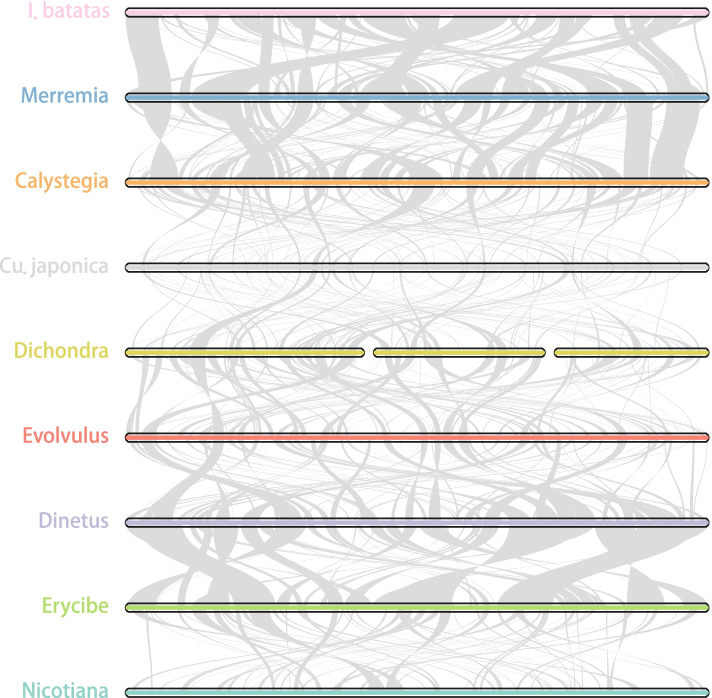
Pairwise mitogenome synteny among tribes. The bars indicated the mitogenomes, and the ribbons showed the homologous sequences between the adjacent species. *Dichondra* has three chromosomes. Only one species of each tribe was used, and their order referred to the plastid CDS tree in Fig. 5. Tobacco (*Nicotiana tabacum*, GenBank: NC_006581) was also compared

In contrast to the generally small size in the family, the mitogenome in *Cu. japonica* has exceeded 800 kb, reaching twice as large as others (Fig. 1c). Besides, *Dichondra*, *Evolvulus*, *Cu. epilinum*, and *Cu. europaea* also showed expansions. Pair-wise mitogenomic synteny of the tribes was compared, which revealed rapid changes in mitogenomic structure and DNA content (Fig. 3). To understand the causes of the difference, we dissected the mitogenomic sequences into five classes: conserved (have homologs in all Convolvulaceae), plastid-like (potential plastid insertions), repetitive, genus-, or/and species-specific and other (the rest) (Fig. 1c). Only a small proportion of DNA exists in all (mostly intragenic regions; Fig. 1c, the “Conserved” proportion). The most significant difference was from genus- or/and species-specific sequences (GSS, details see “Methods” section), especially in *Cu. japonica*.

**Fig. 4:**
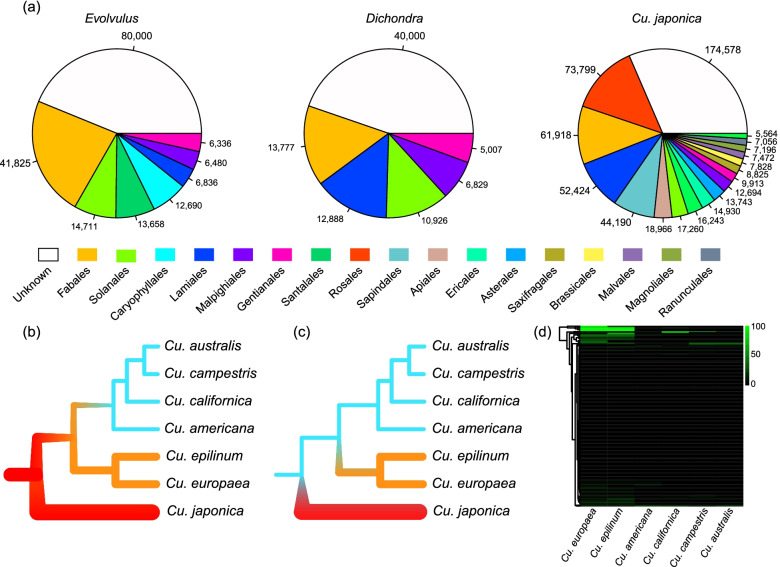
Mitogenome variations in Convolvulaceae. **a** Orders of GSS best hits in *Evolvulus*, *Dichondra*, and *Cu. japonica*. The numbers showed the total length (bp) of each order. Only orders >5 kb are displayed. The white sectors showed the GSS had no homologs in NCBI *nt* database. **b**, **c** The two potential evolutionary paths of the small mitogenome size in subgenus *Grammica*—either degenerated after inflation or stayed small. Red, yellow, and blue branches indicated the large, middle, and small mitogenome sizes, respectively. **d** Significant best hits of *Cu. japonica* GSS (>500 bp, i.e., HGT-like) were used to search for homologs in other dodders, with the coverage shown in the heatmap. Only a few could be found with homologs (green cells). This supported the possibility that the large mitogenome size of *Cu. japonica* has evolved independently from other dodders

To identify the potential origin, GSS of *Dichondra*, *Evolvulus*, and *Cu. japonica* were blasted against *nt* database and screened the best hits (Additional file 4). The best hits were further grouped into orders. Although many of them had unknown origins (i.e., had no results from the BLAST searches), most GSS shared high similarity with distantly related taxa (Fig. 4a). Fabales, Solanales, Lamiales, Malpighiales, and Gentianales occupied large proportions in the GSS in all the three species, which may indicate that their expansion has experienced similar events. In contrast, Caryophyllales and Santalales in *Evolvulus* and Rosales, Sapindales, Apiales, etc., in *Cu. japonica* could imply independent evolution.

### Divergent evolution of the mitogenomes in dodders

The parasitic plants have evolved at least 12 times independently in angiosperms [33, 34], which provides an excellent model to study plant interactions and molecular evolution. Their genomes often show characteristics not commonly observed in other plants, including gene losses and HGTs [19, 20, 35–37]. Plastomes in green autotrophic plants are very conserved, while those in parasitic plants always show reduced size, loss of photosynthesis genes, different IR boundaries, and ascending substitution rates [38], as reported for *Cuscuta* [10, 11, 18]. For mitogenomes, many obligate parasitic plants exhibited extensive HGTs from hosts, even including the replacements of the native genes, such as in *Cynomorium* (Cynomoriaceae [39, 40];), *Lophophytum mirabile* (Balanophoraceae [41, 42];, *Viscum album* (Santalaceae [43];), and *Aeginetia indica* (Orobanchaceae [44];). Therefore, it might not be surprising that *Cu. japonica* received large amounts of DNA from other species. However, mitogenome size in other dodders still resembled that of leafy species. An interesting question is how large the common ancestor of dodders was in mitogenome size—if the mitogenome of *Grammica* dodders degraded or *Cu. japonica* expanded (Fig. 4b, c). We hypothesized that if the mitogenomes shrank from big to small, there should contain some remnants, even though the remnants were very short. We used the significant HGT-like sequences of *Cu. japonica* (Additional file 4, length >500 bp) as queries to search for homologs in the other six dodders and then calculated the coverage rate of each GSS. Only a few GSS yielded hits from the other dodders (Fig. 4d). It suggested that though dodders had some HGT-like sequences in common, most expansions in *Cu. japonica* occurred independently after the speciation. The mitogenome size evolved in a divergent manner in dodders. A similar situation was also observed for *Viscum scurruloideum*, which possesses the smallest mitogenome in angiosperms (only 66 kb) while its relative *V. album* outnumbers it 8.6-fold in size [45, 46]. Why some dodders and *V*. *scurruloideum* evolved in an opposite direction, and how they maintained a small mitogenome are intriguing questions to be answered in the future.

### Phylogenetic relationships

**Fig. 5:**
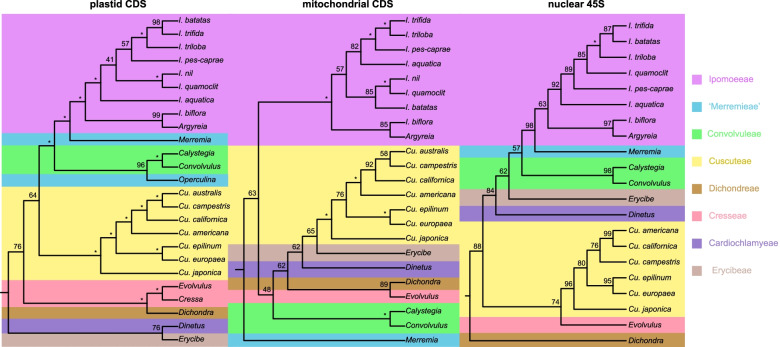
ML trees of Convolvulaceae based on plastid, mitochondrial, and nuclear sequences. Each color represented one tribe. Numbers at nodes indicated bootstrap support (as percentages of 1000 replications)

Sequences of plastid protein coding sequences (CDS), mitochondrial CDS, and nuclear 45S (18S, 5.8S, and 25S rRNAs and the spacer regions) were employed to build the maximum-likelihood (ML) trees of Convolvulaceae (Fig. 5; Additional file 5). Plastomes of *Operculina macrocarpa* (GenBank: KF242502) and *Cressa cretica* (NC_035516) were included. The plastid matrix contained 77,711 columns with 16,689 parsimony informative sites (PIS). The plastid tree has a similar topology to previous studies [3–5]. However, the two species from “Merremieae,” *Merremia* and *Operculina*, were nested with Convolvuleae. Merremieae was suggested as polyphyletic [5, 13] and then re-classified based on several DNA markers and morphological data [1]. Our result revealed that the monophyly of Merremieae was likely still uncertain. The support of the Cuscuteae clade was low, the sister group of dodders remained unresolved.

The mitochondrial matrix had 30,753 columns but only with 1033 PIS. The mitochondrial tree got a very different topology and was poorly supported. The ability of mitochondrial sequences in phylogeny in family or order level was challenged [47]. The nuclear tree suggested a closer relationship between dodders and Erycibeae. However, the support of the nuclear tree was also weak, which may also be caused by a lack of PIS (5906 columns with 548 PIS).

### All family members harbor the broken ccmFc

**Fig. 6:**
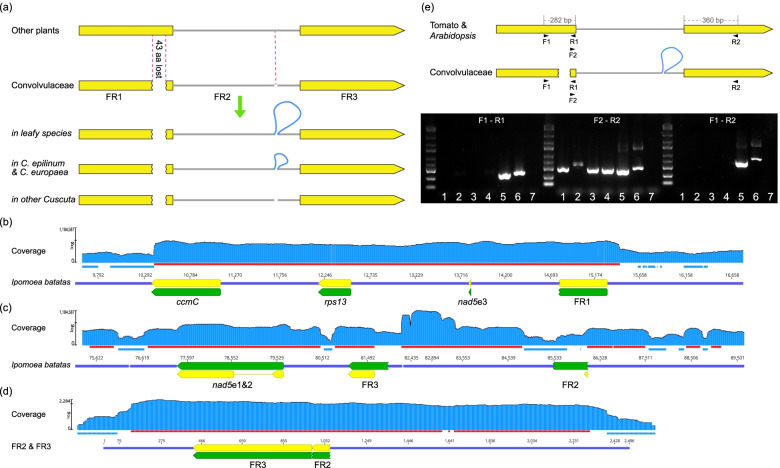
*ccmFc* encountered two independent breaks and became two genes. **a** Breakpoints in *ccmFc*. A long insertion in the second breakpoint meant that FR2 and FR3 are still adjacent in leafy species. In *Cu. epilinum* and *Cu. europaea*, the insertion is much shorter; the connection has broken in other *Cuscuta*. **b**, **c** Transcriptome coverage of the three fragments in sweet potato (*I. batatas*). Blue and red lines under the coverage indicated coverage less than 50 and greater than 400, respectively. The coverage was scaled by Log function in GENEIOUS. **d** Transcriptome coverage of the exon parts after the intergenic regions were removed. **e** Positions of primers and agarose gel analysis of RT-PCR confirming the gene fission. Gene length scales indicated on the tomato *ccmFc*. Lanes 1 to 7: *Cu. australis*, *Cu. japonica*, sweet potato, *Dichondra*, tomato, *Arabidopsis*, and no template

A very unusual phenomenon was observed in the *ccmFc* gene. *ccmFc* is one subunit of the cytochrome *c* maturation (CCM) system and is involved in the final stage of the maturation process [48, 49]. The *c*-type cytochrome is an essential component of the mitochondrial electron transport chain and delivers electrons between complexes III and IV. Typically, the gene contains two exons and a group II *cis*-splicing intron, and the structure and sequence of the gene are remarkably conserved in almost all angiosperms. Differently, in all members of Convolvulaceae, it has been divided into three fragments. The first break occurred close to the 3′-end of the first exon, causing a *ca.* 43 aa loss (between positions 164 to 206 in tomato); the second break occurred within the intron without base losses (Fig. 6a). Our data included multiple long sequencing reads, and we detected no large repeats around *ccmFc* fragments, arguing against incorrect assembly. Our rich sampling suggests the breaks most likely occurred to the common ancestor of this family.

The genomic distribution of the three *ccmFc* fragments varied among species (Fig. 6a). In leafy members, the first fragment (FR1) formed a new gene block together with *nad5* exon 3, *rps13*, and *ccmC* (this block had been rearranged in dodders). The second (FR2) and third (FR3) fragments were still connected in leafy species, with a *ca.* 3.5 kb insertion (2.8 kb of plastid origin) in between; in *Cu. epilinum* and *Cu. europaea*, the insertion was reduced to *ca.* 1.3 kb; while in other dodders, the connection was broken, and FR2 and FR3 had become separated.

### CcmFc encountered fission and a splicing shift

Considering the fast evolution of mitochondrial non-coding regions (Fig. 3), the presence of all three *ccmFc* fragments in Convolvulaceae probably implies they are still functional. We searched for DNA and amino acid sequences of *ccmFc* in known Convolvulaceae genomes to detect potential nuclear transfers, but no high-confidence matches were obtained. We then mapped the rRNA depletion transcriptome of sweet potato to the mitogenome to check for evidence of active expression. All three fragments yielded high expression levels (Fig. 6b, c). FR1 formed a new transcription unit with the downstream *nad5* exon 3, *rps13*, and *ccmC* (Fig. 6b). No splicing sites were found around the 3′-end of FR1, while FR2 and FR3 had reads mapped to both ends (Fig. 6c, d). These results support FR1 becoming an independent gene (hereafter, *ccmFc1*), whereas FR2 and FR3 are two exons of another gene (hereafter, *ccmFc2*). To validate this, we synthesized cDNA of sweet potato, *Dichondra*, *Cu. australis*, *Cu. japonica*, tomato, and *Arabidopsis* from mRNA using reverse transcriptase (RT) and performed PCR analyses. Primer pair “F1 + R1” yielded a band in tomato and *Arabidopsis* but not Convolvulaceae species (Fig. 6e). In contrast, primer pair “F2 + R2” that should amplify a part within the region containing FR2 and FR3 yielded a band for all cDNA templates, serving as a positive control for the quality of the cDNA templates. These results support the occurrence of the fission event deduced from the transcriptome data.

The two exons of *ccmFc2* are still concatenated in most family members but are separated in some dodders (Fig. 6b). The existence of both *cis*- and *trans*-splicing in angiosperm mitogenomes raises the obvious question of how *ccmFc2* mRNA is spliced. The following evidence indicated splicing could be *trans* in all species. On the one hand, the unspliced intron-containing pre-mRNA could be cloned since random primers were used for reverse transcription. Another band fainter in brightness was observed in the gel for tomato and *Arabidopsis*, but not for Convolvulaceae species (Fig. 6e). On the other hand, a *cis*-splicing intron might also be transcribed at a high level (e.g., *cis*-splicing intron of *nad5* in Fig. 6c). However, the transcriptomic mapping of sweet potato displayed a relatively low coverage close to FR2 (Fig. 6c, blue line). Similar results were also obtained from the very recent work of the two dodders *Cu. australis* and *Cu. campestris* [50].

### Coding sequences expanded after fission

**Fig. 7:**
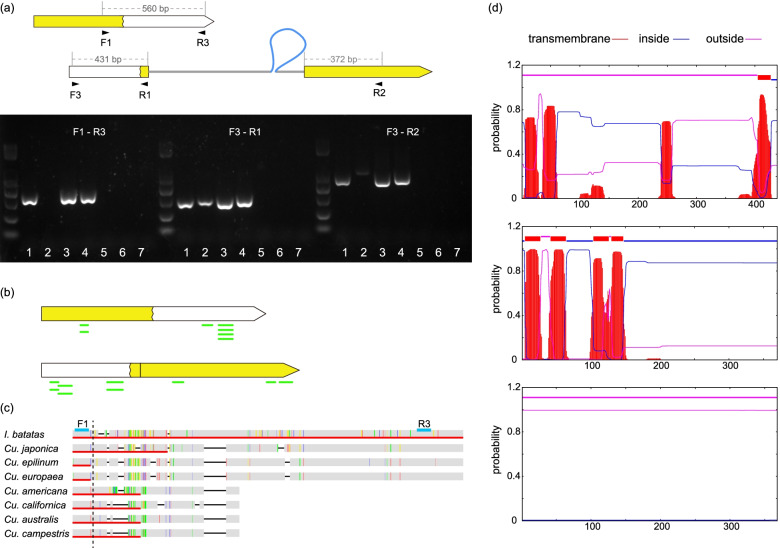
Coding regions of *ccmFc1* and *ccmFc2* expanded after breaking. **a** Position and direction of primers and agarose gel of cDNA confirming gene expansion. Gene lengths referred to sweet potato. Lanes 1 to 7 represent *Cu. australis*, *Cu. japonica*, sweet potato, *Dichondra*, tomato, *Arabidopsis*, and no template. **b** Peptides (shown by short green segments) were identified by mass spectrometry data. **c** Independent nonsense mutations truncated the expansion of *ccmFc1* amino acid sequences in *Cuscuta*. Blue lines indicated the position of F1 and R3 primers. Red lines under the sequence showed coding regions. The vertical black dotted line indicated the breakpoint. **d** Transmembrane helices predicted by TMHMM: top, tomato *ccmFc*; middle, sweet potato *ccmFc1*; bottom, sweet potato *ccmFc2*

Along with the fission, there was an increase in indels and divergent bases in the coding regions of *ccmFc*, which accelerated the substitution rates (see Additional file 3, *ccmFc1* and *ccmFc2*). Additionally, after the break, FR1 was separated from the stop codon. Thus, it required a new stop codon downstream; similarly, FR2 required a new initiation codon upstream. However, we determined that the closest stop and ATG initiation codons were far away, resulting in a *ca.* 185 and 118 aa expansion of *ccmFc1* and *ccmFc2*, respectively. New RNA editing sites were absent in the expanded genic regions. We cloned cDNA to confirm the gene expansion (Fig. 7a). The expansions were further confirmed by the sweet potato mass spectrometry data, with multiple hits detected during peptide identification (Fig. 7b). In dodders, *ccmFc1* gained independent nonsense mutations shortening its length back to the breakpoint (Fig. 7c).

*CcmFc* is localized to the mitochondrial inner membrane and contains multiple predicted transmembrane helices in both exons (Fig. 7d [48];. However, in sweet potato, our predictions for *ccmFc1* and *ccmFc2* (performed by TMHMM Server 2.0 [51]) revealed four transmembrane helices in *ccmFc1* but none in *ccmFc2* (Fig. 7d). The breaks and expansions might have altered the protein structure.

### Cis- and trans-splicing may not differ much

**Fig. 8:**
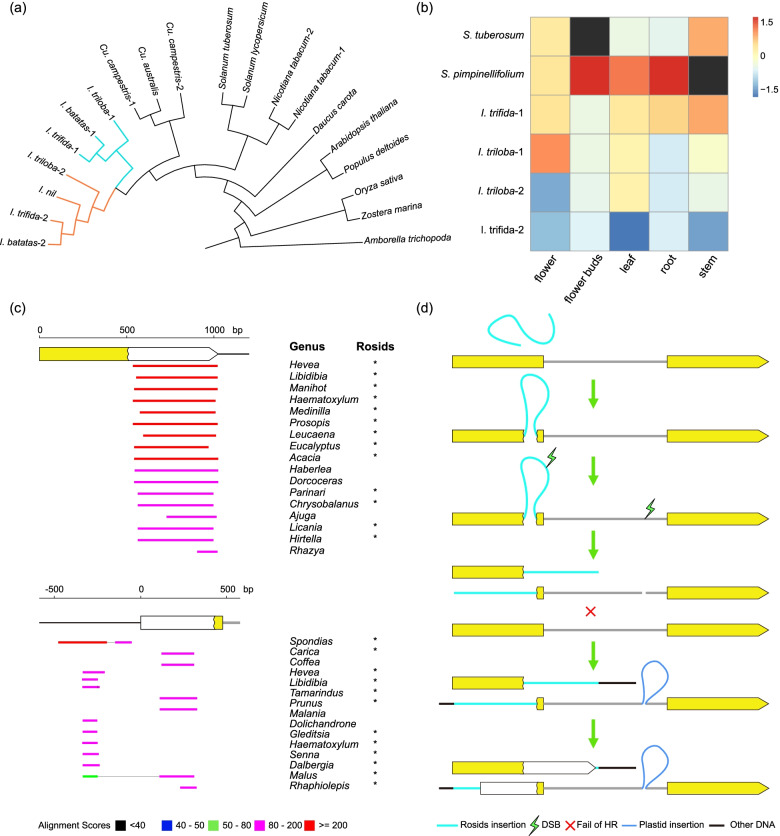
Phylogeny and expression of *WTF9* and a potential evolutionary history of *ccmFc* in Convolvulaceae. **a** Phylogeny of *WTF9* in angiosperms. Colored branches in *Ipomoea* showed duplication. **b** *WTF9* expression level in *Solanum* and *Ipomoea*. Black boxes indicated missing data. **c** BLASTN searches of the downstream of FR1 and upstream of FR2 against NCBI *nt* database. Top hits were mainly from rosids (*) after masked hits from Convolvulaceae and other parasitic plants. **d** A potential evolutionary trajectory of *ccmFc* in Convolvulaceae

The mechanism of the shift from *cis*- to *trans*-splicing remains unclear. What’s This Factor 9 (WTF9), a nuclear-encoded plant organelle RNA recognition protein, can bind directly to the 48 nucleotides of the *ccmFc* intron to regulate *cis*-splicing [49, 52]. The molecular chaperone heat shock protein 60 (HSP60) interacts with WTF9 during this process [52]. To detect potential changes caused by the *trans*-splicing shift, we used *Arabidopsis WTF9* and *HSP60* as references to search for homologs in genomes of Convolvulaceae and other angiosperms. *WTF9* is retained in all species of Convolvulaceae and even duplicated in *Ipomoea* (Fig. 8a). We checked the expression level of *WTF9* in *I. trifida*, *I. triloba*, and two species of Solanaceae, potato and *S. pimpinellifolium*. The expression pattern of *Ipomoea WTF9-1* resembled that of *Solanum* (Fig. 8b). A similar situation was observed for *HSP60* (Additional file 6). After the splicing shift, the two intronic fragments were almost entirely preserved in all species, including the 48-nucleotide binding region (except *Cu. america*, which has a 28 bp insertion in the binding region). Based on these, we speculated that the mechanism between *cis*- and *trans*-splicing might be similar, but further research is needed to test this hypothesis.

### Variations in Convolvulaceae CcmFc is unprecedented among angiosperms

Under selection pressure and homologous recombination (HR) repair, plant mitochondrial genes evolve more slowly than other genome compartments [53, 54]. The mitogenomic structure in angiosperms is exceptionally dynamic, while gene sequences and structure, as well as methods of splicing, are well conserved [55, 56]. *Trans*-splicing has evolved independently several times in land plants, such as in lycophytes and gymnosperms [57–60]. In angiosperms, the emergence of *trans*-splicing was less frequent. Aside from *nad1*, *nad2*, and *nad5*, only *cox2* in *Allium* was reported to undergo *trans*-splicing [61–64]. Our discovery of *ccmFc* in Convolvulaceae represents a novel type of *trans*-splicing in angiosperms. Gene fission has also occurred several times in plant mitogenomes, such as *ccmFc* in *Marchantia* and *ccmFn* in Brassicaceae, *Allium*, and *Trifolium*, caused either by deletion-based frameshift mutation or rearrangements [65–68]. As far as we know, fission followed by expansions found in this study is the first time reported in mitogenomes. Among them, the *ccmFc* in Convolvulaceae might be the most complicated, integrating gene fission, splicing shift, and expansions. We also searched the downstream of FR1 and upstream of FR2 against the NCBI *nt* database, finding that both were most similar to the mitochondrial sequences of rosids after masked hits of Convolvulaceae (Fig. 8c; Additional file 7). We hypothesized that a long mitochondrial sequence from rosids inserted into the *ccmFc* gene of Convolvulaceae common ancestor occasionally (Fig. 8d). Then double-strand breaks (DSBs [69];) occurred inside the insertion and the intron. The long insertion could make it difficult to repair the mutation through HR. Imprecise non-homologous repair finally created the gene fission and brought the plastid insertion inside the intron. The latter may have interrupted the standard secondary structure of the group II intron, resulting in a shift from *cis*-splicing to *trans*. HGTs are widespread among mitogenomes but mostly non-functional [70, 71]. In some cases, HGT can replace native genes completely or partially [41, 72]. The chimeric *ccmFc1* and *ccmFc2* in Convolvulaceae fuse novel HGT and native coding sequences, representing a novel manner of how HGT functionally impacts the organism.

An immediate question is how these changes would modify the structure of the CCM complex or even the efficiency of oxidative phosphorylation. The elevated substitution rates and potential changes in transmembrane helices may have affected the protein structure. Recent studies have shown that modifying cytochrome *c* maturation can optimize extracellular electron transfer for higher electron flux in engineered *Escherichia coli* [73]. Our results provide an example of plant respiratory diversity, which may have implications for the engineering of plant respiration in the future.

## Conclusions

Th*e* family Convolvulaceae is famous for its extensive uses, diverse morphologies, and different trophic modes. In this study, our family-scale analyses revealed that the same amazement also exists in their genetic materials. Plastids (including chloroplasts) and mitochondria play crucial roles in cellular energy supply and metabolism. Normally the structure and genes of plastome and coding sequences of mitogenome are well-conserved. However, it has some changes in Convolvulaceae. Even in green leafy members, the IR boundaries are all atypical, and uncommon features such as intron losses, nuclear transfer (*infA*), duplications, and foreign insertions also exist. The mitogenome of *Cu. japonica* was significantly inflated by HGT-like sequences, whereas other dodders preserved in a small size like their leafy relatives—the mitogenome evolves divergently in dodders. Besides, dodder mitochondrial genes have more losses and higher substitution rates, but no HGT events. The most notable variation is the mitochondrial *ccmFc*. It is highly conserved in other plants but likely encountered gene fission, splicing shift, and coding expansion in the common ancestor of Convolvulaceae, which is unique in angiosperms. Nuclear genes involved in the splicing process seems still functional, raising the question of what differs between *cis*- and *trans*-splicing on mechanism. These unusual changes of *ccmFc* were likely caused by HGT insertion followed by double-strand breaks and imprecise repairs. Our data provide valuable genetic resources for studying the evolution and phylogeny of Convolvulaceae and plant parasitism. The results presented in this study are also helpful to understand the diversification of mitochondrial complexes and gene innovation.

## Methods

### Sampling and sequencing

Accessions of 12 Convolvulaceae species were collected from different places in China (Additional file 8). Sequencing libraries were prepared using a NEB Next® Ultra DNA Library Prep Kit, then sequenced on the Illumina Hiseq 4000 platform to generate PE150 reads. *Dichondra micrantha* was also sequenced using the Oxford Nanopore promethION platform. Raw sequencing data for the other 10 species were obtained from NCBI SRA (https://www.ncbi.nlm.nih.gov/sra). Details of the reads are provided in Additional file 9.

### Genome assembly and annotation

Short reads were filtered using TRIMMOMATIC v0.36 [74]. Plastomes were assembled using GetOrganelle [75] and annotated using *I. nil* (GenBank: NC_031159) as the reference. The assembly of mitogenomes was as described in our previous work [47]. Briefly, de novo assemblies of short and long reads were performed using SPAdes v3.13.1 [76] and FLYE v2.8.3 [77], respectively. Then mitochondrial contigs were identified from total contigs by BLASTN against the *I. nil* mitogenome. These contigs were connected and/or extended manually in GENEIOUS R10 (Biomatters, Inc.) by mapping reads back and checking both ends. Long repeats and plastid insertions were the two major challenges of the assembly. Repeat regions were resolved using short-read sequencing coverage; plastid insertions were resolved based on the locations of their plastid counterparts. The mitogenome could get one or several circles after the repeats and plastid insertions were properly determined. *Cu. japonica* yielded one linear chromosome since repeats at both ends could not be connected. The four dodders from subgenus *Grammica* (*Cu. americana*, *Cu. australis*, *Cu. californica*, and *Cu. campestris*) only obtained draft mitogenomes because of the large amounts of repeats. Putative mitochondrial protein-coding and rRNA genes were annotated based on similarity to known mitochondrial genes. tRNAs were predicted using tRNAscan-SE v2.0 [78]. Plastid insertion and repeat length were determined by BLASTN v2.10.0+ [79] against Convolvulaceae plastomes (identity > 90%) and itself (identity > 95%), respectively. Common sequences were determined initially by BLASTN (word size 16, *e*-value 1e-5), and then counted using custom scripts.

### Analyses of genome synteny and Genus-specific sequences

Plastome (IR removed) and mitogenome syntenies were plotted using Python version MCscan of JCVI 192 utility libraries v1.1.17 [80]. The DNA sequences were first divided into 50 bp fragments, and these fragments were then forced to be used as “genes” to search for homologous regions. Genus-specific sequences (GSS) meant sequences that shared no homology with other genera. For species in *Ipomoea* and *Cuscuta*, GSS also included the species-specific sequences. GSS were identified by searching each mitogenome against other Convolvulaceae mitogenomes, with *e*-value of 1e-5 and word size of 16. The GSS of *Cu. japonica*, *Dichondra*, and *Evolvulus* were searched against NCBI *nt* database to find the most potential donors, with only hits longer than 100 bp considered. Subsequently, the best matches were 184 grouped into orders (Additional file 3). Orders with total length >5 kb were plotted as pie charts in R (https://www.r-project.org/). To test whether other dodders have experienced expansion, high-confidence HGT-like sequences of *Cu. japonica* (best hits >500 bp) were searched against other dodders, with the percentage of the coverage calculated. Finally, the heatmaps were generated using PHEATMAP (https://github.com/raivokolde/pheatmap) in R.

### Phylogenetic analyses

Potential HGT events of the mitochondrial genes were detected. CDS of Convolvulaceae and another 42 seed plants were aligned using MAFFT v7 [81] with the “auto” mode. Then ML trees were built using IQTREE v1.6.12 [82] with parameter *-bb 1000 -m GTR+G4+F -me 0.0001 runs 5*. The trees were plotted using FIGTREE v.1.4.2 (http://tree.bio.ed.ac.uk/software/figtree/). GenBank accessions were listed behind the species on the trees (Additional file 3).

The plastid CDS were obtained from our assemblies and *Ipomoea batatas* (GenBank: NC_026703), *I. nil* (NC_031159), *I. trifida* (NC_034670), *I. triloba* (NC_037913), *Cressa cretica* (NC_035516), *Cuscuta australis* (NC_045885), *Cu. campestris* (NC_052920), *Convolvulus arvensis* (MW054627), *Operculina macrocarpa* (KF242502), *Nicotiana tabacum* (Z00044), *Solanum melongena* (MN218080), and *Solanum lycopersicum* (NC_007898). Mitochondrial CDS were obtained from our assemblies and *I. nil* (AP017303), *N. tabacum* (NC_006581), *S. lycopersicum* (NC_035963), and *S. melongena* (NC_050334). RNA editing sites were predicted using the PREP website (Mower, 2009) and removed manually. The nuclear 45S were obtained from *de novo* contigs and *Nicotiana benthamiana* (KP824745). Aligned plastid and mitochondrial (*rps7* was excluded from an HGT) CDS were concatenated into one matrix, respectively. Then the three datasets (plastid CDS, mitochondrial CDS, and nuclear 45S) were built the ML trees as mentioned above. Solanaceae were always used as outgroups.

### Peptide identification from published proteomics data

Protein mass spectrometry data for sweet potato (*I. batatas*) were downloaded from ProteomeXchange PXD012999 [83]. A Trans-Proteomic Pipeline (TPP) v5.2.0 [84] comet search was used for peptide identification with default parameters.

### Gene family analyses

Amino acid sequences of *Arabidopsis thaliana WTF9* (AT3G24320), *HSP60-2* (AT2G33210), *HSP60-3A* (AT3G13860), and *HSP60-3B* (AT3G23990) were used as references to search for homologs in Convolvulaceae, including sweet potato [85], *I. trifida* and *I. triloba* [86], *I. nil* [15], *Cuscuta australis* [19], and *Cu. campestris* [20], and other angiosperms, including *Nicotiana tabacum* [87], *Amborella trichopoda* v1.0 [88], *Daucus carota* v2.0 [89], *Populus trichocarpa* v3.0 [90], *Oryza sativa* v7 [91], tomato *Solanum lycopersicum* iTAG2.4 [92], potato *Solanum tuberosum* v4.03 [93], and *Zostera marina* v2.2 [94] (the last seven were downloaded from Phytozome v13: https://phytozome.jgi.doe.gov/). The amino acid sequences were then aligned through MAFFT, and trees were then reconstructed using IQTREE (parameter: *-bb 1000 -m LG+F+G4 runs 10 -me 0.0001*).

The expression level data of *Ipomoea trifida* and *I. triloba* were obtained from Sweetpotato Genomics Resource [86]; those of *Solanum pimpinellifolium* were from the Tomato Functional Genomics Database [95], and those of potato were from SpudDB [96]. Heatmaps were plotted using PHEATMAP in R (https://r-charts.com/correlation/pheatmap/) by normalizing each gene with Z-score.

### Expression examination

Transcriptomes enriched with oligo-dT would introduce bias since organelle transcripts do not generally contain a polyA tail [97]. Based on that, sweet potato rRNA-depletion transcriptome data (SRA: SRR10538086 [98]) were employed and were mapped to the mitogenome to check the expression.

Fresh leaves of sweet potato, *Dichondra*, tomato, *Arabidopsis*, and fresh vines of *Cuscuta australis* and *Cu. japonica* were used for RNA extraction using an Omega RNA isolation kit and reverse-transcribed into cDNA using random primers. Primers used for RT-PCR were designed using GENEIOUS (Additional file 10).

## Supplementary Information

**Additional file 1.** Plastome content. The black, grey and blank cells represent intact, pseudo and missing, respectively. Coding genes with disrupted reading frames, premature stop codons, or non-triplet frameshifts were annotated as pseudogenes.

**Additional file 2.** Mitogenome content. The black, grey and blank cells represent intact, pseudo and missing, respectively. Coding genes with disrupted reading frames, premature stop codons, or non-triplet frameshifts were annotated as pseudogenes.

**Additional file 3.** Most mitochondrial genes in dodders.

**Additional file 4.** BLASTN results of Cu. japonica genus- and species-specific sequences.

**Additional file 5.** Plastid CDS, Mitochondrial CDS, and Nuclear 45S.

**Additional file 6 **Similar situation observed for *HSP60.*

**Additional file 7.** BLASTN searches of the downstream of FR1 and upstream of FR2. Erycibe was used as the query. Parameter: word_size 16, e-value 1e-5.

**Additional file 8.** Collections of materials.

**Additional file 9.** Data used for organelle genome assemblies.

**Additional file 10.** Primers used to confirm gene fission and expansion.

## Data Availability

Raw reads have been deposited on NCBI under BioProject PRJNA737311 and accession SRR14812959 - SRR14812971 (https://www.ncbi.nlm.nih.gov/bioproject/PRJNA737311) [99]. The assembled mitogenomes and plastomes have been deposited to NCBI under accessions MZ240723 - MZ240750 and BK059236 - BK059244 (TPA) and CNGBdb under project CNP0001927 and accessions N_000011218 - N_000011230 and N_000011273 - N_000011309 (https://db.cngb.org/search/project/CNP0001927). The used scripts can be found in Github (https://github.com/fengyanlei33/Convolvulaceae-mitogenome-project) [100].

## Abbreviations

IR: Inverted repeat
LSC: Long single-copy
SSC: Short single-copy
HGT: Horizontal gene transfer
GSS: Genus- or/and species-specific sequences
CDS: Coding sequences
ML: Maximum-likelihood
PIS: Parsimony informative sites
CCM: Cytochrome *c* maturation
FR1: The first fragment
FR2: The second fragment
FR3: The third fragment
RT: Reverse transcriptase
WTF9: What’s This Factor 9
HSP60: Heat shock protein 60
HR: Homologous recombination
DSBs: Double-strand breaks
