# Supplementary material for: Unprecedented organelle genomic variations in morning glories reveal independent evolutionary scenarios of parasitic plants and the diversification of plant mitochondrial complexes
Source: BMC Biol. 2022 Feb 16;20:49. doi: 10.1186/s12915-022-01250-1 (PMC8851834; doi:10.1186/s12915-022-01250-1)
Supplement: Supplementary file 3 — Additional file 3. Most mitochondrial genes in dodders. [file 12915_2022_1250_MOESM3_ESM.pdf]

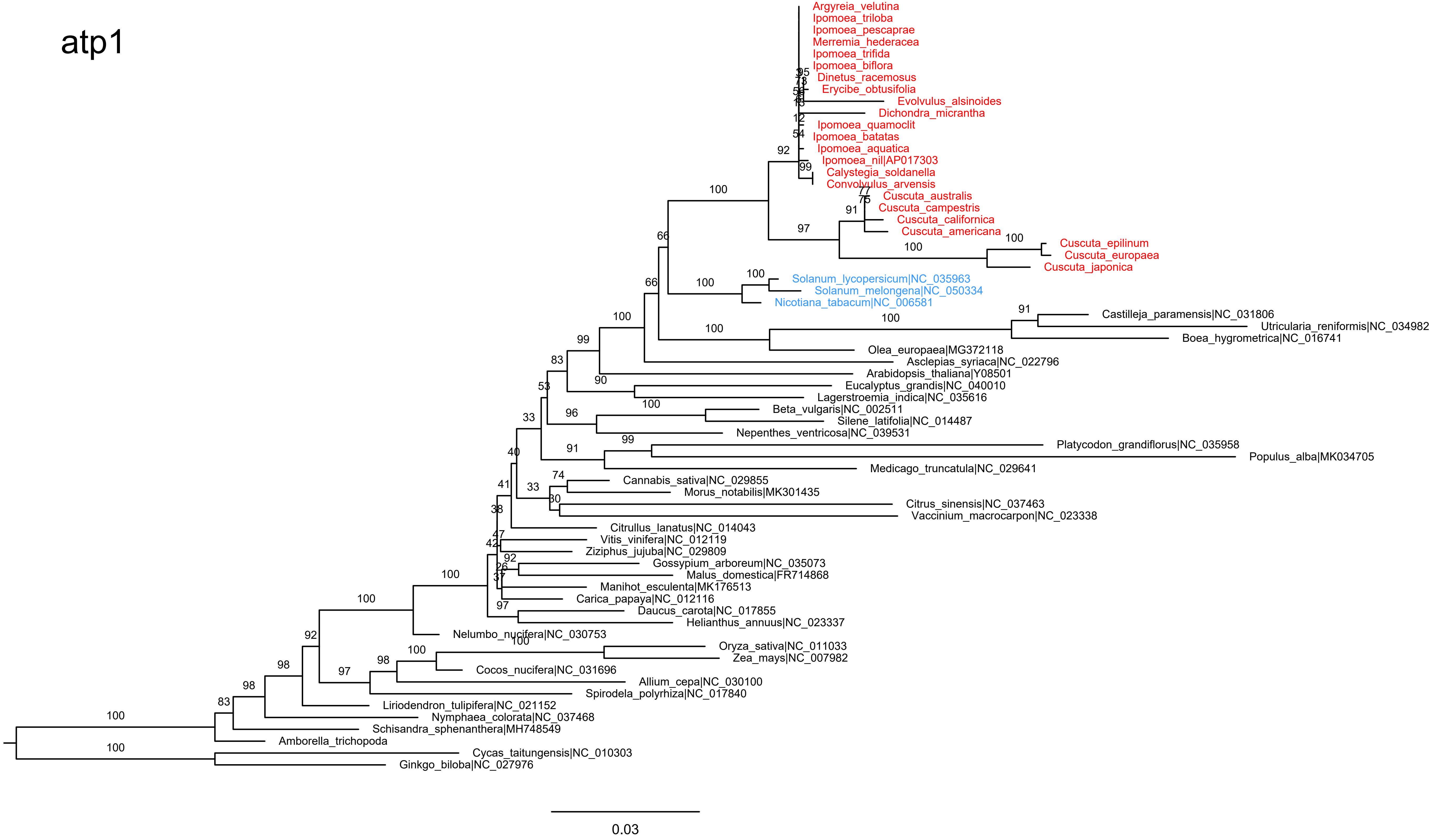
atp1
Argyreia_velutina
Ipomoea_triloba
Ipomoea_pescaprae
Merremia_hederacea
Ipomoea_trifida
Ipomoea_biflora
Dinetus_racemosus
Erycibe_obtusifolia
Evolvulus_alsinoides
Dichondra_micrantha
Ipomoea_quamoclit
Ipomoea_batatas
Ipomoea_aquatica
Ipomoea_nil|AP017303
Calystegia_soldanella
Convolvulus_arvensis
Cuscuta_australis
Cuscuta_campestris
Cuscuta_californica
Cuscuta_americana
Cuscuta_epilinum
Cuscuta_europaea
Cuscuta_japonica
Solanum_lycopersicum|NC_035963
Solanum_melongena|NC_050334
Nicotiana_tabacum|NC_006581
Castilleja_paramensis|NC_031806
Utricularia_reniformis|NC_034982
Boea_hygrometrica|NC_016741
Olea_europaea|MG372118
Asclepias_syriaca|NC_022796
Arabidopsis_thaliana|Y08501
Eucalyptus_grandis|NC_040010
Lagerstroemia_indica|NC_035616
Beta_vulgaris|NC_002511
Silene_latifolia|NC_014487
Nepenthes_ventricosa|NC_039531
Platycodon_grandiflorus|NC_035958
Populus_alba|MK034705
Medicago_truncatula|NC_029641
Cannabis_sativa|NC_029855
Morus_notabilis|MK301435
Citrus_sinensis|NC_037463
Vaccinium_macrocarpon|NC_023338
Citrullus_lanatus|NC_014043
Vitis_vinifera|NC_012119
Ziziphus_jujuba|NC_029809
Gossypium_arboreum|NC_035073
Malus_domestica|FR714868
Manihot_esculenta|MK176513
Carica_papaya|NC_012116
Daucus_carota|NC_017855
Helianthus_annuus|NC_023337
Nelumbo_nucifera|NC_030753
Oryza_sativa|NC_011033
Zea_mays|NC_007982
Cocos_nucifera|NC_031696
Allium_cepa|NC_030100
Spirodela_polyrhiza|NC_017840
Liriodendron_tulipifera|NC_021152
Nymphaea_colorata|NC_037468
Schisandra_sphenanthera|MH748549
Amborella_trichopoda
Cycas_taitungensis|NC_010303
Ginkgo_biloba|NC_027976
0.03

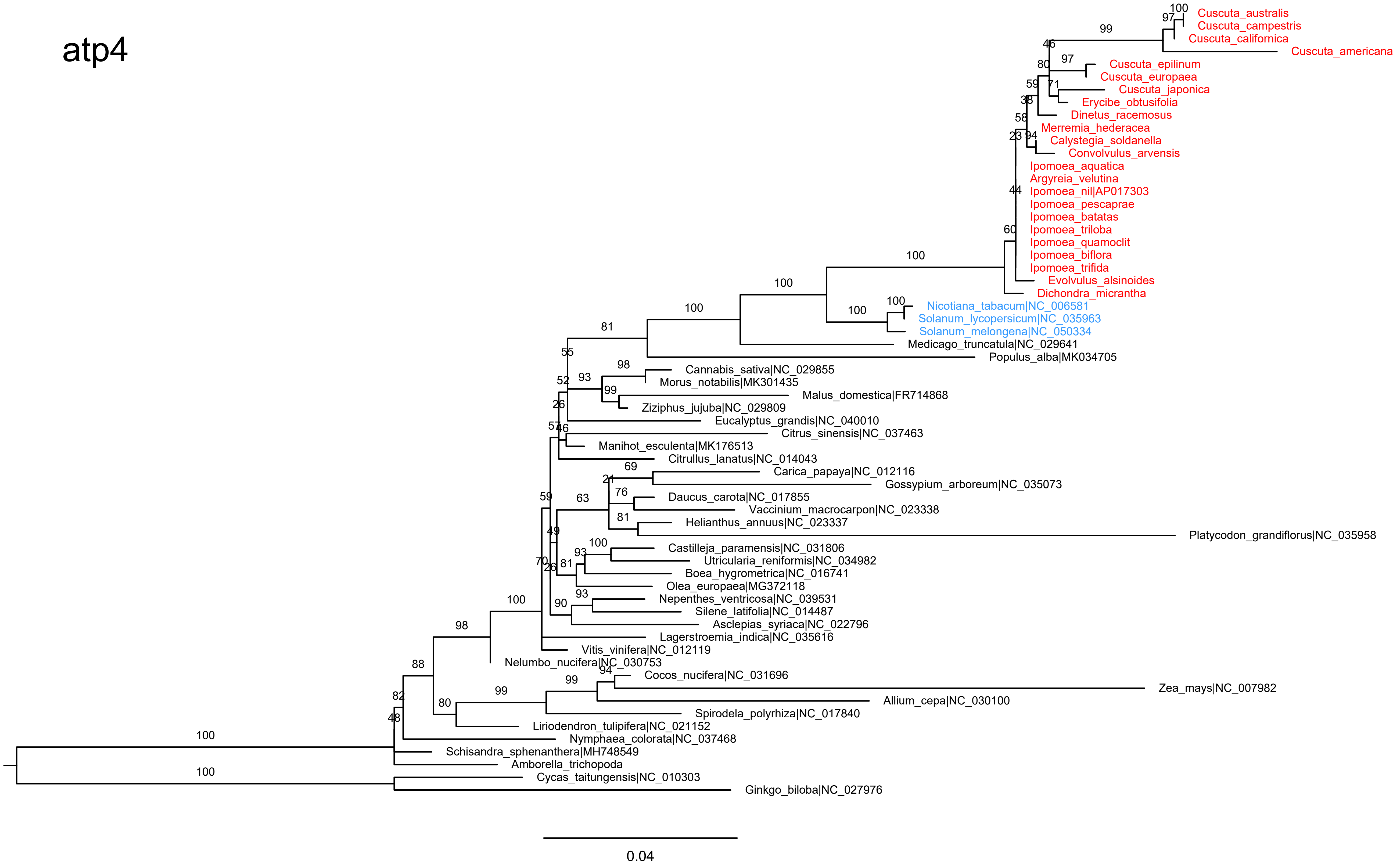

atp4
Cuscuta_australis
Cuscuta_campestris
Cuscuta_californica
Cuscuta_americana
Cuscuta_epilinum
Cuscuta_europaea
Cuscuta_japonica
Erycibe_obtusifolia
Dinetus_racemosus
Merremia_hederacea
Calystegia_soldanella
Convolvulus_arvensis
Ipomoea_aquatica
Argyreia_velutina
Ipomoea_nil|AP017303
Ipomoea_pescaprae
Ipomoea_batatas
Ipomoea_triloba
Ipomoea_quamoclit
Ipomoea_biflora
Ipomoea_trifida
Evolvulus_alsinoides
Dichondra_micrantha
Nicotiana_tabacum|NC_006581
Solanum_lycopersicum|NC_035963
Solanum_melongena|NC_050334
Medicago_truncatula|NC_029641
Populus_alba|MK034705
Cannabis_sativa|NC_029855
Morus_notabilis|MK301435
Malus_domestica|FR714868
Ziziphus_jujuba|NC_029809
Eucalyptus_grandis|NC_040010
Citrus_sinensis|NC_037463
Manihot_esculenta|MK176513
Citrullus_lanatus|NC_014043
Carica_papaya|NC_012116
Gossypium_arboreum|NC_035073
Daucus_carota|NC_017855
Vaccinium_macrocarpon|NC_023338
Helianthus_annuus|NC_023337
Platycodon_grandiflorus|NC_035958
Castilleja_paramensis|NC_031806
Utricularia_reniformis|NC_034982
Boea_hygrometrica|NC_016741
Olea_europaea|MG372118
Nepenthes_ventricosa|NC_039531
Silene_latifolia|NC_014487
Asclepias_syriaca|NC_022796
Lagerstroemia_indica|NC_035616
Vitis_vinifera|NC_012119
Nelumbo_nucifera|NC_030753
Cocos_nucifera|NC_031696
Zea_mays|NC_007982
Allium_cepa|NC_030100
Spirodela_polyrhiza|NC_017840
Liriodendron_tulipifera|NC_021152
Nymphaea_colorata|NC_037468
Schisandra_sphenanthera|MH748549
Amborella_trichopoda
Cycas_taitungensis|NC_010303
Ginkgo_biloba|NC_027976
0.04

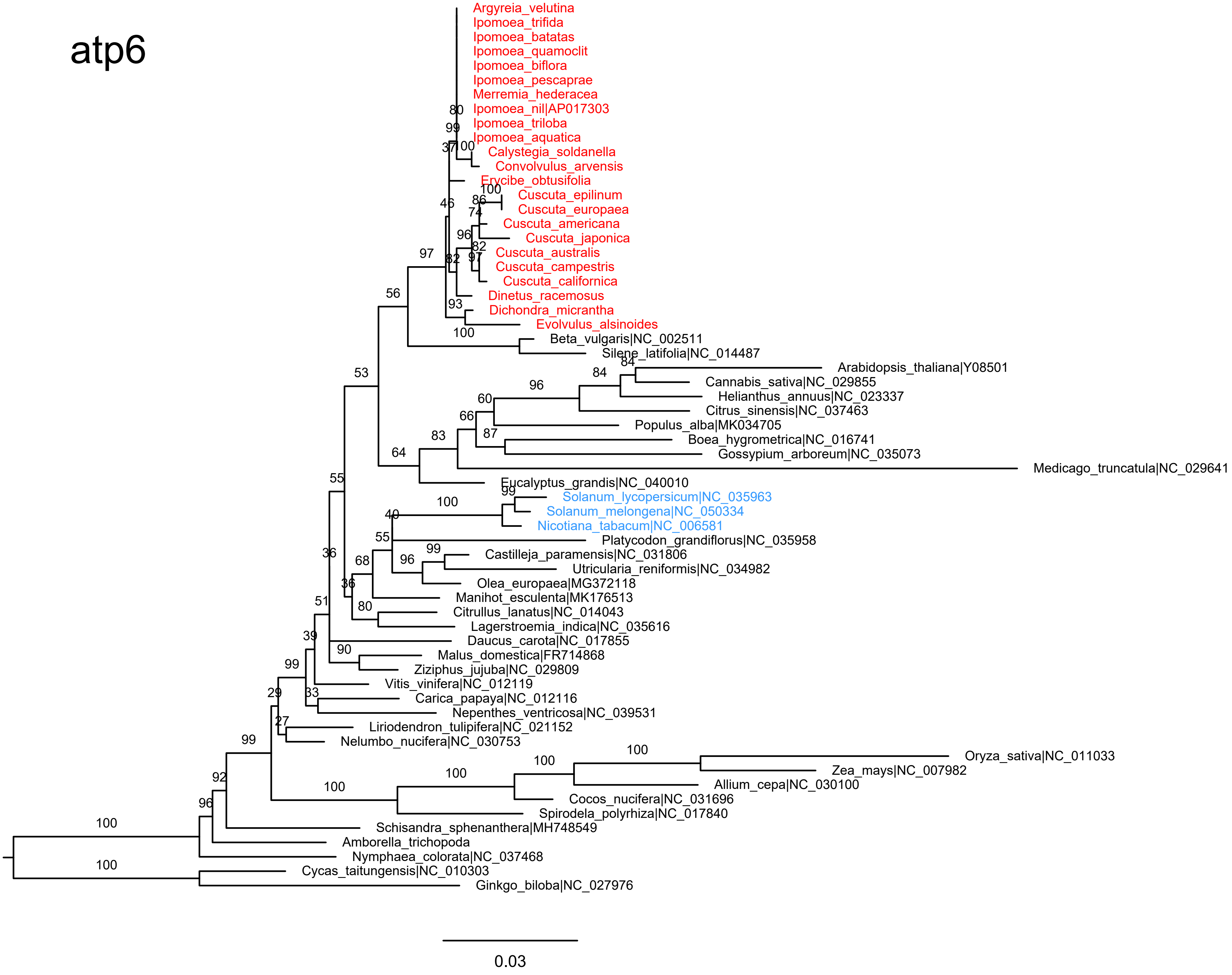
atp6
Argyreia_velutina
Ipomoea_trifida
Ipomoea_batatas
Ipomoea_quamoclit
Ipomoea_biflora
Ipomoea_pescaprae
Merremia_hederacea
Ipomoea_nil|AP017303
Ipomoea_triloba
Ipomoea_aquatica
Calystegia_soldanella
Convolvulus_arvensis
Erycibe_obtusifolia
Cuscuta_epilinum
Cuscuta_europaea
Cuscuta_americana
Cuscuta_japonica
Cuscuta_australis
Cuscuta_campestris
Cuscuta_californica
Dinetus_racemosus
Dichondra_micrantha
Evolvulus_alsinoides
Beta_vulgaris|NC_002511
Silene_latifolia|NC_014487
Arabidopsis_thaliana|Y08501
Cannabis_sativa|NC_029855
Helianthus_annuus|NC_023337
Citrus_sinensis|NC_037463
Populus_alba|MK034705
Boea_hygrometrica|NC_016741
Gossypium_arboreum|NC_035073
Medicago_truncatula|NC_029641
Eucalyptus_grandis|NC_040010
Solanum_lycopersicum|NC_035963
Solanum_melongena|NC_050334
Nicotiana_tabacum|NC_006581
Platycodon_grandiflorus|NC_035958
Castilleja_paramensis|NC_031806
Utricularia_reniformis|NC_034982
Olea_europaea|MG372118
Manihot_esculenta|MK176513
Citrullus_lanatus|NC_014043
Lagerstroemia_indica|NC_035616
Daucus_carota|NC_017855
Malus_domestica|FR714868
Ziziphus_jujuba|NC_029809
Vitis_vinifera|NC_012119
Carica_papaya|NC_012116
Nepenthes_ventricosa|NC_039531
Liriodendron_tulipifera|NC_021152
Nelumbo_nucifera|NC_030753
Oryza_sativa|NC_011033
Zea_mays|NC_007982
Allium_cepa|NC_030100
Cocos_nucifera|NC_031696
Spirodela_polyrhiza|NC_017840
Schisandra_sphenanthera|MH748549
Amborella_trichopoda
Nymphaea_colorata|NC_037468
Cycas_taitungensis|NC_010303
Ginkgo_biloba|NC_027976
0.03

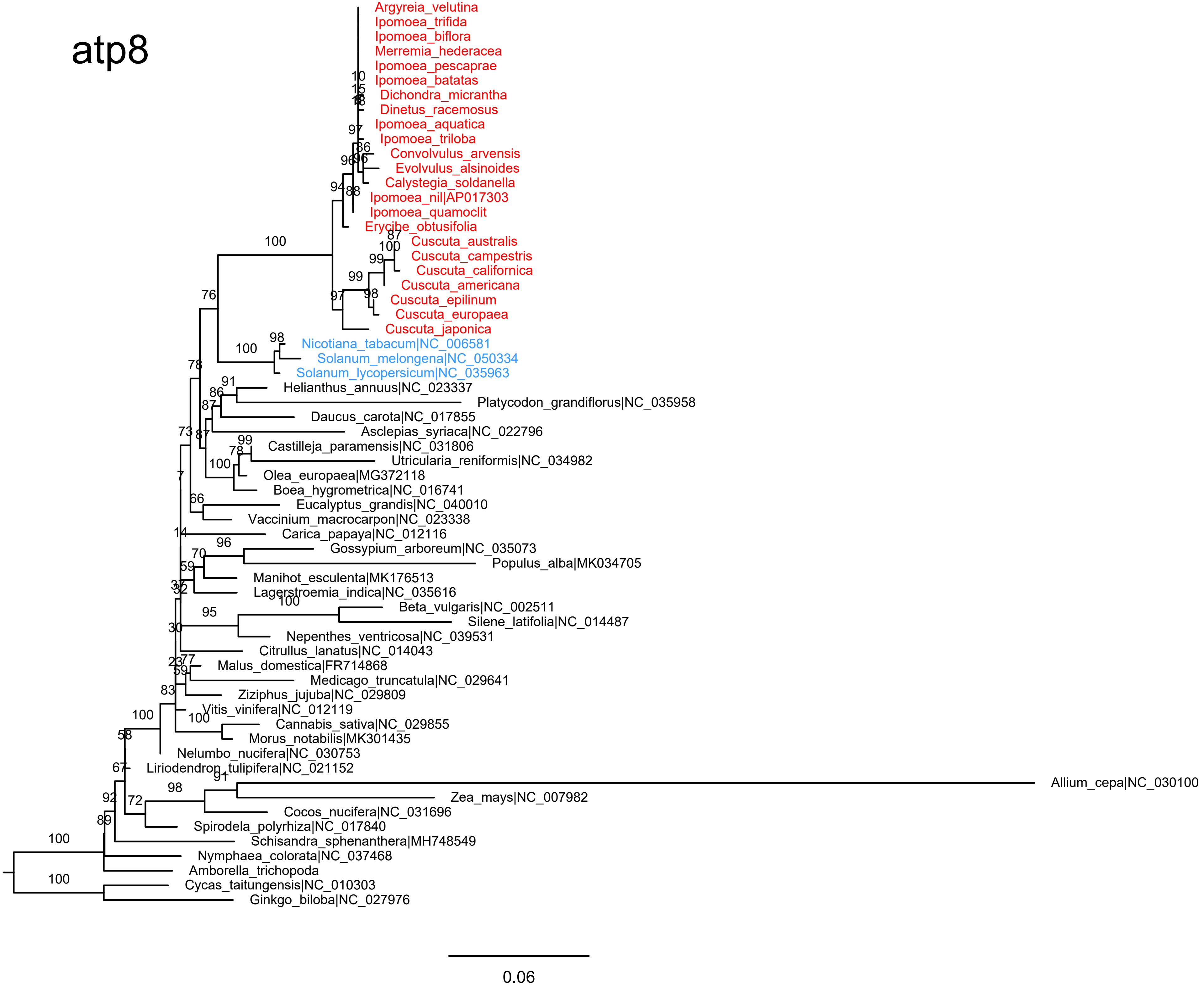
atp8
Argyreia_velutina
Ipomoea_trifida
Ipomoea_biflora
Merremia_hederacea
Ipomoea_pescaprae
Ipomoea_batatas
Dichondra_micrantha
Dinetus_racemosus
Ipomoea_aquatica
Ipomoea_triloba
Convolvulus_arvensis
Evolvulus_alsinoides
Calystegia_soldanella
Ipomoea_nil|AP017303
Ipomoea_quamoclit
Erycibe_obtusifolia
Cuscuta_australis
Cuscuta_campestris
Cuscuta_californica
Cuscuta_americana
Cuscuta_epilinum
Cuscuta_europaea
Cuscuta_japonica
Nicotiana_tabacum|NC_006581
Solanum_melongena|NC_050334
Solanum_lycopersicum|NC_035963
Helianthus_annuus|NC_023337
Platycodon_grandiflorus|NC_035958
Daucus_carota|NC_017855
Asclepias_syriaca|NC_022796
Castilleja_paramensis|NC_031806
Utricularia_reniformis|NC_034982
Olea_europaea|MG372118
Boea_hygrometrica|NC_016741
Eucalyptus_grandis|NC_040010
Vaccinium_macrocarpon|NC_023338
Carica_papaya|NC_012116
Gossypium_arboreum|NC_035073
Populus_alba|MK034705
Manihot_esculenta|MK176513
Lagerstroemia_indica|NC_035616
Beta_vulgaris|NC_002511
Silene_latifolia|NC_014487
Nepenthes_ventricosa|NC_039531
Citrullus_lanatus|NC_014043
Malus_domestica|FR714868
Medicago_truncatula|NC_029641
Ziziphus_jujuba|NC_029809
Vitis_vinifera|NC_012119
Cannabis_sativa|NC_029855
Morus_notabilis|MK301435
Nelumbo_nucifera|NC_030753
Liriodendron_tulipifera|NC_021152
Allium_cepa|NC_030100
Zea_mays|NC_007982
Cocos_nucifera|NC_031696
Spirodela_polyrhiza|NC_017840
Schisandra_sphenanthera|MH748549
Nymphaea_colorata|NC_037468
Amborella_trichopoda
Cycas_taitungensis|NC_010303
Ginkgo_biloba|NC_027976
0.06

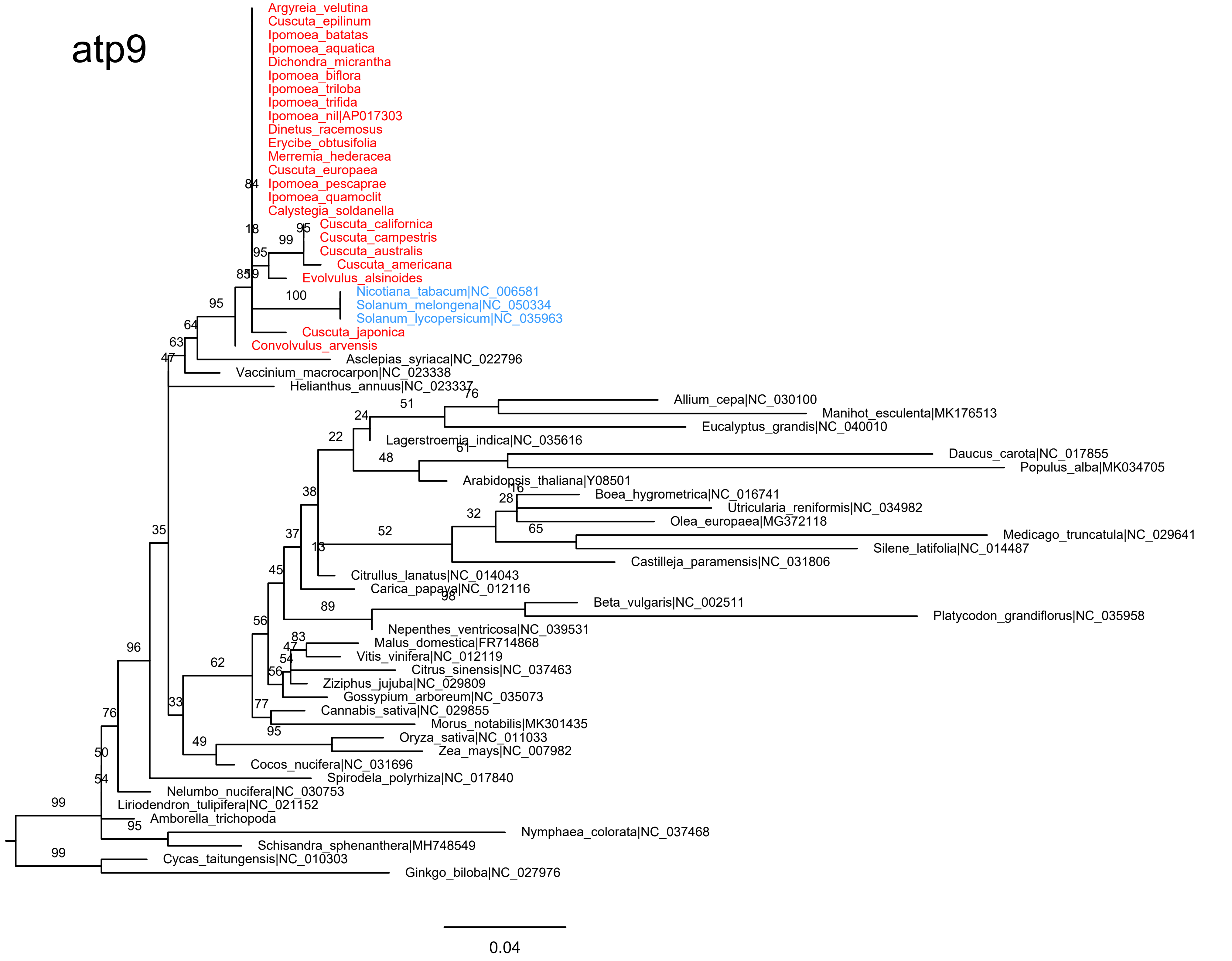
atp9
Argyreia_velutina
Cuscuta_epilinum
Ipomoea_batatas
Ipomoea_aquatica
Dichondra_micrantha
Ipomoea_biflora
Ipomoea_triloba
Ipomoea_trifida
Ipomoea_nil|AP017303
Dinetus_racemosus
Erycibe_obtusifolia
Merremia_hederacea
Cuscuta_europaea
Ipomoea_pescaprae
Ipomoea_quamoclit
Calystegia_soldanella
Cuscuta_californica
Cuscuta_campestris
Cuscuta_australis
Cuscuta_americana
Evolvulus_alsinoides
Nicotiana_tabacum|NC_006581
Solanum_melongena|NC_050334
Solanum_lycopersicum|NC_035963
Cuscuta_japonica
Convolvulus_arvensis
Asclepias_syriaca|NC_022796
Vaccinium_macrocarpon|NC_023338
Helianthus_annuus|NC_023337
Allium_cepa|NC_030100
Manihot_esculenta|MK176513
Eucalyptus_grandis|NC_040010
Lagerstroemia_indica|NC_035616
Daucus_carota|NC_017855
Populus_alba|MK034705
Arabidopsis_thaliana|Y08501
Boea_hygrometrica|NC_016741
Utricularia_reniformis|NC_034982
Olea_europaea|MG372118
Medicago_truncatula|NC_029641
Silene_latifolia|NC_014487
Castilleja_paramensis|NC_031806
Citrullus_lanatus|NC_014043
Carica_papaya|NC_012116
Beta_vulgaris|NC_002511
Platycodon_grandiflorus|NC_035958
Nepenthes_ventricosa|NC_039531
Malus_domestica|FR714868
Vitis_vinifera|NC_012119
Citrus_sinensis|NC_037463
Ziziphus_jujuba|NC_029809
Gossypium_arboreum|NC_035073
Cannabis_sativa|NC_029855
Morus_notabilis|MK301435
Oryza_sativa|NC_011033
Zea_mays|NC_007982
Cocos_nucifera|NC_031696
Spirodela_polyrhiza|NC_017840
Nelumbo_nucifera|NC_030753
Liriodendron_tulipifera|NC_021152
Amborella_trichopoda
Nymphaea_colorata|NC_037468
Schisandra_sphenanthera|MH748549
Cycas_taitungensis|NC_010303
Ginkgo_biloba|NC_027976
0.04

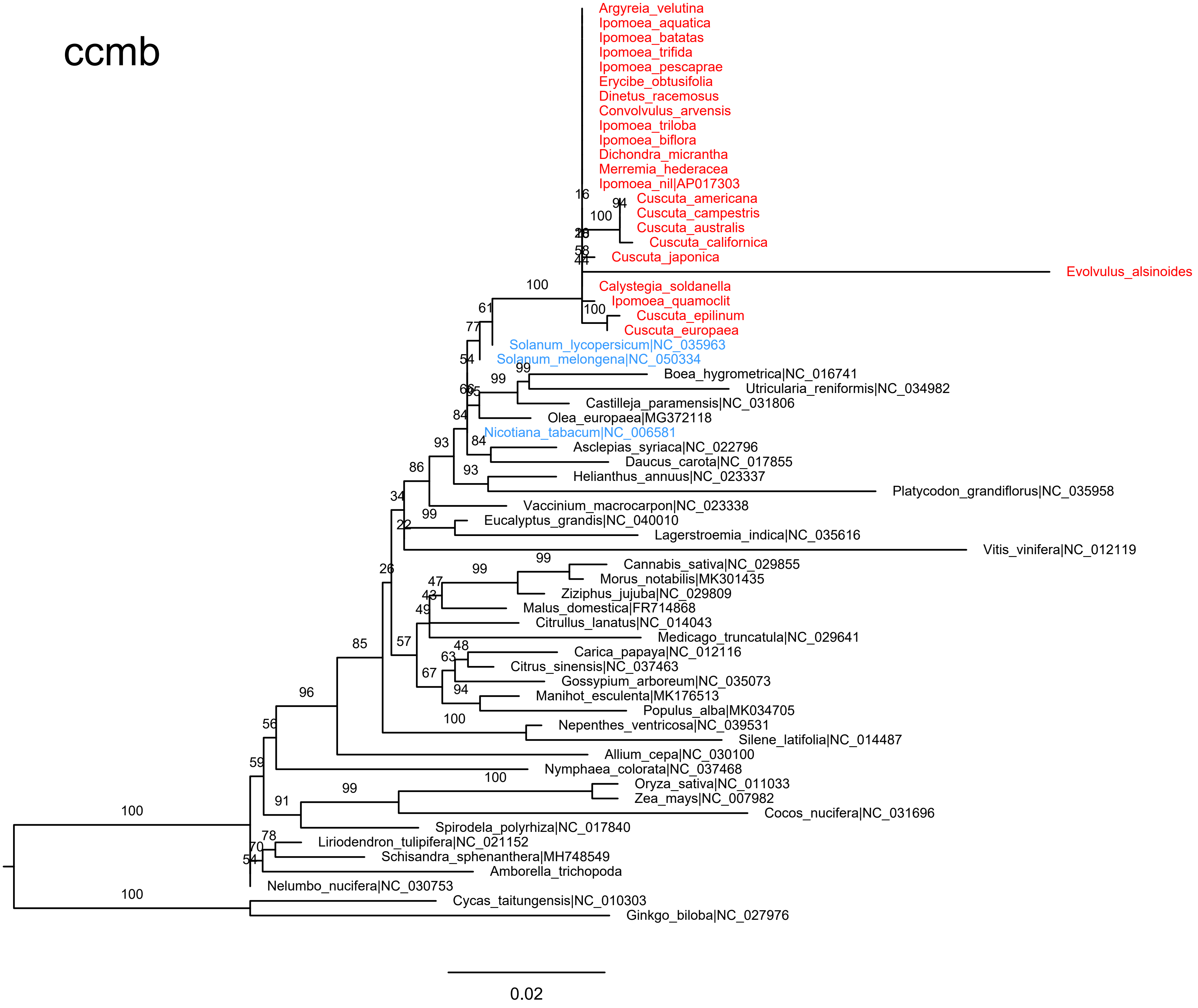
ccmb
Argyreia_velutina
Ipomoea_aquatica
Ipomoea_batatas
Ipomoea_trifida
Ipomoea_pescaprae
Erycibe_obtusifolia
Dinetus_racemosus
Convolvulus_arvensis
Ipomoea_triloba
Ipomoea_biflora
Dichondra_micrantha
Merremia_hederacea
Ipomoea_nil|AP017303
Cuscuta_americana
Cuscuta_campestris
Cuscuta_australis
Cuscuta_californica
Cuscuta_japonica
Evolvulus_alsinoides
Calystegia_soldanella
Ipomoea_quamoclit
Cuscuta_epilinum
Cuscuta_europaea
Solanum_lycopersicum|NC_035963
Solanum_melongena|NC_050334
Boea_hygrometrica|NC_016741
Utricularia_reniformis|NC_034982
Castilleja_paramensis|NC_031806
Olea_europaea|MG372118
Nicotiana_tabacum|NC_006581
Asclepias_syriaca|NC_022796
Daucus_carota|NC_017855
Helianthus_annuus|NC_023337
Platycodon_grandiflorus|NC_035958
Vaccinium_macrocarpon|NC_023338
Eucalyptus_grandis|NC_040010
Lagerstroemia_indica|NC_035616
Vitis_vinifera|NC_012119
Cannabis_sativa|NC_029855
Morus_notabilis|MK301435
Ziziphus_jujuba|NC_029809
Malus_domestica|FR714868
Citrullus_lanatus|NC_014043
Medicago_truncatula|NC_029641
Carica_papaya|NC_012116
Citrus_sinensis|NC_037463
Gossypium_arboreum|NC_035073
Manihot_esculenta|MK176513
Populus_alba|MK034705
Nepenthes_ventricosa|NC_039531
Silene_latifolia|NC_014487
Allium_cepa|NC_030100
Nymphaea_colorata|NC_037468
Oryza_sativa|NC_011033
Zea_mays|NC_007982
Cocos_nucifera|NC_031696
Spirodela_polyrhiza|NC_017840
Liriodendron_tulipifera|NC_021152
Schisandra_sphenanthera|MH748549
Amborella_trichopoda
Nelumbo_nucifera|NC_030753
Cycas_taitungensis|NC_010303
Ginkgo_biloba|NC_027976
0.02

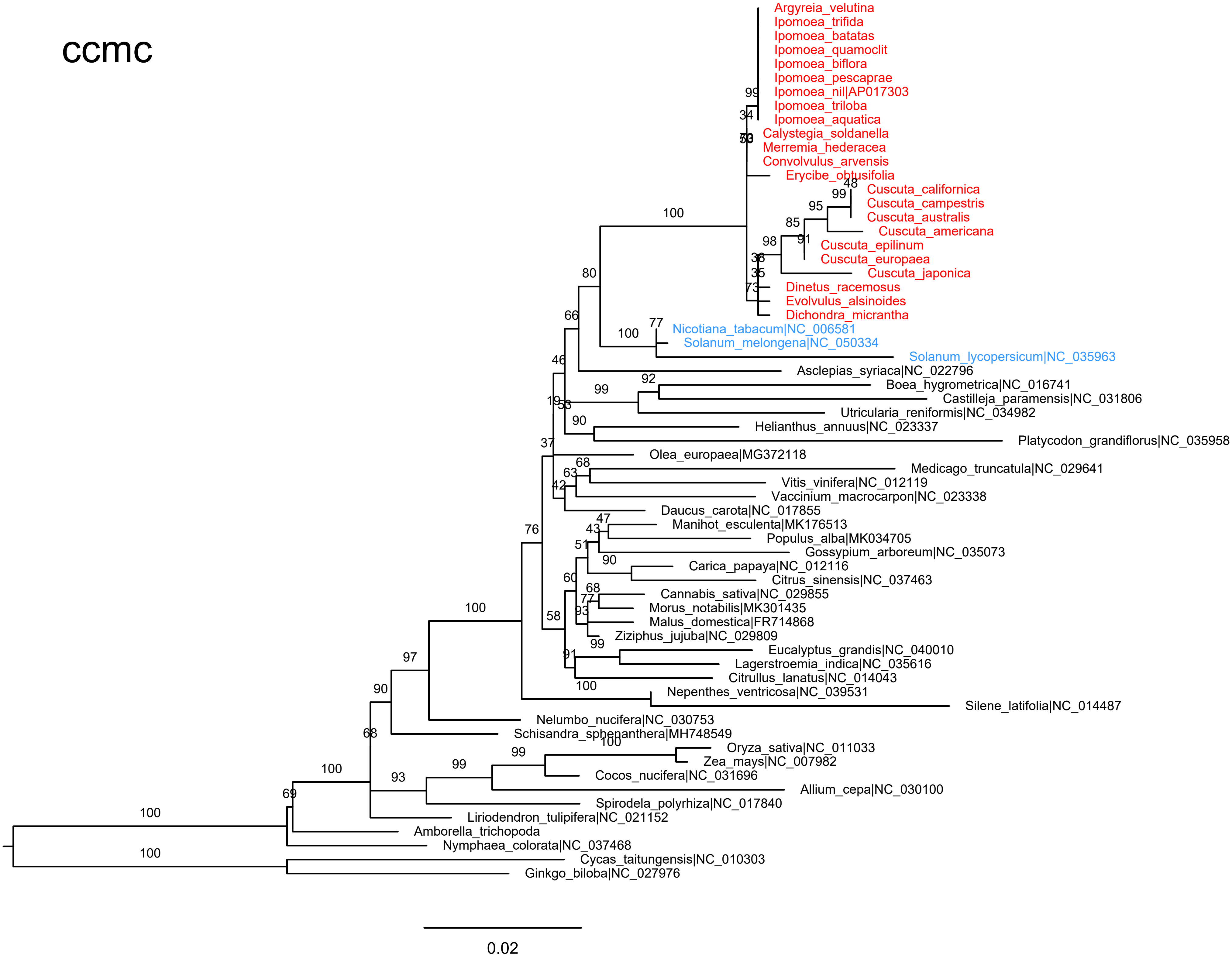
ccmc
Argyreia_velutina
Ipomoea_trifida
Ipomoea_batatas
Ipomoea_quamoclit
Ipomoea_biflora
Ipomoea_pescaprae
Ipomoea_nil|AP017303
Ipomoea_triloba
Ipomoea_aquatica
Calystegia_soldanella
Merremia_hederacea
Convolvulus_arvensis
Erycibe_obtusifolia
Cuscuta_californica
Cuscuta_campestris
Cuscuta_australis
Cuscuta_americana
Cuscuta_epilinum
Cuscuta_europaea
Cuscuta_japonica
Dinetus_racemosus
Evolvulus_alsinoides
Dichondra_micrantha
Nicotiana_tabacum|NC_006581
Solanum_melongena|NC_050334
Solanum_lycopersicum|NC_035963
Asclepias_syriaca|NC_022796
Boea_hygrometrica|NC_016741
Castilleja_paramensis|NC_031806
Utricularia_reniformis|NC_034982
Helianthus_annuus|NC_023337
Platycodon_grandiflorus|NC_035958
Olea_europaea|MG372118
Medicago_truncatula|NC_029641
Vitis_vinifera|NC_012119
Vaccinium_macrocarpon|NC_023338
Daucus_carota|NC_017855
Manihot_esculenta|MK176513
Populus_alba|MK034705
Gossypium_arboreum|NC_035073
Carica_papaya|NC_012116
Citrus_sinensis|NC_037463
Cannabis_sativa|NC_029855
Morus_notabilis|MK301435
Malus_domestica|FR714868
Ziziphus_jujuba|NC_029809
Eucalyptus_grandis|NC_040010
Lagerstroemia_indica|NC_035616
Citrullus_lanatus|NC_014043
Nepenthes_ventricosa|NC_039531
Silene_latifolia|NC_014487
Nelumbo_nucifera|NC_030753
Schisandra_sphenanthera|MH748549
Oryza_sativa|NC_011033
Zea_mays|NC_007982
Cocos_nucifera|NC_031696
Allium_cepa|NC_030100
Spirodela_polyrhiza|NC_017840
Liriodendron_tulipifera|NC_021152
Amborella_trichopoda
Nymphaea_colorata|NC_037468
Cycas_taitungensis|NC_010303
Ginkgo_biloba|NC_027976
0.02

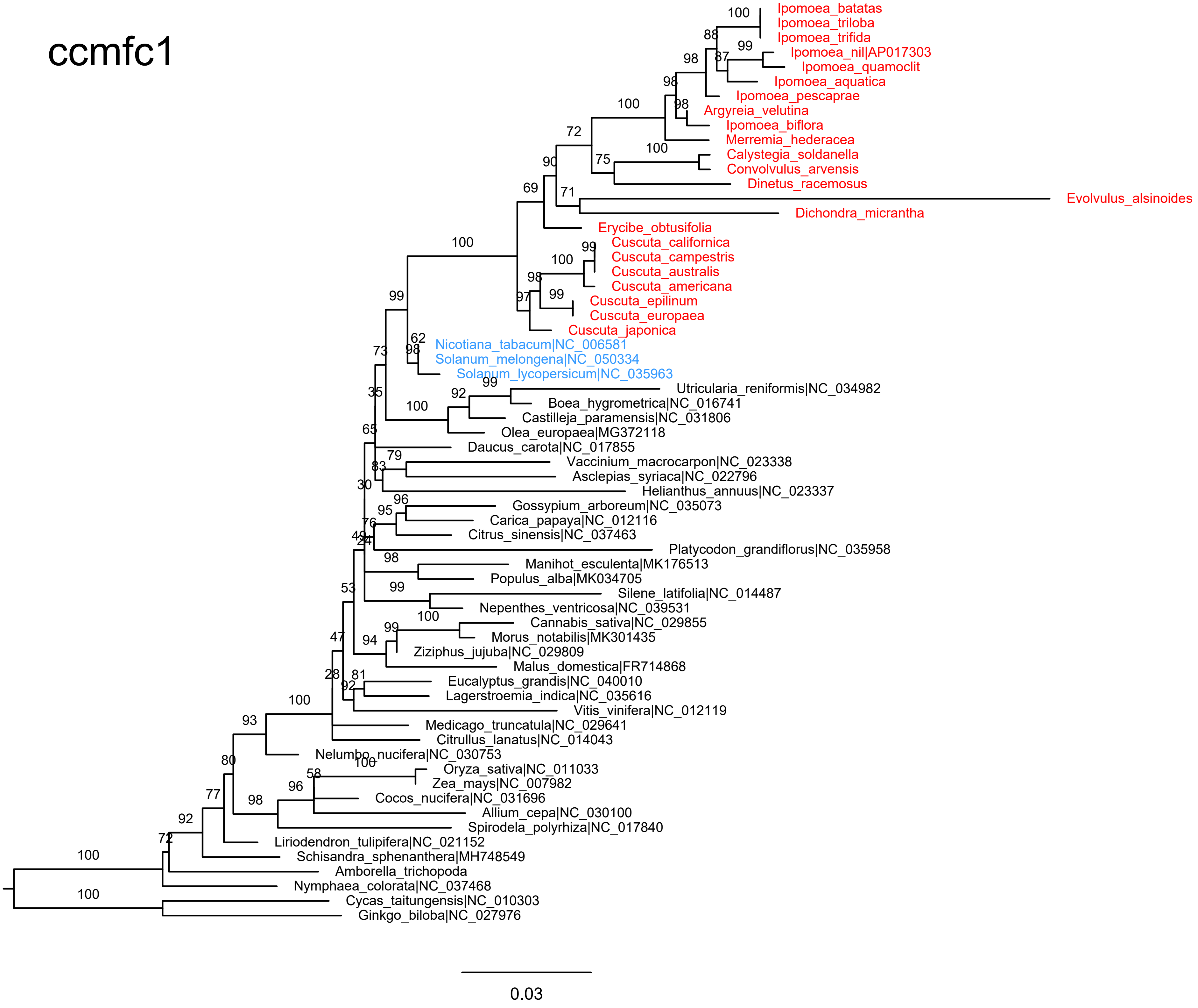

ccmfc1
Ipomoea_batatas
Ipomoea_triloba
Ipomoea_trifida
Ipomoea_nil|AP017303
Ipomoea_quamoclit
Ipomoea_aquatica
Ipomoea_pescaprae
Argyreia_velutina
Ipomoea_biflora
Merremia_hederacea
Calystegia_soldanella
Convolvulus_arvensis
Dinetus_racemosus
Evolvulus_alsinoides
Dichondra_micrantha
Erycibe_obtusifolia
Cuscuta_californica
Cuscuta_campestris
Cuscuta_australis
Cuscuta_americana
Cuscuta_epilinum
Cuscuta_europaea
Cuscuta_japonica
Nicotiana_tabacum|NC_006581
Solanum_melongena|NC_050334
Solanum_lycopersicum|NC_035963
Utricularia_reniformis|NC_034982
Boea_hygrometrica|NC_016741
Castilleja_paramensis|NC_031806
Olea_europaea|MG372118
Daucus_carota|NC_017855
Vaccinium_macrocarpon|NC_023338
Asclepias_syriaca|NC_022796
Helianthus_annuus|NC_023337
Gossypium_arboreum|NC_035073
Carica_papaya|NC_012116
Citrus_sinensis|NC_037463
Platycodon_grandiflorus|NC_035958
Manihot_esculenta|MK176513
Populus_alba|MK034705
Silene_latifolia|NC_014487
Nepenthes_ventricosa|NC_039531
Cannabis_sativa|NC_029855
Morus_notabilis|MK301435
Ziziphus_jujuba|NC_029809
Malus_domestica|FR714868
Eucalyptus_grandis|NC_040010
Lagerstroemia_indica|NC_035616
Vitis_vinifera|NC_012119
Medicago_truncatula|NC_029641
Citrullus_lanatus|NC_014043
Nelumbo_nucifera|NC_030753
Oryza_sativa|NC_011033
Zea_mays|NC_007982
Cocos_nucifera|NC_031696
Allium_cepa|NC_030100
Spirodela_polyrhiza|NC_017840
Liriodendron_tulipifera|NC_021152
Schisandra_sphenanthera|MH748549
Amborella_trichopoda
Nymphaea_colorata|NC_037468
Cycas_taitungensis|NC_010303
Ginkgo_biloba|NC_027976
0.03

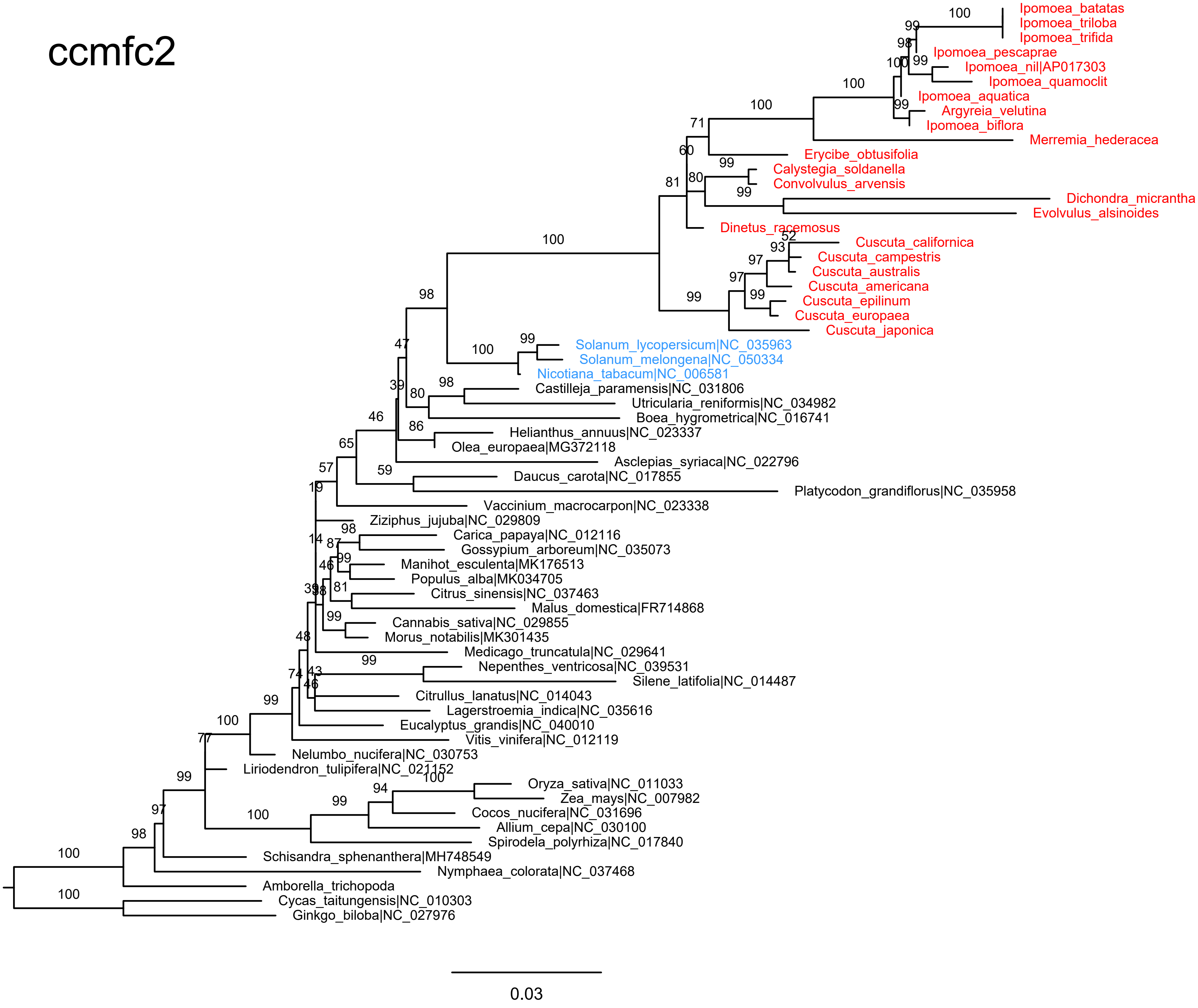
ccmfc2
Ipomoea_batatas
Ipomoea_triloba
Ipomoea_trifida
Ipomoea_pescaprae
Ipomoea_nil|AP017303
Ipomoea_quamoclit
Ipomoea_aquatica
Argyreia_velutina
Ipomoea_biflora
Merremia_hederacea
Erycibe_obtusifolia
Calystegia_soldanella
Convolvulus_arvensis
Dichondra_micrantha
Evolvulus_alsinoides
Dinetus_racemosus
Cuscuta_californica
Cuscuta_campestris
Cuscuta_australis
Cuscuta_americana
Cuscuta_epilinum
Cuscuta_europaea
Cuscuta_japonica
Solanum_lycopersicum|NC_035963
Solanum_melongena|NC_050334
Nicotiana_tabacum|NC_006581
Castilleja_paramensis|NC_031806
Utricularia_reniformis|NC_034982
Boea_hygrometrica|NC_016741
Helianthus_annuus|NC_023337
Olea_europaea|MG372118
Asclepias_syriaca|NC_022796
Daucus_carota|NC_017855
Platycodon_grandiflorus|NC_035958
Vaccinium_macrocarpon|NC_023338
Ziziphus_jujuba|NC_029809
Carica_papaya|NC_012116
Gossypium_arboreum|NC_035073
Manihot_esculenta|MK176513
Populus_alba|MK034705
Citrus_sinensis|NC_037463
Malus_domestica|FR714868
Cannabis_sativa|NC_029855
Morus_notabilis|MK301435
Medicago_truncatula|NC_029641
Nepenthes_ventricosa|NC_039531
Silene_latifolia|NC_014487
Citrullus_lanatus|NC_014043
Lagerstroemia_indica|NC_035616
Eucalyptus_grandis|NC_040010
Vitis_vinifera|NC_012119
Nelumbo_nucifera|NC_030753
Liriodendron_tulipifera|NC_021152
Oryza_sativa|NC_011033
Zea_mays|NC_007982
Cocos_nucifera|NC_031696
Allium_cepa|NC_030100
Spirodela_polyrhiza|NC_017840
Schisandra_sphenanthera|MH748549
Nymphaea_colorata|NC_037468
Amborella_trichopoda
Cycas_taitungensis|NC_010303
Ginkgo_biloba|NC_027976
0.03

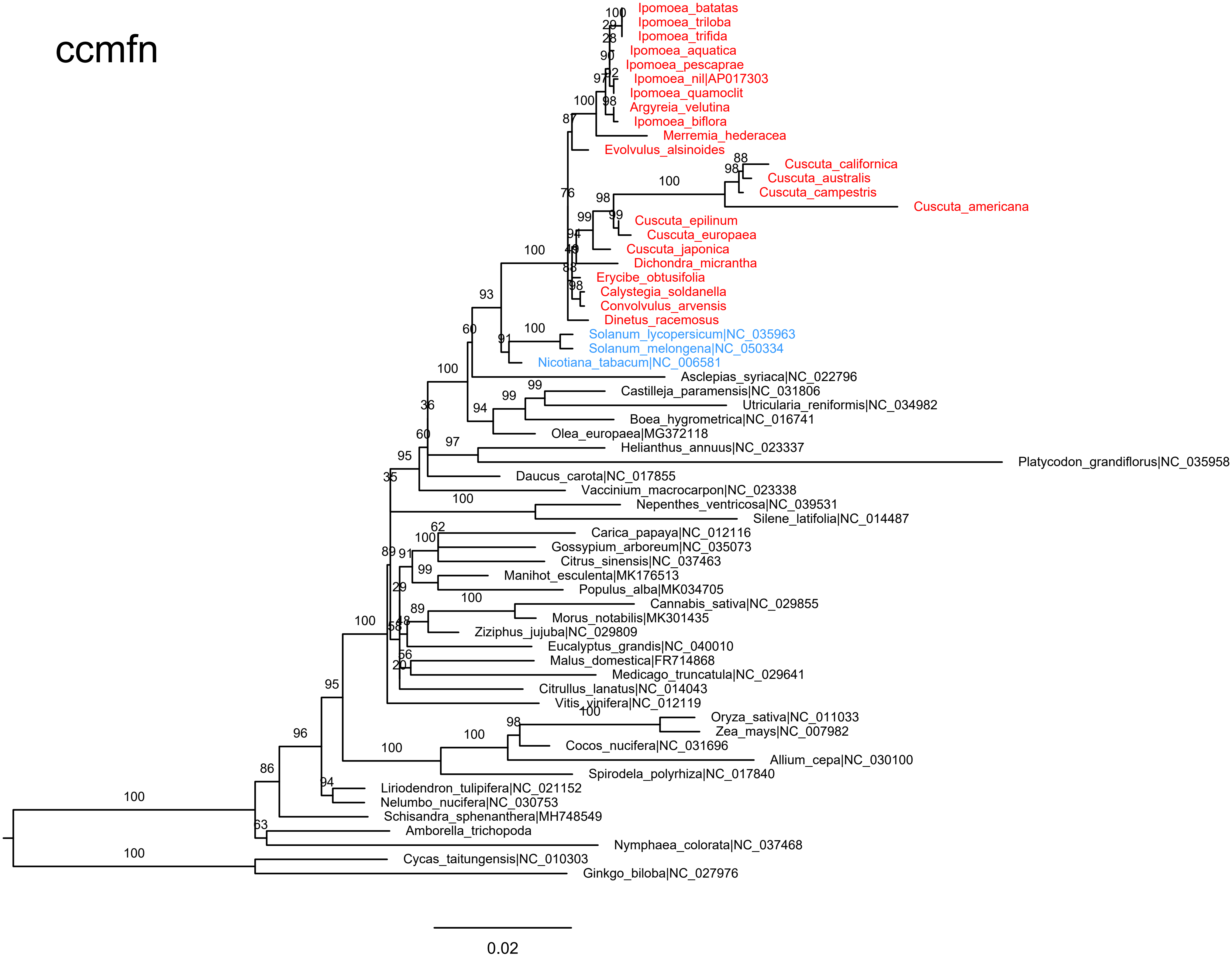
ccmfn
Ipomoea_batatas
Ipomoea_triloba
Ipomoea_trifida
Ipomoea_aquatica
Ipomoea_pescaprae
Ipomoea_nil|AP017303
Ipomoea_quamoclit
Argyreia_velutina
Ipomoea_biflora
Merremia_hederacea
Evolvulus_alsinoides
Cuscuta_californica
Cuscuta_australis
Cuscuta_campestris
Cuscuta_americana
Cuscuta_epilinum
Cuscuta_europaea
Cuscuta_japonica
Dichondra_micrantha
Erycibe_obtusifolia
Calystegia_soldanella
Convolvulus_arvensis
Dinetus_racemosus
Solanum_lycopersicum|NC_035963
Solanum_melongena|NC_050334
Nicotiana_tabacum|NC_006581
Asclepias_syriaca|NC_022796
Castilleja_paramensis|NC_031806
Utricularia_reniformis|NC_034982
Boea_hygrometrica|NC_016741
Olea_europaea|MG372118
Helianthus_annuus|NC_023337
Platycodon_grandiflorus|NC_035958
Daucus_carota|NC_017855
Vaccinium_macrocarpon|NC_023338
Nepenthes_ventricosa|NC_039531
Silene_latifolia|NC_014487
Carica_papaya|NC_012116
Gossypium_arboreum|NC_035073
Citrus_sinensis|NC_037463
Manihot_esculenta|MK176513
Populus_alba|MK034705
Cannabis_sativa|NC_029855
Morus_notabilis|MK301435
Ziziphus_jujuba|NC_029809
Eucalyptus_grandis|NC_040010
Malus_domestica|FR714868
Medicago_truncatula|NC_029641
Citrullus_lanatus|NC_014043
Vitis_vinifera|NC_012119
Oryza_sativa|NC_011033
Zea_mays|NC_007982
Cocos_nucifera|NC_031696
Allium_cepa|NC_030100
Spirodela_polyrhiza|NC_017840
Liriodendron_tulipifera|NC_021152
Nelumbo_nucifera|NC_030753
Schisandra_sphenanthera|MH748549
Amborella_trichopoda
Nymphaea_colorata|NC_037468
Cycas_taitungensis|NC_010303
Ginkgo_biloba|NC_027976
0.02

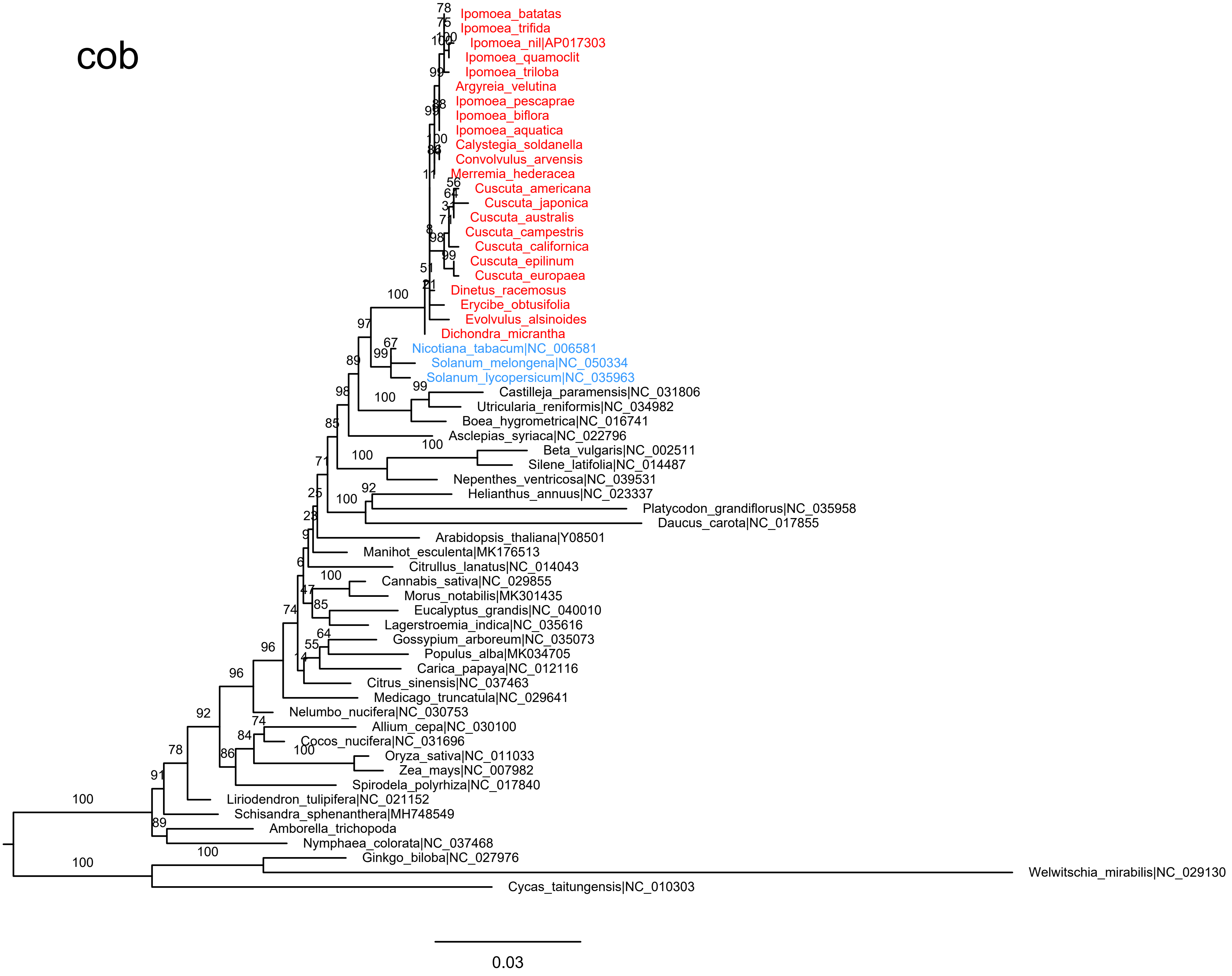
cob
Ipomoea_batatas
Ipomoea_trifida
Ipomoea_nil|AP017303
Ipomoea_quamoclit
Ipomoea_triloba
Argyreia_velutina
Ipomoea_pescaprae
Ipomoea_biflora
Ipomoea_aquatica
Calystegia_soldanella
Convolvulus_arvensis
Merremia_hederacea
Cuscuta_americana
Cuscuta_japonica
Cuscuta_australis
Cuscuta_campestris
Cuscuta_californica
Cuscuta_epilinum
Cuscuta_europaea
Dinetus_racemosus
Erycibe_obtusifolia
Evolvulus_alsinoides
Dichondra_micrantha
Nicotiana_tabacum|NC_006581
Solanum_melongena|NC_050334
Solanum_lycopersicum|NC_035963
Castilleja_paramensis|NC_031806
Utricularia_reniformis|NC_034982
Boea_hygrometrica|NC_016741
Asclepias_syriaca|NC_022796
Beta_vulgaris|NC_002511
Silene_latifolia|NC_014487
Nepenthes_ventricosa|NC_039531
Helianthus_annuus|NC_023337
Platycodon_grandiflorus|NC_035958
Daucus_carota|NC_017855
Arabidopsis_thaliana|Y08501
Manihot_esculenta|MK176513
Citrullus_lanatus|NC_014043
Cannabis_sativa|NC_029855
Morus_notabilis|MK301435
Eucalyptus_grandis|NC_040010
Lagerstroemia_indica|NC_035616
Gossypium_arboreum|NC_035073
Populus_alba|MK034705
Carica_papaya|NC_012116
Citrus_sinensis|NC_037463
Medicago_truncatula|NC_029641
Nelumbo_nucifera|NC_030753
Allium_cepa|NC_030100
Cocos_nucifera|NC_031696
Oryza_sativa|NC_011033
Zea_mays|NC_007982
Spirodela_polyrhiza|NC_017840
Liriodendron_tulipifera|NC_021152
Schisandra_sphenanthera|MH748549
Amborella_trichopoda
Nymphaea_colorata|NC_037468
Ginkgo_biloba|NC_027976
Welwitschia_mirabilis|NC_029130
Cycas_taitungensis|NC_010303
0.03

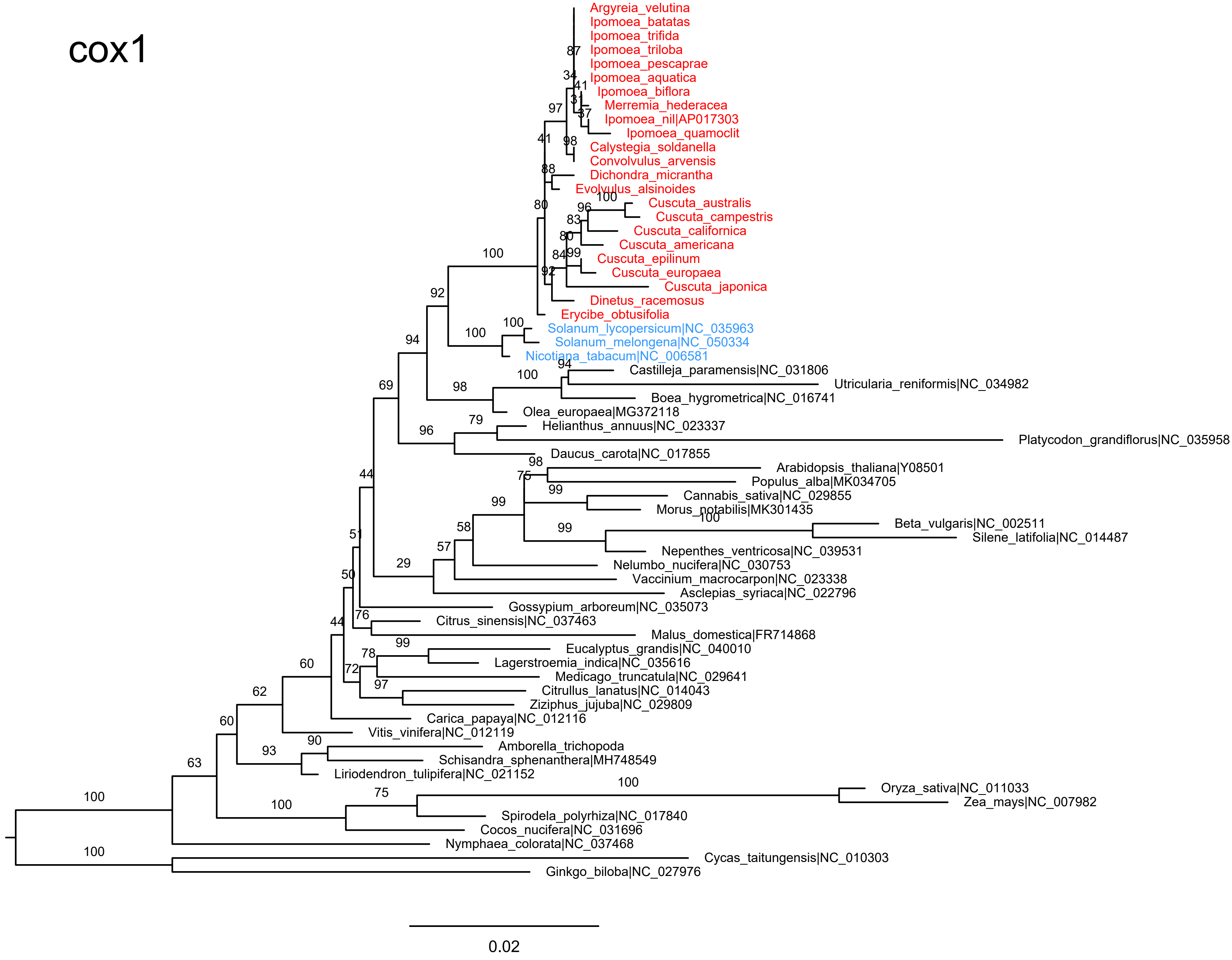

cox1
Argyreia_velutina
Ipomoea_batatas
Ipomoea_trifida
Ipomoea_triloba
Ipomoea_pescaprae
Ipomoea_aquatica
Ipomoea_biflora
Merremia_hederacea
Ipomoea_nil|AP017303
Ipomoea_quamoclit
Calystegia_soldanella
Convolvulus_arvensis
Dichondra_micrantha
Evolvulus_alsinoides
Cuscuta_australis
Cuscuta_campestris
Cuscuta_californica
Cuscuta_americana
Cuscuta_epilinum
Cuscuta_europaea
Cuscuta_japonica
Dinetus_racemosus
Erycibe_obtusifolia
Solanum_lycopersicum|NC_035963
Solanum_melongena|NC_050334
Nicotiana_tabacum|NC_006581
Castilleja_paramensis|NC_031806
Utricularia_reniformis|NC_034982
Boea_hygrometrica|NC_016741
Olea_europaea|MG372118
Helianthus_annuus|NC_023337
Platycodon_grandiflorus|NC_035958
Daucus_carota|NC_017855
Arabidopsis_thaliana|Y08501
Populus_alba|MK034705
Cannabis_sativa|NC_029855
Morus_notabilis|MK301435
Beta_vulgaris|NC_002511
Silene_latifolia|NC_014487
Nepenthes_ventricosa|NC_039531
Nelumbo_nucifera|NC_030753
Vaccinium_macrocarpon|NC_023338
Asclepias_syriaca|NC_022796
Gossypium_arboreum|NC_035073
Citrus_sinensis|NC_037463
Malus_domestica|FR714868
Eucalyptus_grandis|NC_040010
Lagerstroemia_indica|NC_035616
Medicago_truncatula|NC_029641
Citrullus_lanatus|NC_014043
Ziziphus_jujuba|NC_029809
Carica_papaya|NC_012116
Vitis_vinifera|NC_012119
Amborella_trichopoda
Schisandra_sphenanthera|MH748549
Liriodendron_tulipifera|NC_021152
Oryza_sativa|NC_011033
Zea_mays|NC_007982
Spirodela_polyrhiza|NC_017840
Cocos_nucifera|NC_031696
Nymphaea_colorata|NC_037468
Cycas_taitungensis|NC_010303
Ginkgo_biloba|NC_027976
0.02

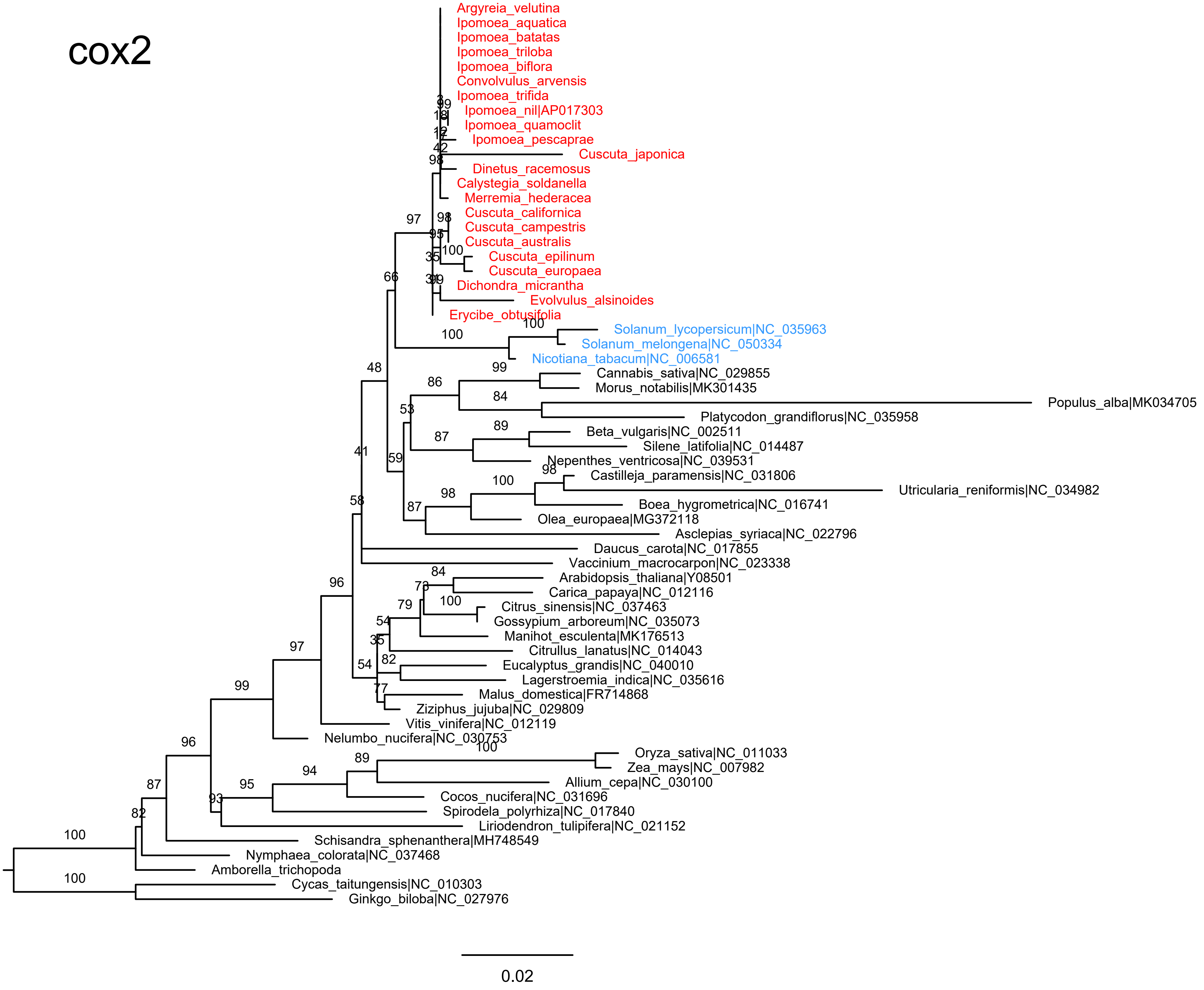
cox2
Argyreia_velutina
Ipomoea_aquatica
Ipomoea_batatas
Ipomoea_triloba
Ipomoea_biflora
Convolvulus_arvensis
Ipomoea_trifida
Ipomoea_nil|AP017303
Ipomoea_quamoclit
Ipomoea_pescaprae
Cuscuta_japonica
Dinetus_racemosus
Calystegia_soldanella
Merremia_hederacea
Cuscuta_californica
Cuscuta_campestris
Cuscuta_australis
Cuscuta_epilinum
Cuscuta_europaea
Dichondra_micrantha
Evolvulus_alsinoides
Erycibe_obtusifolia
Solanum_lycopersicum|NC_035963
Solanum_melongena|NC_050334
Nicotiana_tabacum|NC_006581
Cannabis_sativa|NC_029855
Morus_notabilis|MK301435
Populus_alba|MK034705
Platycodon_grandiflorus|NC_035958
Beta_vulgaris|NC_002511
Silene_latifolia|NC_014487
Nepenthes_ventricosa|NC_039531
Castilleja_paramensis|NC_031806
Utricularia_reniformis|NC_034982
Boea_hygrometrica|NC_016741
Olea_europaea|MG372118
Asclepias_syriaca|NC_022796
Daucus_carota|NC_017855
Vaccinium_macrocarpon|NC_023338
Arabidopsis_thaliana|Y08501
Carica_papaya|NC_012116
Citrus_sinensis|NC_037463
Gossypium_arboreum|NC_035073
Manihot_esculenta|MK176513
Citrullus_lanatus|NC_014043
Eucalyptus_grandis|NC_040010
Lagerstroemia_indica|NC_035616
Malus_domestica|FR714868
Ziziphus_jujuba|NC_029809
Vitis_vinifera|NC_012119
Nelumbo_nucifera|NC_030753
Oryza_sativa|NC_011033
Zea_mays|NC_007982
Allium_cepa|NC_030100
Cocos_nucifera|NC_031696
Spirodela_polyrhiza|NC_017840
Liriodendron_tulipifera|NC_021152
Schisandra_sphenanthera|MH748549
Nymphaea_colorata|NC_037468
Amborella_trichopoda
Cycas_taitungensis|NC_010303
Ginkgo_biloba|NC_027976
0.02

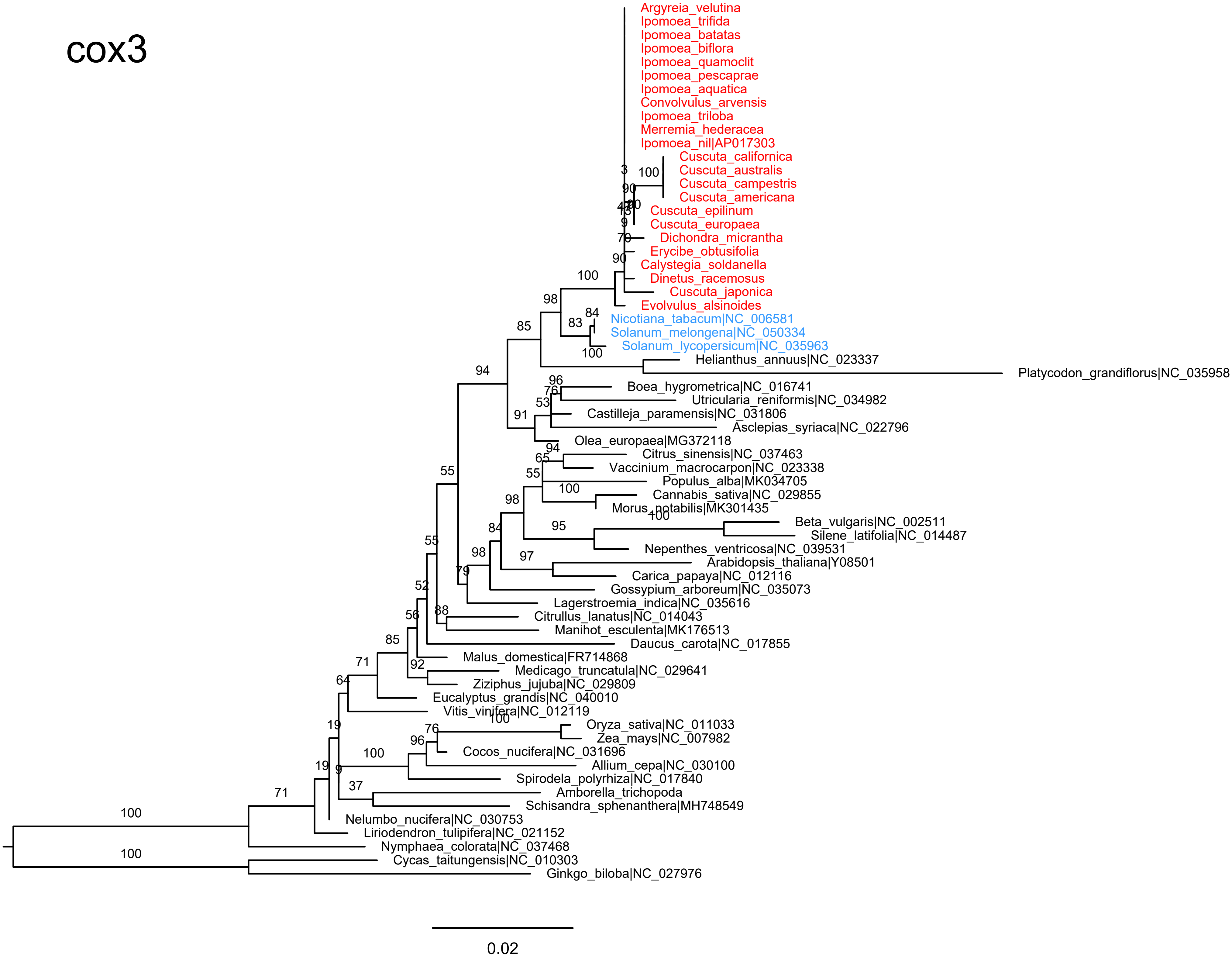
cox3
Argyreia_velutina
Ipomoea_trifida
Ipomoea_batatas
Ipomoea_biflora
Ipomoea_quamoclit
Ipomoea_pescaprae
Ipomoea_aquatica
Convolvulus_arvensis
Ipomoea_triloba
Merremia_hederacea
Ipomoea_nil|AP017303
Cuscuta_californica
Cuscuta_australis
Cuscuta_campestris
Cuscuta_americana
Cuscuta_epilinum
Cuscuta_europaea
Dichondra_micrantha
Erycibe_obtusifolia
Calystegia_soldanella
Dinetus_racemosus
Cuscuta_japonica
Evolvulus_alsinoides
Nicotiana_tabacum|NC_006581
Solanum_melongena|NC_050334
Solanum_lycopersicum|NC_035963
Helianthus_annuus|NC_023337
Platycodon_grandiflorus|NC_035958
Boea_hygrometrica|NC_016741
Utricularia_reniformis|NC_034982
Castilleja_paramensis|NC_031806
Asclepias_syriaca|NC_022796
Olea_europaea|MG372118
Citrus_sinensis|NC_037463
Vaccinium_macrocarpon|NC_023338
Populus_alba|MK034705
Cannabis_sativa|NC_029855
Morus_notabilis|MK301435
Beta_vulgaris|NC_002511
Silene_latifolia|NC_014487
Nepenthes_ventricosa|NC_039531
Arabidopsis_thaliana|Y08501
Carica_papaya|NC_012116
Gossypium_arboreum|NC_035073
Lagerstroemia_indica|NC_035616
Citrullus_lanatus|NC_014043
Manihot_esculenta|MK176513
Daucus_carota|NC_017855
Malus_domestica|FR714868
Medicago_truncatula|NC_029641
Ziziphus_jujuba|NC_029809
Eucalyptus_grandis|NC_040010
Vitis_vinifera|NC_012119
Oryza_sativa|NC_011033
Zea_mays|NC_007982
Cocos_nucifera|NC_031696
Allium_cepa|NC_030100
Spirodela_polyrhiza|NC_017840
Amborella_trichopoda
Schisandra_sphenanthera|MH748549
Nelumbo_nucifera|NC_030753
Liriodendron_tulipifera|NC_021152
Nymphaea_colorata|NC_037468
Cycas_taitungensis|NC_010303
Ginkgo_biloba|NC_027976
0.02

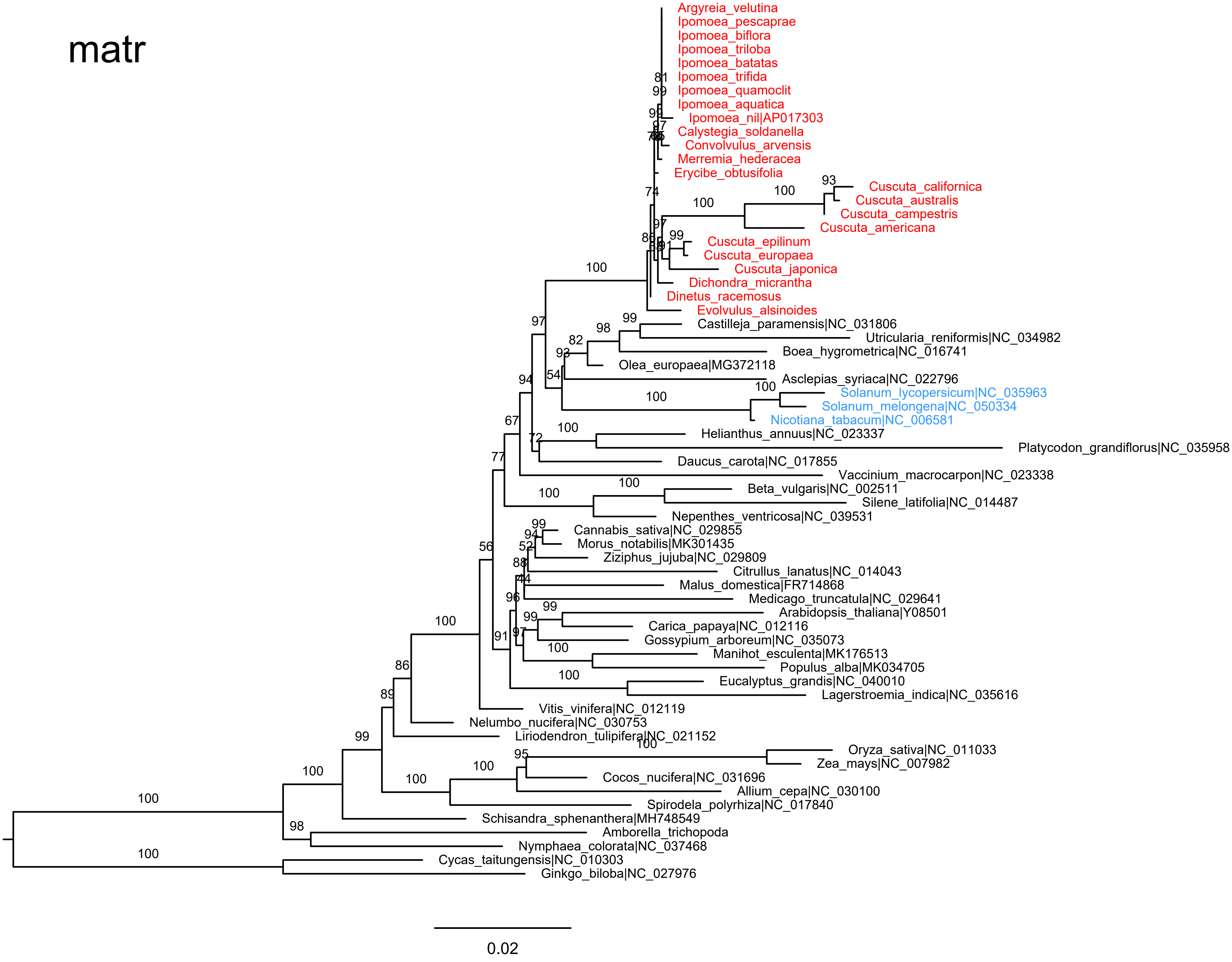
matr
Argyreia_velutina
Ipomoea_pescaprae
Ipomoea_biflora
Ipomoea_triloba
Ipomoea_batatas
Ipomoea_trifida
Ipomoea_quamoclit
Ipomoea_aquatica
Ipomoea_nil|AP017303
Calystegia_soldanella
Convolvulus_arvensis
Merremia_hederacea
Erycibe_obtusifolia
Cuscuta_californica
Cuscuta_australis
Cuscuta_campestris
Cuscuta_americana
Cuscuta_epilinum
Cuscuta_europaea
Cuscuta_japonica
Dichondra_micrantha
Dinetus_racemosus
Evolvulus_alsinoides
Castilleja_paramensis|NC_031806
Utricularia_reniformis|NC_034982
Boea_hygrometrica|NC_016741
Olea_europaea|MG372118
Asclepias_syriaca|NC_022796
Solanum_lycopersicum|NC_035963
Solanum_melongena|NC_050334
Nicotiana_tabacum|NC_006581
Helianthus_annuus|NC_023337
Platycodon_grandiflorus|NC_035958
Daucus_carota|NC_017855
Vaccinium_macrocarpon|NC_023338
Beta_vulgaris|NC_002511
Silene_latifolia|NC_014487
Nepenthes_ventricosa|NC_039531
Cannabis_sativa|NC_029855
Morus_notabilis|MK301435
Ziziphus_jujuba|NC_029809
Citrullus_lanatus|NC_014043
Malus_domestica|FR714868
Medicago_truncatula|NC_029641
Arabidopsis_thaliana|Y08501
Carica_papaya|NC_012116
Gossypium_arboreum|NC_035073
Manihot_esculenta|MK176513
Populus_alba|MK034705
Eucalyptus_grandis|NC_040010
Lagerstroemia_indica|NC_035616
Vitis_vinifera|NC_012119
Nelumbo_nucifera|NC_030753
Liriodendron_tulipifera|NC_021152
Oryza_sativa|NC_011033
Zea_mays|NC_007982
Cocos_nucifera|NC_031696
Allium_cepa|NC_030100
Spirodela_polyrhiza|NC_017840
Schisandra_sphenanthera|MH748549
Amborella_trichopoda
Nymphaea_colorata|NC_037468
Cycas_taitungensis|NC_010303
Ginkgo_biloba|NC_027976
0.02

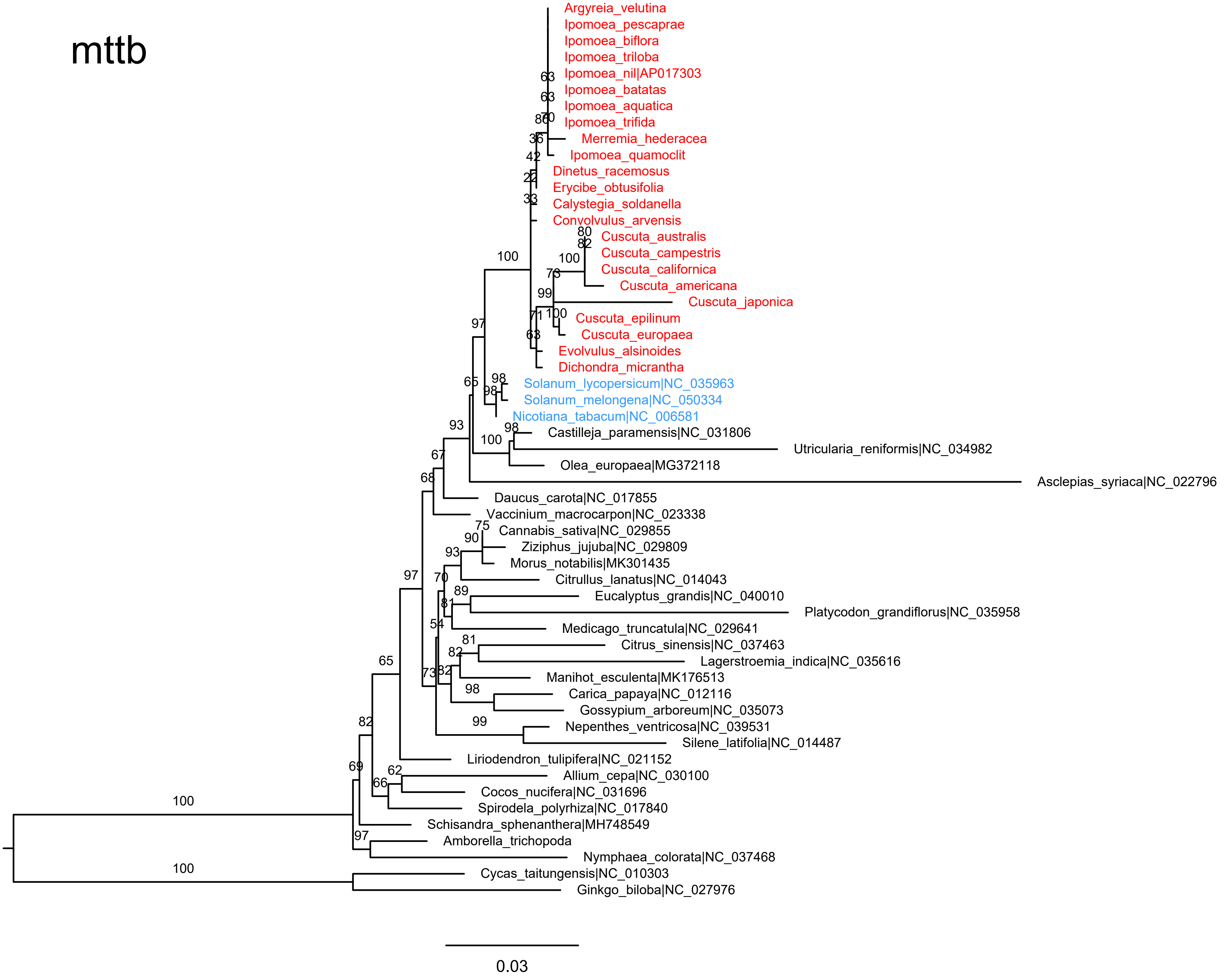
mttb
Argyreia_velutina
Ipomoea_pescaprae
Ipomoea_biflora
Ipomoea_triloba
Ipomoea_nil|AP017303
Ipomoea_batatas
Ipomoea_aquatica
Ipomoea_trifida
Merremia_hederacea
Ipomoea_quamoclit
Dinetus_racemosus
Erycibe_obtusifolia
Calystegia_soldanella
Convolvulus_arvensis
Cuscuta_australis
Cuscuta_campestris
Cuscuta_californica
Cuscuta_americana
Cuscuta_japonica
Cuscuta_epilinum
Cuscuta_europaea
Evolvulus_alsinoides
Dichondra_micrantha
Solanum_lycopersicum|NC_035963
Solanum_melongena|NC_050334
Nicotiana_tabacum|NC_006581
Castilleja_paramensis|NC_031806
Utricularia_reniformis|NC_034982
Olea_europaea|MG372118
Asclepias_syriaca|NC_022796
Daucus_carota|NC_017855
Vaccinium_macrocarpon|NC_023338
Cannabis_sativa|NC_029855
Ziziphus_jujuba|NC_029809
Morus_notabilis|MK301435
Citrullus_lanatus|NC_014043
Eucalyptus_grandis|NC_040010
Platycodon_grandiflorus|NC_035958
Medicago_truncatula|NC_029641
Citrus_sinensis|NC_037463
Lagerstroemia_indica|NC_035616
Manihot_esculenta|MK176513
Carica_papaya|NC_012116
Gossypium_arboreum|NC_035073
Nepenthes_ventricosa|NC_039531
Silene_latifolia|NC_014487
Liriodendron_tulipifera|NC_021152
Allium_cepa|NC_030100
Cocos_nucifera|NC_031696
Spirodela_polyrhiza|NC_017840
Schisandra_sphenanthera|MH748549
Amborella_trichopoda
Nymphaea_colorata|NC_037468
Cycas_taitungensis|NC_010303
Ginkgo_biloba|NC_027976
0.03

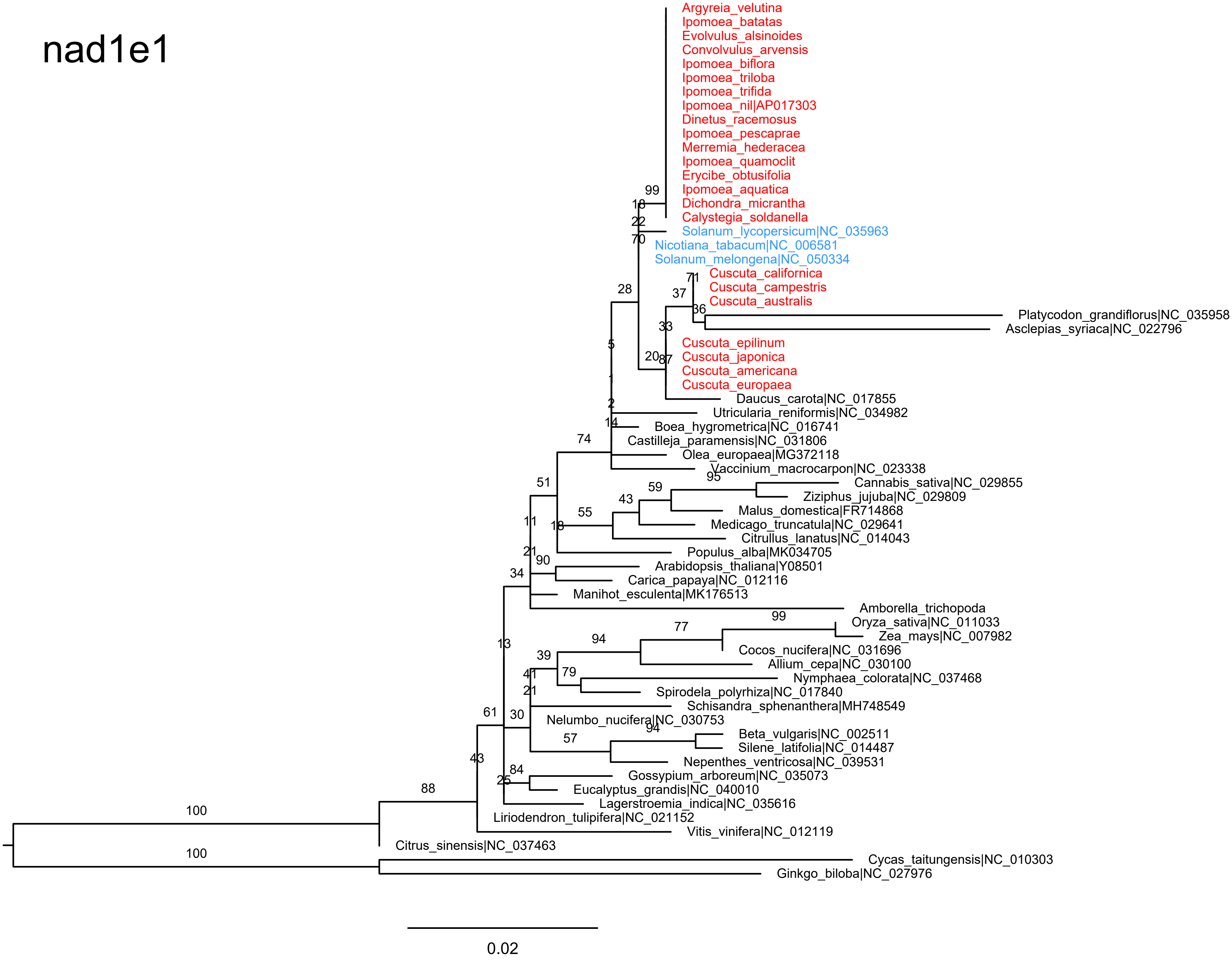

nad1e1
Argyreia_velutina
Ipomoea_batatas
Evolvulus_alsinoides
Convolvulus_arvensis
Ipomoea_biflora
Ipomoea_triloba
Ipomoea_trifida
Ipomoea_nil|AP017303
Dinetus_racemosus
Ipomoea_pescaprae
Merremia_hederacea
Ipomoea_quamoclit
Erycibe_obtusifolia
Ipomoea_aquatica
Dichondra_micrantha
Calystegia_soldanella
Solanum_lycopersicum|NC_035963
Nicotiana_tabacum|NC_006581
Solanum_melongena|NC_050334
Cuscuta_californica
Cuscuta_campestris
Cuscuta_australis
Platycodon_grandiflorus|NC_035958
Asclepias_syriaca|NC_022796
Cuscuta_epilinum
Cuscuta_japonica
Cuscuta_americana
Cuscuta_europaea
Daucus_carota|NC_017855
Utricularia_reniformis|NC_034982
Boea_hygrometrica|NC_016741
Castilleja_paramensis|NC_031806
Olea_europaea|MG372118
Vaccinium_macrocarpon|NC_023338
Cannabis_sativa|NC_029855
Ziziphus_jujuba|NC_029809
Malus_domestica|FR714868
Medicago_truncatula|NC_029641
Citrullus_lanatus|NC_014043
Populus_alba|MK034705
Arabidopsis_thaliana|Y08501
Carica_papaya|NC_012116
Manihot_esculenta|MK176513
Amborella_trichopoda
Oryza_sativa|NC_011033
Zea_mays|NC_007982
Cocos_nucifera|NC_031696
Allium_cepa|NC_030100
Nymphaea_colorata|NC_037468
Spirodela_polyrhiza|NC_017840
Schisandra_sphenanthera|MH748549
Nelumbo_nucifera|NC_030753
Beta_vulgaris|NC_002511
Silene_latifolia|NC_014487
Nepenthes_ventricosa|NC_039531
Gossypium_arboreum|NC_035073
Eucalyptus_grandis|NC_040010
Lagerstroemia_indica|NC_035616
Liriodendron_tulipifera|NC_021152
Vitis_vinifera|NC_012119
Citrus_sinensis|NC_037463
Cycas_taitungensis|NC_010303
Ginkgo_biloba|NC_027976
0.02

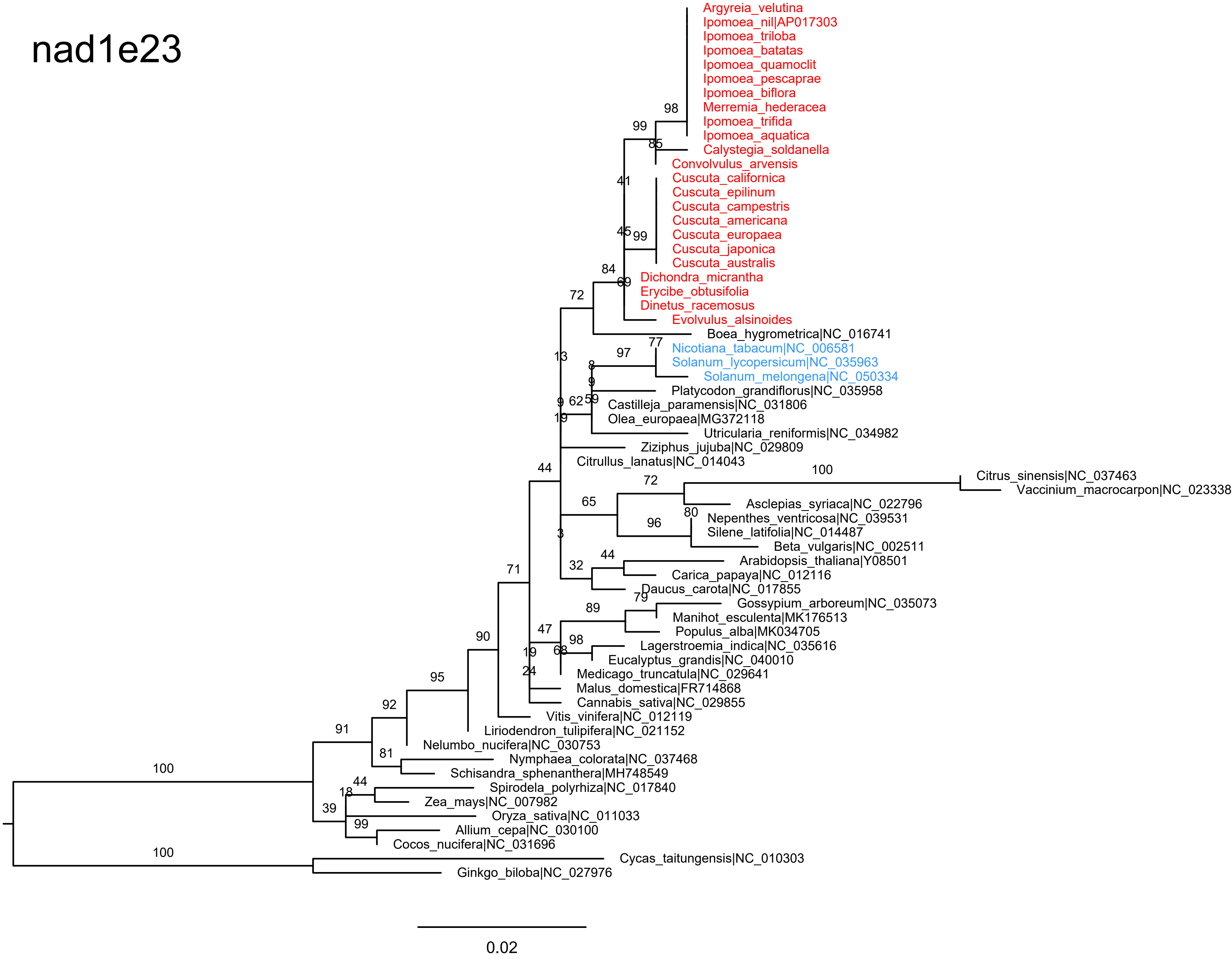
nad1e23
Argyreia_velutina
Ipomoea_nil|AP017303
Ipomoea_triloba
Ipomoea_batatas
Ipomoea_quamoclit
Ipomoea_pescaprae
Ipomoea_biflora
Merremia_hederacea
Ipomoea_trifida
Ipomoea_aquatica
Calystegia_soldanella
Convolvulus_arvensis
Cuscuta_californica
Cuscuta_epilinum
Cuscuta_campestris
Cuscuta_americana
Cuscuta_europaea
Cuscuta_japonica
Cuscuta_australis
Dichondra_micrantha
Erycibe_obtusifolia
Dinetus_racemosus
Evolvulus_alsinoides
Boea_hygrometrica|NC_016741
Nicotiana_tabacum|NC_006581
Solanum_lycopersicum|NC_035963
Solanum_melongena|NC_050334
Platycodon_grandiflorus|NC_035958
Castilleja_paramensis|NC_031806
Olea_europaea|MG372118
Utricularia_reniformis|NC_034982
Ziziphus_jujuba|NC_029809
Citrullus_lanatus|NC_014043
Citrus_sinensis|NC_037463
Vaccinium_macrocarpon|NC_023338
Asclepias_syriaca|NC_022796
Nepenthes_ventricosa|NC_039531
Silene_latifolia|NC_014487
Beta_vulgaris|NC_002511
Arabidopsis_thaliana|Y08501
Carica_papaya|NC_012116
Daucus_carota|NC_017855
Gossypium_arboreum|NC_035073
Manihot_esculenta|MK176513
Populus_alba|MK034705
Lagerstroemia_indica|NC_035616
Eucalyptus_grandis|NC_040010
Medicago_truncatula|NC_029641
Malus_domestica|FR714868
Cannabis_sativa|NC_029855
Vitis_vinifera|NC_012119
Liriodendron_tulipifera|NC_021152
Nelumbo_nucifera|NC_030753
Nymphaea_colorata|NC_037468
Schisandra_sphenanthera|MH748549
Spirodela_polyrhiza|NC_017840
Zea_mays|NC_007982
Oryza_sativa|NC_011033
Allium_cepa|NC_030100
Cocos_nucifera|NC_031696
Cycas_taitungensis|NC_010303
Ginkgo_biloba|NC_027976
0.02

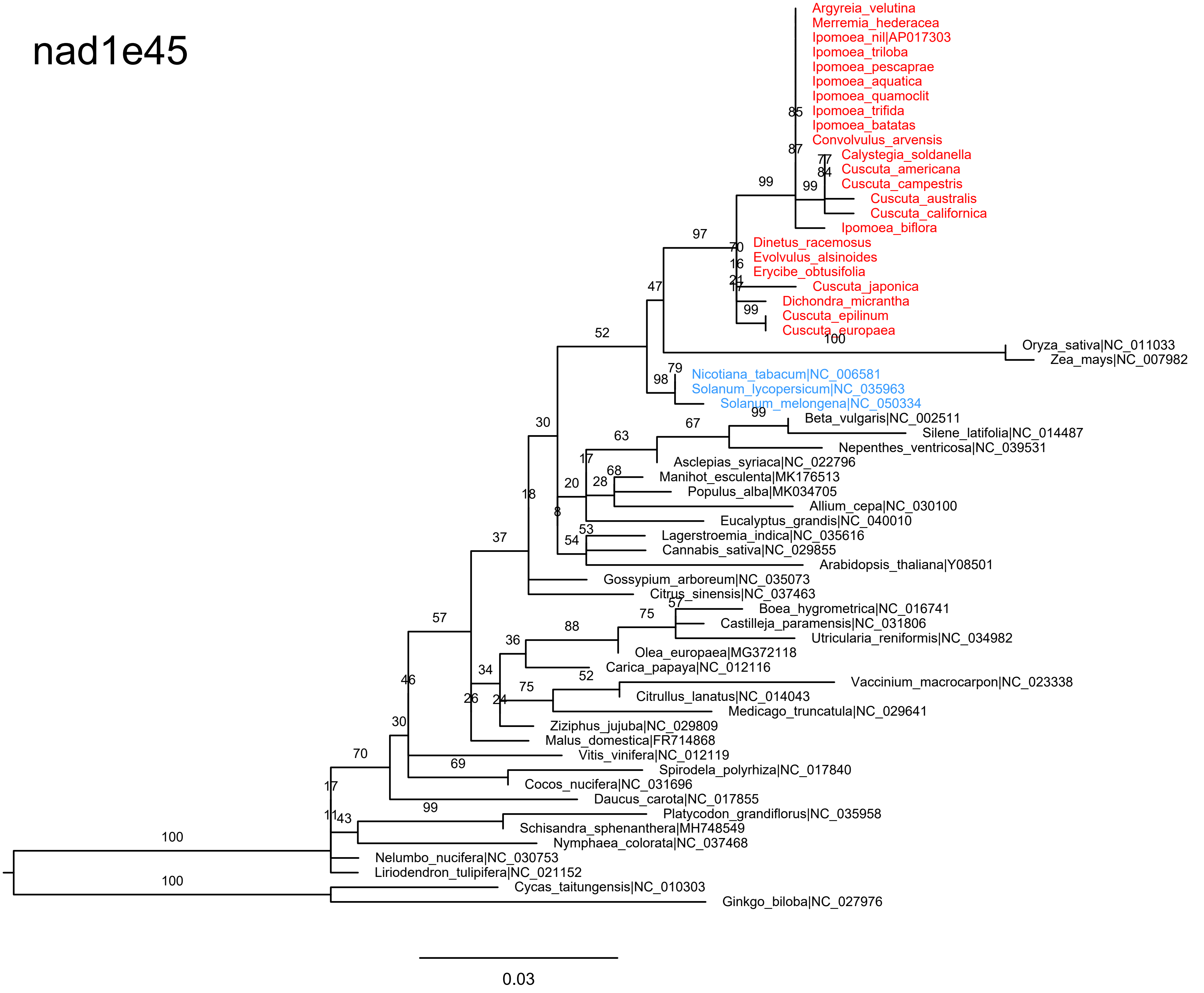
nad1e45
Argyreia_velutina
Merremia_hederacea
Ipomoea_nil|AP017303
Ipomoea_triloba
Ipomoea_pescaprae
Ipomoea_aquatica
Ipomoea_quamoclit
Ipomoea_trifida
Ipomoea_batatas
Convolvulus_arvensis
Calystegia_soldanella
Cuscuta_americana
Cuscuta_campestris
Cuscuta_australis
Cuscuta_californica
Ipomoea_biflora
Dinetus_racemosus
Evolvulus_alsinoides
Erycibe_obtusifolia
Cuscuta_japonica
Dichondra_micrantha
Cuscuta_epilinum
Cuscuta_europaea
Oryza_sativa|NC_011033
Zea_mays|NC_007982
Nicotiana_tabacum|NC_006581
Solanum_lycopersicum|NC_035963
Solanum_melongena|NC_050334
Beta_vulgaris|NC_002511
Silene_latifolia|NC_014487
Nepenthes_ventricosa|NC_039531
Asclepias_syriaca|NC_022796
Manihot_esculenta|MK176513
Populus_alba|MK034705
Allium_cepa|NC_030100
Eucalyptus_grandis|NC_040010
Lagerstroemia_indica|NC_035616
Cannabis_sativa|NC_029855
Arabidopsis_thaliana|Y08501
Gossypium_arboreum|NC_035073
Citrus_sinensis|NC_037463
Boea_hygrometrica|NC_016741
Castilleja_paramensis|NC_031806
Utricularia_reniformis|NC_034982
Olea_europaea|MG372118
Carica_papaya|NC_012116
Vaccinium_macrocarpon|NC_023338
Citrullus_lanatus|NC_014043
Medicago_truncatula|NC_029641
Ziziphus_jujuba|NC_029809
Malus_domestica|FR714868
Vitis_vinifera|NC_012119
Spirodela_polyrhiza|NC_017840
Cocos_nucifera|NC_031696
Daucus_carota|NC_017855
Platycodon_grandiflorus|NC_035958
Schisandra_sphenanthera|MH748549
Nymphaea_colorata|NC_037468
Nelumbo_nucifera|NC_030753
Liriodendron_tulipifera|NC_021152
Cycas_taitungensis|NC_010303
Ginkgo_biloba|NC_027976
0.03

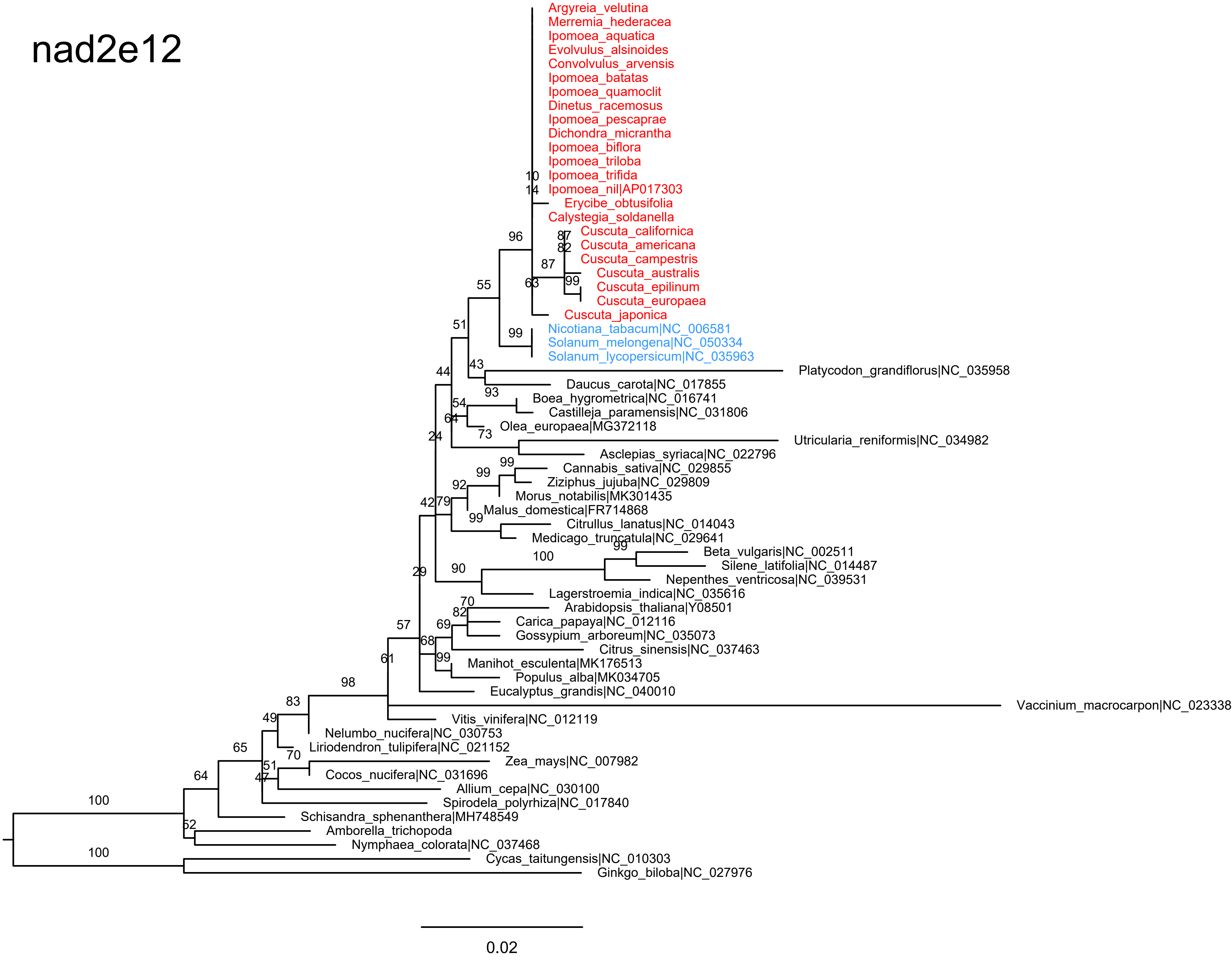

nad2e12
Argyreia_velutina
Merremia_hederacea
Ipomoea_aquatica
Evolvulus_alsinoides
Convolvulus_arvensis
Ipomoea_batatas
Ipomoea_quamoclit
Dinetus_racemosus
Ipomoea_pescaprae
Dichondra_micrantha
Ipomoea_biflora
Ipomoea_triloba
Ipomoea_trifida
Ipomoea_nil|AP017303
Erycibe_obtusifolia
Calystegia_soldanella
Cuscuta_californica
Cuscuta_americana
Cuscuta_campestris
Cuscuta_australis
Cuscuta_epilinum
Cuscuta_europaea
Cuscuta_japonica
Nicotiana_tabacum|NC_006581
Solanum_melongena|NC_050334
Solanum_lycopersicum|NC_035963
Platycodon_grandiflorus|NC_035958
Daucus_carota|NC_017855
Boea_hygrometrica|NC_016741
Castilleja_paramensis|NC_031806
Olea_europaea|MG372118
Utricularia_reniformis|NC_034982
Asclepias_syriaca|NC_022796
Cannabis_sativa|NC_029855
Ziziphus_jujuba|NC_029809
Morus_notabilis|MK301435
Malus_domestica|FR714868
Citrullus_lanatus|NC_014043
Medicago_truncatula|NC_029641
Beta_vulgaris|NC_002511
Silene_latifolia|NC_014487
Nepenthes_ventricosa|NC_039531
Lagerstroemia_indica|NC_035616
Arabidopsis_thaliana|Y08501
Carica_papaya|NC_012116
Gossypium_arboreum|NC_035073
Citrus_sinensis|NC_037463
Manihot_esculenta|MK176513
Populus_alba|MK034705
Eucalyptus_grandis|NC_040010
Vaccinium_macrocarpon|NC_023338
Vitis_vinifera|NC_012119
Nelumbo_nucifera|NC_030753
Liriodendron_tulipifera|NC_021152
Zea_mays|NC_007982
Cocos_nucifera|NC_031696
Allium_cepa|NC_030100
Spirodela_polyrhiza|NC_017840
Schisandra_sphenanthera|MH748549
Amborella_trichopoda
Nymphaea_colorata|NC_037468
Cycas_taitungensis|NC_010303
Ginkgo_biloba|NC_027976
0.02

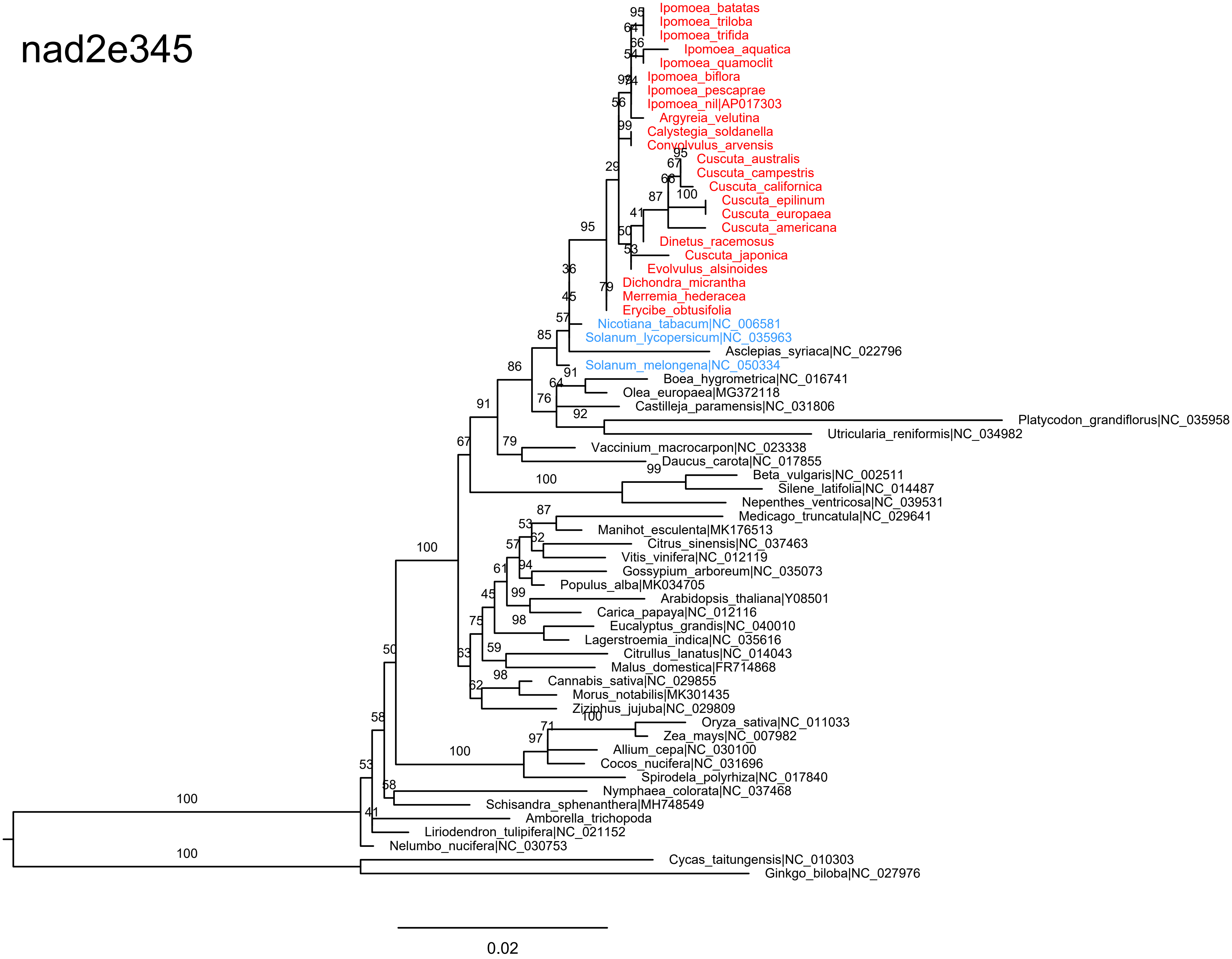
nad2e345
Ipomoea_batatas
Ipomoea_triloba
Ipomoea_trifida
Ipomoea_aquatica
Ipomoea_quamoclit
Ipomoea_biflora
Ipomoea_pescaprae
Ipomoea_nil|AP017303
Argyreia_velutina
Calystegia_soldanella
Convolvulus_arvensis
Cuscuta_australis
Cuscuta_campestris
Cuscuta_californica
Cuscuta_epilinum
Cuscuta_europaea
Cuscuta_americana
Dinetus_racemosus
Cuscuta_japonica
Evolvulus_alsinoides
Dichondra_micrantha
Merremia_hederacea
Erycibe_obtusifolia
Nicotiana_tabacum|NC_006581
Solanum_lycopersicum|NC_035963
Asclepias_syriaca|NC_022796
Solanum_melongena|NC_050334
Boea_hygrometrica|NC_016741
Olea_europaea|MG372118
Castilleja_paramensis|NC_031806
Platycodon_grandiflorus|NC_035958
Utricularia_reniformis|NC_034982
Vaccinium_macrocarpon|NC_023338
Daucus_carota|NC_017855
Beta_vulgaris|NC_002511
Silene_latifolia|NC_014487
Nepenthes_ventricosa|NC_039531
Medicago_truncatula|NC_029641
Manihot_esculenta|MK176513
Citrus_sinensis|NC_037463
Vitis_vinifera|NC_012119
Gossypium_arboreum|NC_035073
Populus_alba|MK034705
Arabidopsis_thaliana|Y08501
Carica_papaya|NC_012116
Eucalyptus_grandis|NC_040010
Lagerstroemia_indica|NC_035616
Citrullus_lanatus|NC_014043
Malus_domestica|FR714868
Cannabis_sativa|NC_029855
Morus_notabilis|MK301435
Ziziphus_jujuba|NC_029809
Oryza_sativa|NC_011033
Zea_mays|NC_007982
Allium_cepa|NC_030100
Cocos_nucifera|NC_031696
Spirodela_polyrhiza|NC_017840
Nymphaea_colorata|NC_037468
Schisandra_sphenanthera|MH748549
Amborella_trichopoda
Liriodendron_tulipifera|NC_021152
Nelumbo_nucifera|NC_030753
Cycas_taitungensis|NC_010303
Ginkgo_biloba|NC_027976
0.02

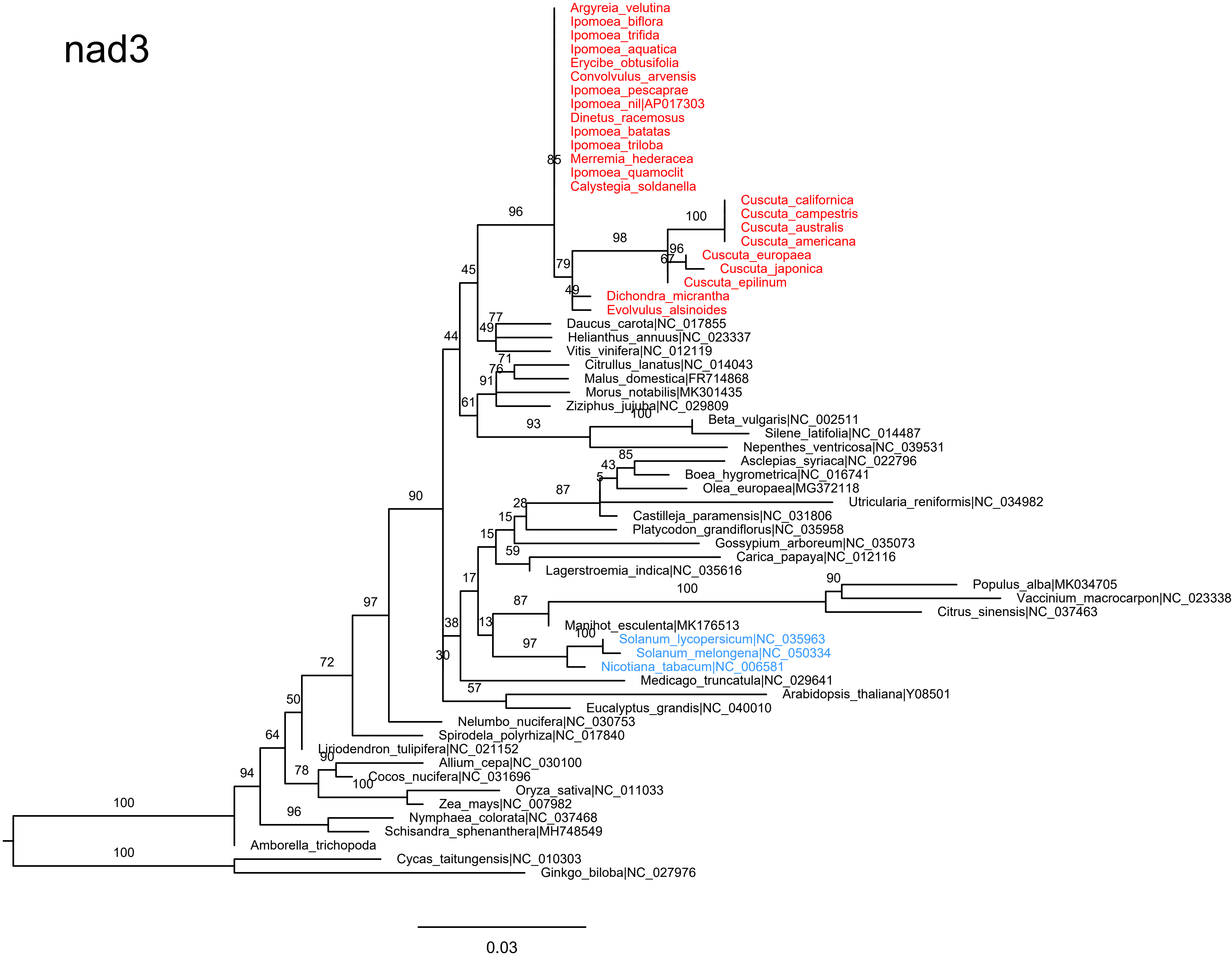
nad3
Argyreia_velutina
Ipomoea_biflora
Ipomoea_trifida
Ipomoea_aquatica
Erycibe_obtusifolia
Convolvulus_arvensis
Ipomoea_pescaprae
Ipomoea_nil|AP017303
Dinetus_racemosus
Ipomoea_batatas
Ipomoea_triloba
Merremia_hederacea
Ipomoea_quamoclit
Calystegia_soldanella
Cuscuta_californica
Cuscuta_campestris
Cuscuta_australis
Cuscuta_americana
Cuscuta_europaea
Cuscuta_japonica
Cuscuta_epilinum
Dichondra_micrantha
Evolvulus_alsinoides
Daucus_carota|NC_017855
Helianthus_annuus|NC_023337
Vitis_vinifera|NC_012119
Citrullus_lanatus|NC_014043
Malus_domestica|FR714868
Morus_notabilis|MK301435
Ziziphus_jujuba|NC_029809
Beta_vulgaris|NC_002511
Silene_latifolia|NC_014487
Nepenthes_ventricosa|NC_039531
Asclepias_syriaca|NC_022796
Boea_hygrometrica|NC_016741
Olea_europaea|MG372118
Utricularia_reniformis|NC_034982
Castilleja_paramensis|NC_031806
Platycodon_grandiflorus|NC_035958
Gossypium_arboreum|NC_035073
Carica_papaya|NC_012116
Lagerstroemia_indica|NC_035616
Populus_alba|MK034705
Vaccinium_macrocarpon|NC_023338
Citrus_sinensis|NC_037463
Manihot_esculenta|MK176513
Solanum_lycopersicum|NC_035963
Solanum_melongena|NC_050334
Nicotiana_tabacum|NC_006581
Medicago_truncatula|NC_029641
Arabidopsis_thaliana|Y08501
Eucalyptus_grandis|NC_040010
Nelumbo_nucifera|NC_030753
Spirodela_polyrhiza|NC_017840
Liriodendron_tulipifera|NC_021152
Allium_cepa|NC_030100
Cocos_nucifera|NC_031696
Oryza_sativa|NC_011033
Zea_mays|NC_007982
Nymphaea_colorata|NC_037468
Schisandra_sphenanthera|MH748549
Amborella_trichopoda
Cycas_taitungensis|NC_010303
Ginkgo_biloba|NC_027976
0.03

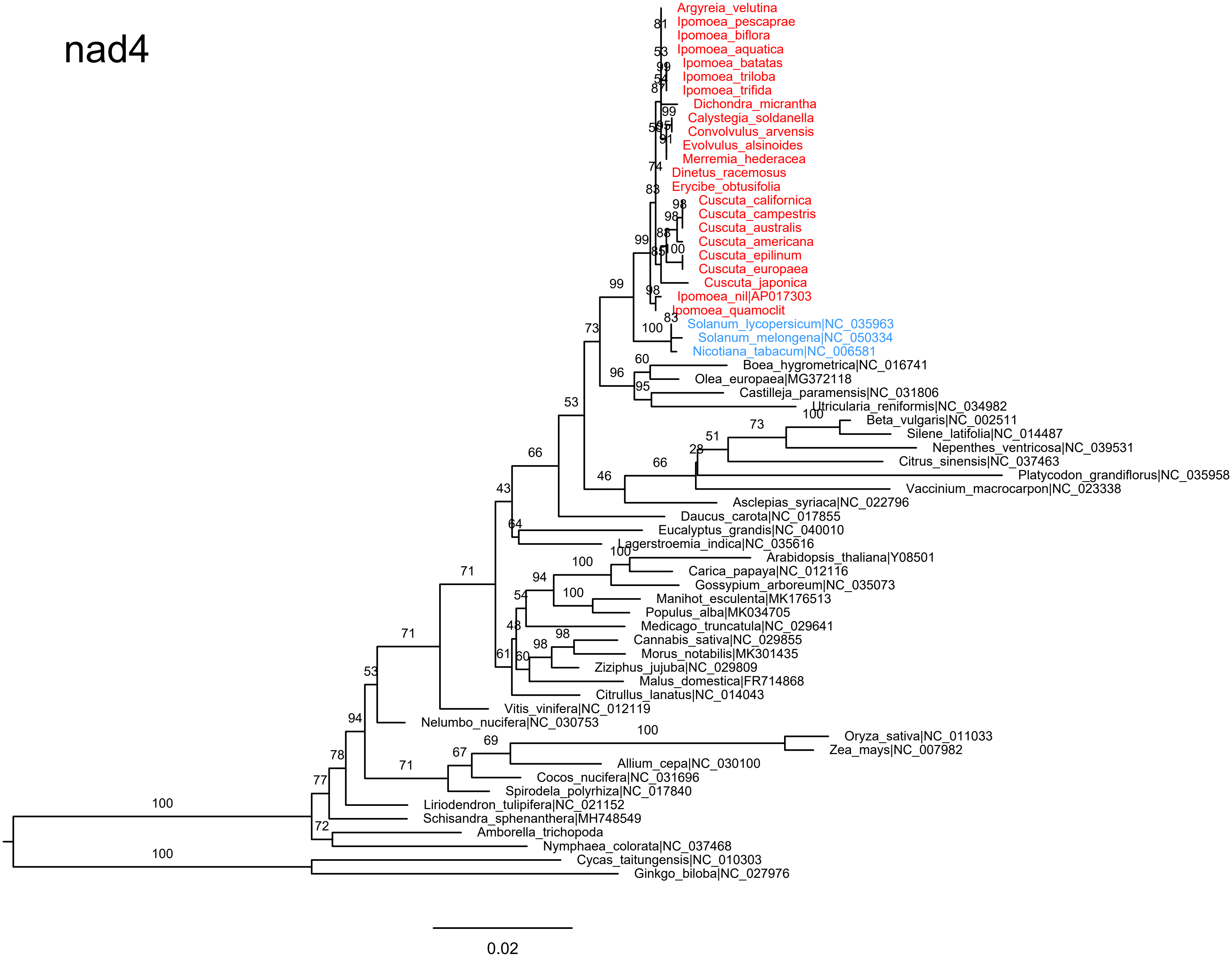
nad4
Argyreia_velutina
Ipomoea_pescaprae
Ipomoea_biflora
Ipomoea_aquatica
Ipomoea_batatas
Ipomoea_triloba
Ipomoea_trifida
Dichondra_micrantha
Calystegia_soldanella
Convolvulus_arvensis
Evolvulus_alsinoides
Merremia_hederacea
Dinetus_racemosus
Erycibe_obtusifolia
Cuscuta_californica
Cuscuta_campestris
Cuscuta_australis
Cuscuta_americana
Cuscuta_epilinum
Cuscuta_europaea
Cuscuta_japonica
Ipomoea_nil|AP017303
Ipomoea_quamoclit
Solanum_lycopersicum|NC_035963
Solanum_melongena|NC_050334
Nicotiana_tabacum|NC_006581
Boea_hygrometrica|NC_016741
Olea_europaea|MG372118
Castilleja_paramensis|NC_031806
Utricularia_reniformis|NC_034982
Beta_vulgaris|NC_002511
Silene_latifolia|NC_014487
Nepenthes_ventricosa|NC_039531
Citrus_sinensis|NC_037463
Platycodon_grandiflorus|NC_035958
Vaccinium_macrocarpon|NC_023338
Asclepias_syriaca|NC_022796
Daucus_carota|NC_017855
Eucalyptus_grandis|NC_040010
Lagerstroemia_indica|NC_035616
Arabidopsis_thaliana|Y08501
Carica_papaya|NC_012116
Gossypium_arboreum|NC_035073
Manihot_esculenta|MK176513
Populus_alba|MK034705
Medicago_truncatula|NC_029641
Cannabis_sativa|NC_029855
Morus_notabilis|MK301435
Ziziphus_jujuba|NC_029809
Malus_domestica|FR714868
Citrullus_lanatus|NC_014043
Vitis_vinifera|NC_012119
Nelumbo_nucifera|NC_030753
Oryza_sativa|NC_011033
Zea_mays|NC_007982
Allium_cepa|NC_030100
Cocos_nucifera|NC_031696
Spirodela_polyrhiza|NC_017840
Liriodendron_tulipifera|NC_021152
Schisandra_sphenanthera|MH748549
Amborella_trichopoda
Nymphaea_colorata|NC_037468
Cycas_taitungensis|NC_010303
Ginkgo_biloba|NC_027976
0.02

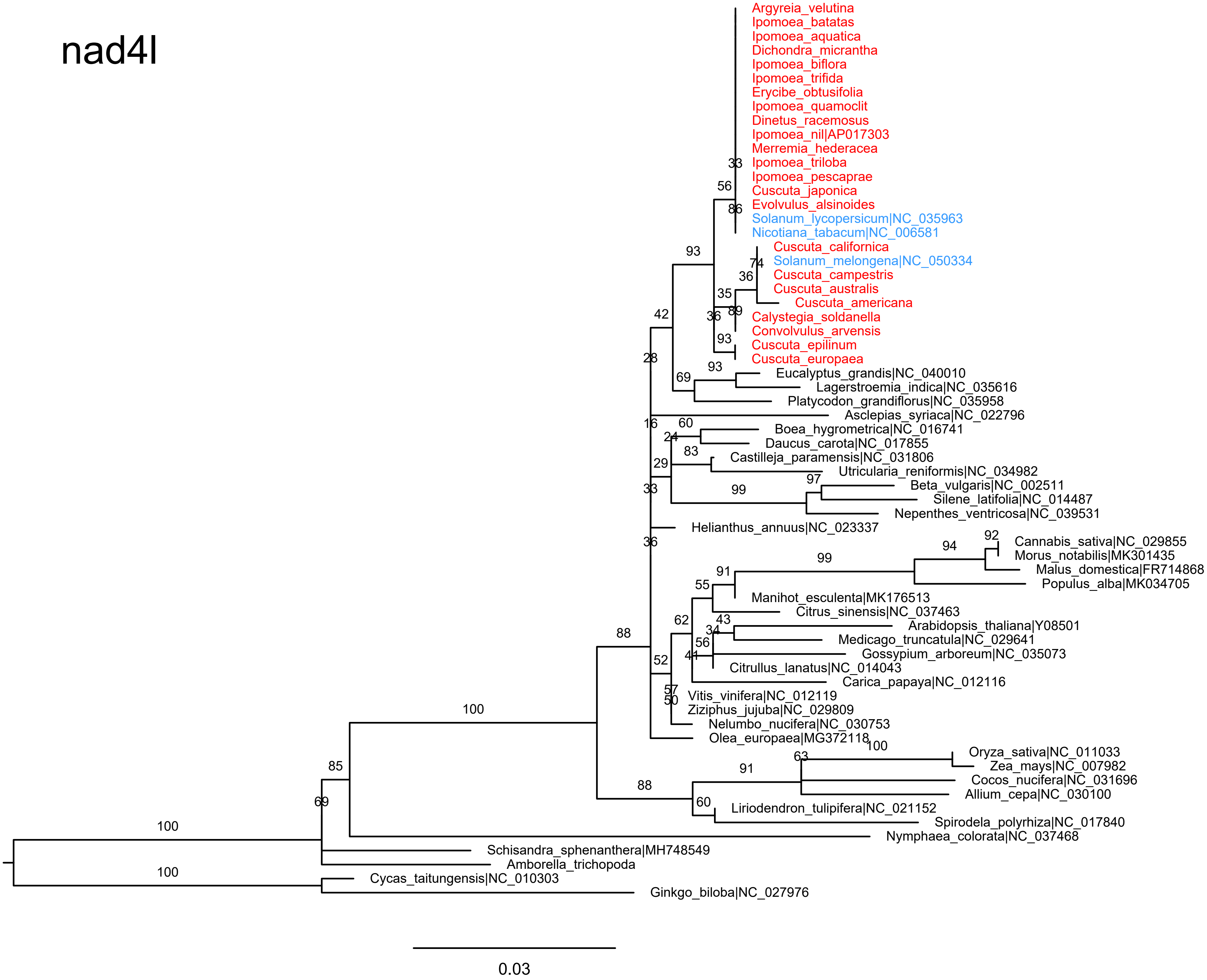
nad4l
Argyreia_velutina
Ipomoea_batatas
Ipomoea_aquatica
Dichondra_micrantha
Ipomoea_biflora
Ipomoea_trifida
Erycibe_obtusifolia
Ipomoea_quamoclit
Dinetus_racemosus
Ipomoea_nil|AP017303
Merremia_hederacea
Ipomoea_triloba
Ipomoea_pescaprae
Cuscuta_japonica
Evolvulus_alsinoides
Solanum_lycopersicum|NC_035963
Nicotiana_tabacum|NC_006581
Cuscuta_californica
Solanum_melongena|NC_050334
Cuscuta_campestris
Cuscuta_australis
Cuscuta_americana
Calystegia_soldanella
Convolvulus_arvensis
Cuscuta_epilinum
Cuscuta_europaea
Eucalyptus_grandis|NC_040010
Lagerstroemia_indica|NC_035616
Platycodon_grandiflorus|NC_035958
Asclepias_syriaca|NC_022796
Boea_hygrometrica|NC_016741
Daucus_carota|NC_017855
Castilleja_paramensis|NC_031806
Utricularia_reniformis|NC_034982
Beta_vulgaris|NC_002511
Silene_latifolia|NC_014487
Nepenthes_ventricosa|NC_039531
Helianthus_annuus|NC_023337
Cannabis_sativa|NC_029855
Morus_notabilis|MK301435
Malus_domestica|FR714868
Populus_alba|MK034705
Manihot_esculenta|MK176513
Citrus_sinensis|NC_037463
Arabidopsis_thaliana|Y08501
Medicago_truncatula|NC_029641
Gossypium_arboreum|NC_035073
Citrullus_lanatus|NC_014043
Carica_papaya|NC_012116
Vitis_vinifera|NC_012119
Ziziphus_jujuba|NC_029809
Nelumbo_nucifera|NC_030753
Olea_europaea|MG372118
Oryza_sativa|NC_011033
Zea_mays|NC_007982
Cocos_nucifera|NC_031696
Allium_cepa|NC_030100
Liriodendron_tulipifera|NC_021152
Spirodela_polyrhiza|NC_017840
Nymphaea_colorata|NC_037468
Schisandra_sphenanthera|MH748549
Amborella_trichopoda
Cycas_taitungensis|NC_010303
Ginkgo_biloba|NC_027976
0.03

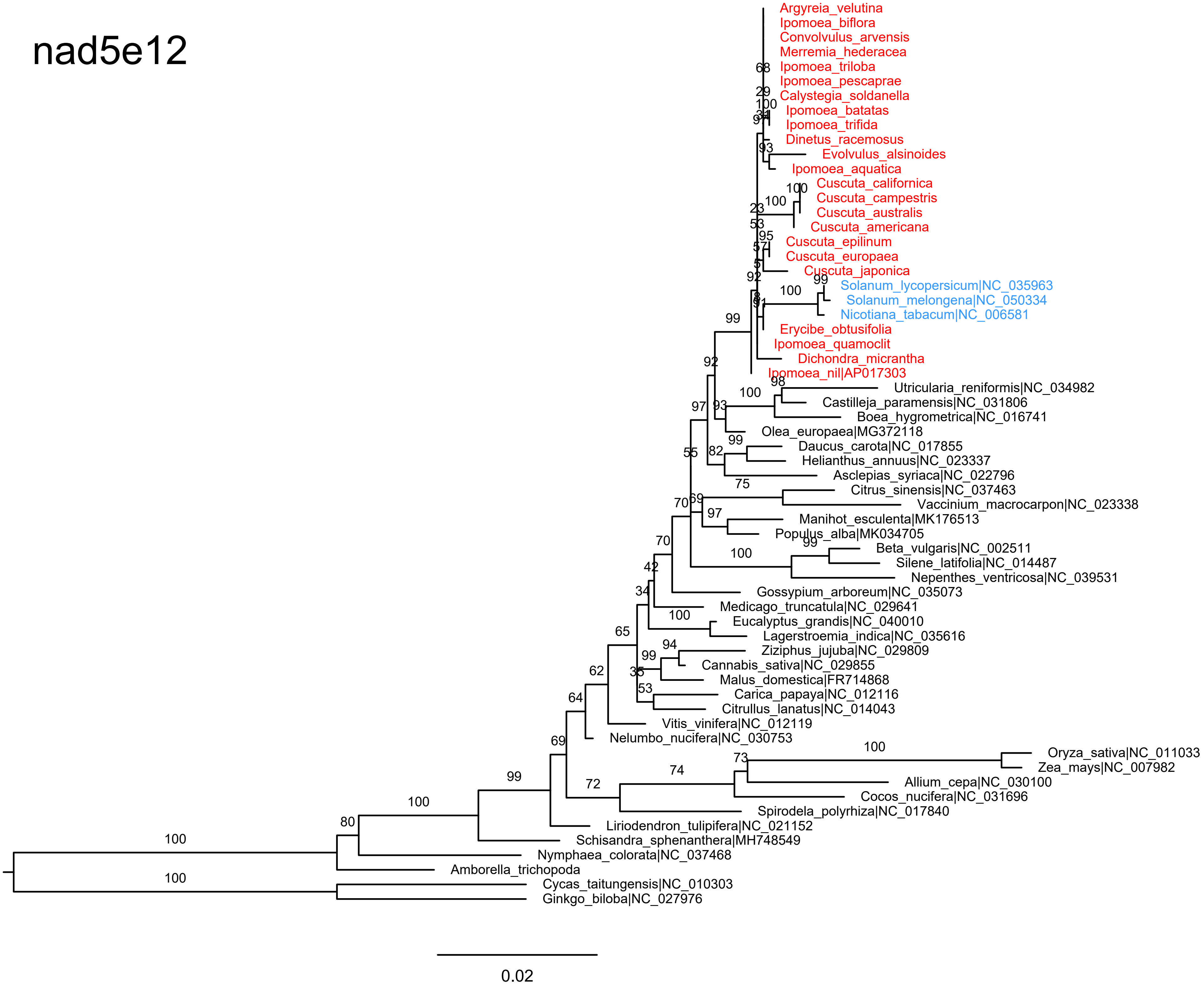

nad5e12
Argyreia_velutina
Ipomoea_biflora
Convolvulus_arvensis
Merremia_hederacea
Ipomoea_triloba
Ipomoea_pescaprae
Calystegia_soldanella
Ipomoea_batatas
Ipomoea_trifida
Dinetus_racemosus
Evolvulus_alsinoides
Ipomoea_aquatica
Cuscuta_californica
Cuscuta_campestris
Cuscuta_australis
Cuscuta_americana
Cuscuta_epilinum
Cuscuta_europaea
Cuscuta_japonica
Solanum_lycopersicum|NC_035963
Solanum_melongena|NC_050334
Nicotiana_tabacum|NC_006581
Erycibe_obtusifolia
Ipomoea_quamoclit
Dichondra_micrantha
Ipomoea_nil|AP017303
Utricularia_reniformis|NC_034982
Castilleja_paramensis|NC_031806
Boea_hygrometrica|NC_016741
Olea_europaea|MG372118
Daucus_carota|NC_017855
Helianthus_annuus|NC_023337
Asclepias_syriaca|NC_022796
Citrus_sinensis|NC_037463
Vaccinium_macrocarpon|NC_023338
Manihot_esculenta|MK176513
Populus_alba|MK034705
Beta_vulgaris|NC_002511
Silene_latifolia|NC_014487
Nepenthes_ventricosa|NC_039531
Gossypium_arboreum|NC_035073
Medicago_truncatula|NC_029641
Eucalyptus_grandis|NC_040010
Lagerstroemia_indica|NC_035616
Ziziphus_jujuba|NC_029809
Cannabis_sativa|NC_029855
Malus_domestica|FR714868
Carica_papaya|NC_012116
Citrullus_lanatus|NC_014043
Vitis_vinifera|NC_012119
Nelumbo_nucifera|NC_030753
Oryza_sativa|NC_011033
Zea_mays|NC_007982
Allium_cepa|NC_030100
Cocos_nucifera|NC_031696
Spirodela_polyrhiza|NC_017840
Liriodendron_tulipifera|NC_021152
Schisandra_sphenanthera|MH748549
Nymphaea_colorata|NC_037468
Amborella_trichopoda
Cycas_taitungensis|NC_010303
Ginkgo_biloba|NC_027976
0.02

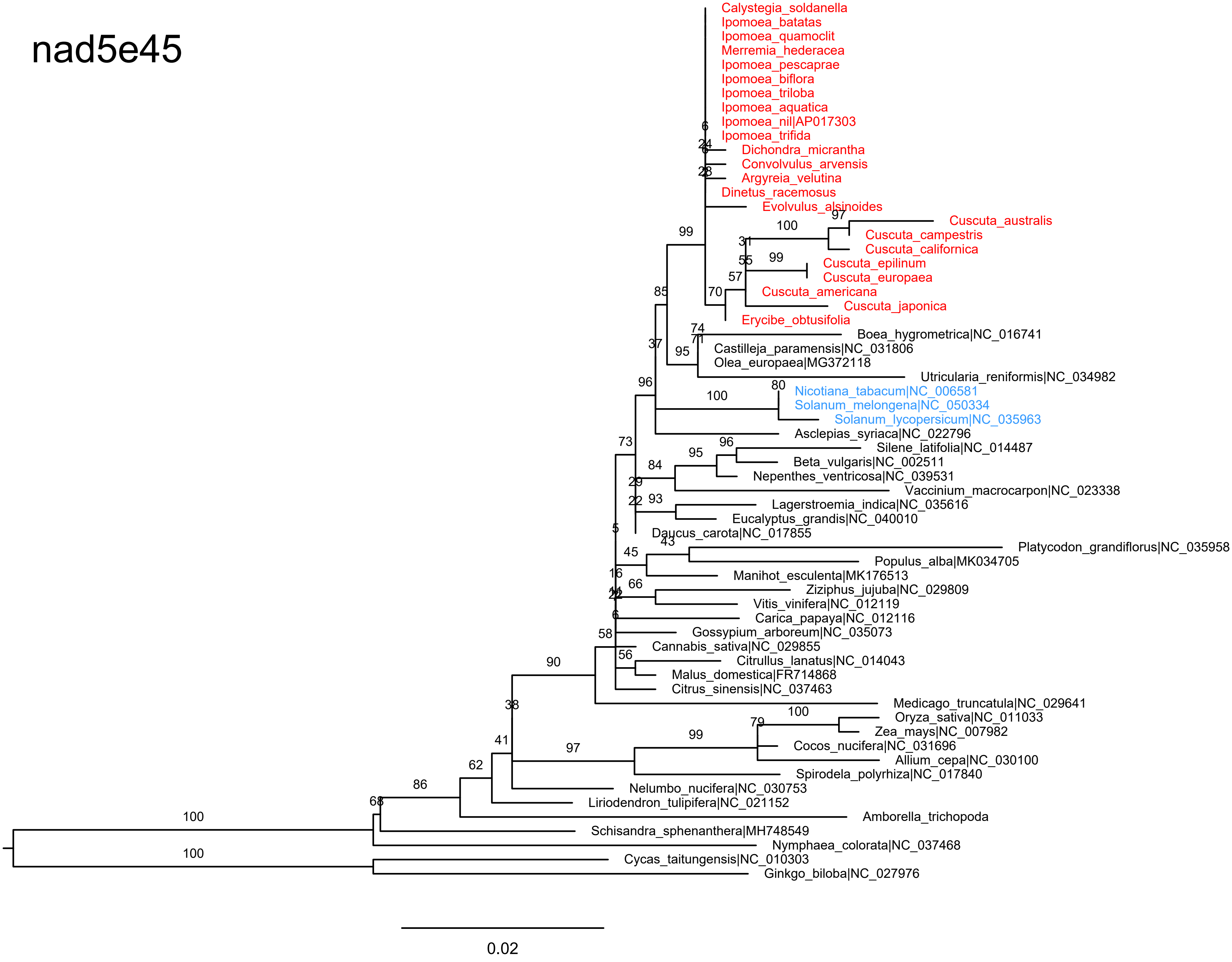
nad5e45
Calystegia_soldanella
Ipomoea_batatas
Ipomoea_quamoclit
Merremia_hederacea
Ipomoea_pescaprae
Ipomoea_biflora
Ipomoea_triloba
Ipomoea_aquatica
Ipomoea_nil|AP017303
Ipomoea_trifida
Dichondra_micrantha
Convolvulus_arvensis
Argyreia_velutina
Dinetus_racemosus
Evolvulus_alsinoides
Cuscuta_australis
Cuscuta_campestris
Cuscuta_californica
Cuscuta_epilinum
Cuscuta_europaea
Cuscuta_americana
Cuscuta_japonica
Erycibe_obtusifolia
Boea_hygrometrica|NC_016741
Castilleja_paramensis|NC_031806
Olea_europaea|MG372118
Utricularia_reniformis|NC_034982
Nicotiana_tabacum|NC_006581
Solanum_melongena|NC_050334
Solanum_lycopersicum|NC_035963
Asclepias_syriaca|NC_022796
Silene_latifolia|NC_014487
Beta_vulgaris|NC_002511
Nepenthes_ventricosa|NC_039531
Vaccinium_macrocarpon|NC_023338
Lagerstroemia_indica|NC_035616
Eucalyptus_grandis|NC_040010
Daucus_carota|NC_017855
Platycodon_grandiflorus|NC_035958
Populus_alba|MK034705
Manihot_esculenta|MK176513
Ziziphus_jujuba|NC_029809
Vitis_vinifera|NC_012119
Carica_papaya|NC_012116
Gossypium_arboreum|NC_035073
Cannabis_sativa|NC_029855
Citrullus_lanatus|NC_014043
Malus_domestica|FR714868
Citrus_sinensis|NC_037463
Medicago_truncatula|NC_029641
Oryza_sativa|NC_011033
Zea_mays|NC_007982
Cocos_nucifera|NC_031696
Allium_cepa|NC_030100
Spirodela_polyrhiza|NC_017840
Nelumbo_nucifera|NC_030753
Liriodendron_tulipifera|NC_021152
Amborella_trichopoda
Schisandra_sphenanthera|MH748549
Nymphaea_colorata|NC_037468
Cycas_taitungensis|NC_010303
Ginkgo_biloba|NC_027976
0.02

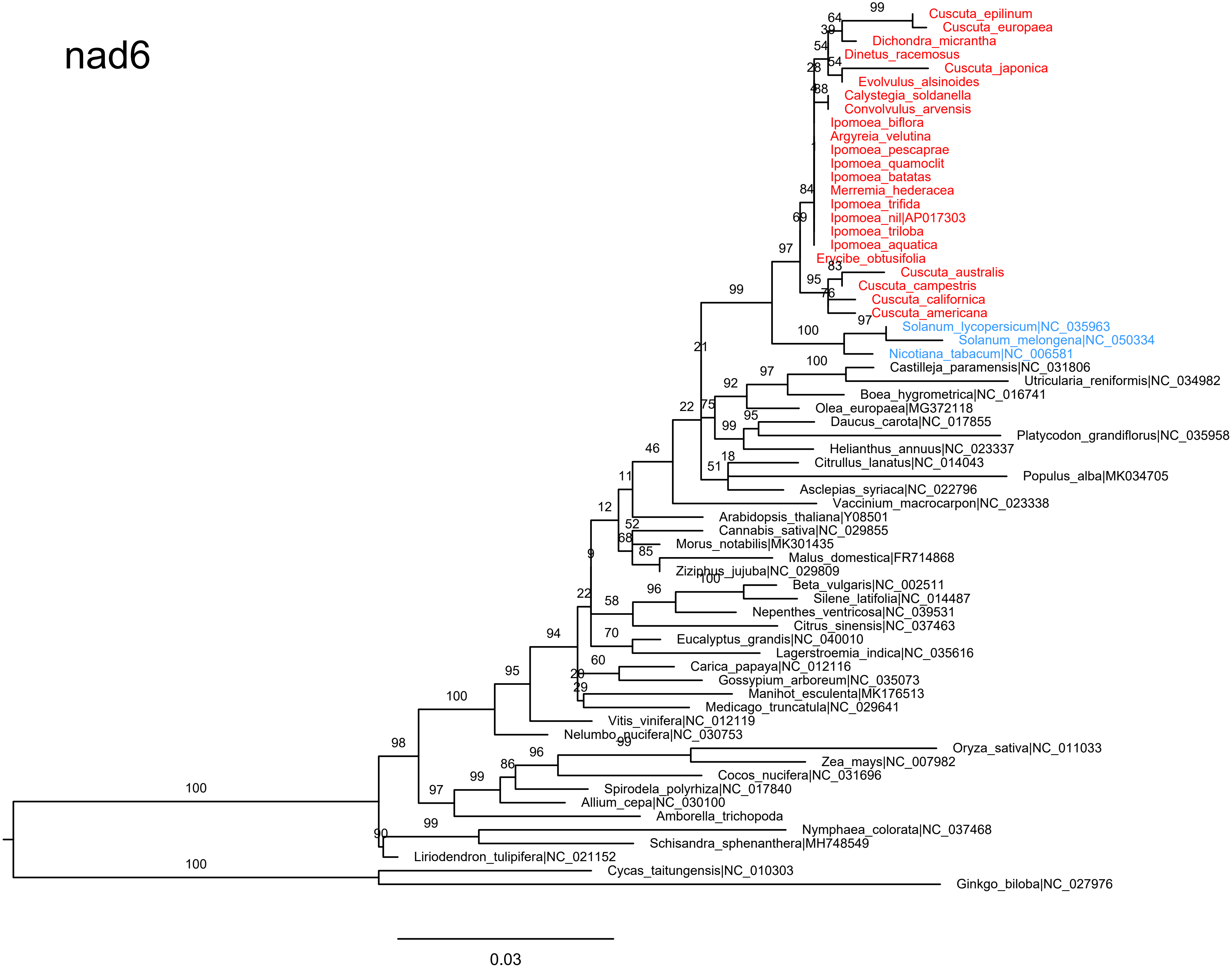
nad6
Cuscuta_epilinum
Cuscuta_europaea
Dichondra_micrantha
Dinetus_racemosus
Cuscuta_japonica
Evolvulus_alsinoides
Calystegia_soldanella
Convolvulus_arvensis
Ipomoea_biflora
Argyreia_velutina
Ipomoea_pescaprae
Ipomoea_quamoclit
Ipomoea_batatas
Merremia_hederacea
Ipomoea_trifida
Ipomoea_nil|AP017303
Ipomoea_triloba
Ipomoea_aquatica
Erycibe_obtusifolia
Cuscuta_australis
Cuscuta_campestris
Cuscuta_californica
Cuscuta_americana
Solanum_lycopersicum|NC_035963
Solanum_melongena|NC_050334
Nicotiana_tabacum|NC_006581
Castilleja_paramensis|NC_031806
Utricularia_reniformis|NC_034982
Boea_hygrometrica|NC_016741
Olea_europaea|MG372118
Daucus_carota|NC_017855
Platycodon_grandiflorus|NC_035958
Helianthus_annuus|NC_023337
Citrullus_lanatus|NC_014043
Populus_alba|MK034705
Asclepias_syriaca|NC_022796
Vaccinium_macrocarpon|NC_023338
Arabidopsis_thaliana|Y08501
Cannabis_sativa|NC_029855
Morus_notabilis|MK301435
Malus_domestica|FR714868
Ziziphus_jujuba|NC_029809
Beta_vulgaris|NC_002511
Silene_latifolia|NC_014487
Nepenthes_ventricosa|NC_039531
Citrus_sinensis|NC_037463
Eucalyptus_grandis|NC_040010
Lagerstroemia_indica|NC_035616
Carica_papaya|NC_012116
Gossypium_arboreum|NC_035073
Manihot_esculenta|MK176513
Medicago_truncatula|NC_029641
Vitis_vinifera|NC_012119
Nelumbo_nucifera|NC_030753
Oryza_sativa|NC_011033
Zea_mays|NC_007982
Cocos_nucifera|NC_031696
Spirodela_polyrhiza|NC_017840
Allium_cepa|NC_030100
Amborella_trichopoda
Nymphaea_colorata|NC_037468
Schisandra_sphenanthera|MH748549
Liriodendron_tulipifera|NC_021152
Cycas_taitungensis|NC_010303
Ginkgo_biloba|NC_027976
0.03

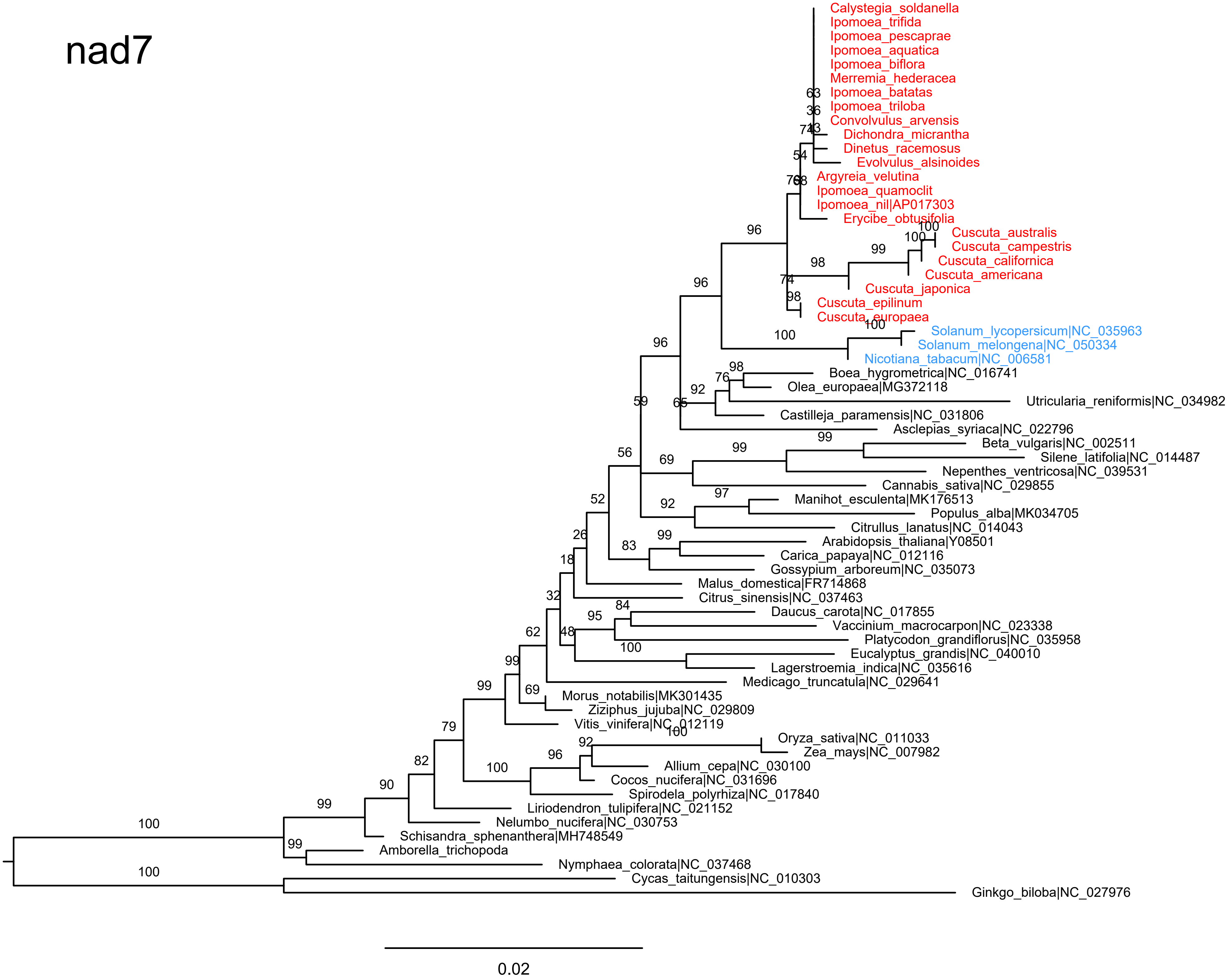
nad7
Calystegia_soldanella
Ipomoea_trifida
Ipomoea_pescaprae
Ipomoea_aquatica
Ipomoea_biflora
Merremia_hederacea
Ipomoea_batatas
Ipomoea_triloba
Convolvulus_arvensis
Dichondra_micrantha
Dinetus_racemosus
Evolvulus_alsinoides
Argyreia_velutina
Ipomoea_quamoclit
Ipomoea_nil|AP017303
Erycibe_obtusifolia
Cuscuta_australis
Cuscuta_campestris
Cuscuta_californica
Cuscuta_americana
Cuscuta_japonica
Cuscuta_epilinum
Cuscuta_europaea
Solanum_lycopersicum|NC_035963
Solanum_melongena|NC_050334
Nicotiana_tabacum|NC_006581
Boea_hygrometrica|NC_016741
Olea_europaea|MG372118
Utricularia_reniformis|NC_034982
Castilleja_paramensis|NC_031806
Asclepias_syriaca|NC_022796
Beta_vulgaris|NC_002511
Silene_latifolia|NC_014487
Nepenthes_ventricosa|NC_039531
Cannabis_sativa|NC_029855
Manihot_esculenta|MK176513
Populus_alba|MK034705
Citrullus_lanatus|NC_014043
Arabidopsis_thaliana|Y08501
Carica_papaya|NC_012116
Gossypium_arboreum|NC_035073
Malus_domestica|FR714868
Citrus_sinensis|NC_037463
Daucus_carota|NC_017855
Vaccinium_macrocarpon|NC_023338
Platycodon_grandiflorus|NC_035958
Eucalyptus_grandis|NC_040010
Lagerstroemia_indica|NC_035616
Medicago_truncatula|NC_029641
Morus_notabilis|MK301435
Ziziphus_jujuba|NC_029809
Vitis_vinifera|NC_012119
Oryza_sativa|NC_011033
Zea_mays|NC_007982
Allium_cepa|NC_030100
Cocos_nucifera|NC_031696
Spirodela_polyrhiza|NC_017840
Liriodendron_tulipifera|NC_021152
Nelumbo_nucifera|NC_030753
Schisandra_sphenanthera|MH748549
Amborella_trichopoda
Nymphaea_colorata|NC_037468
Cycas_taitungensis|NC_010303
Ginkgo_biloba|NC_027976
0.02

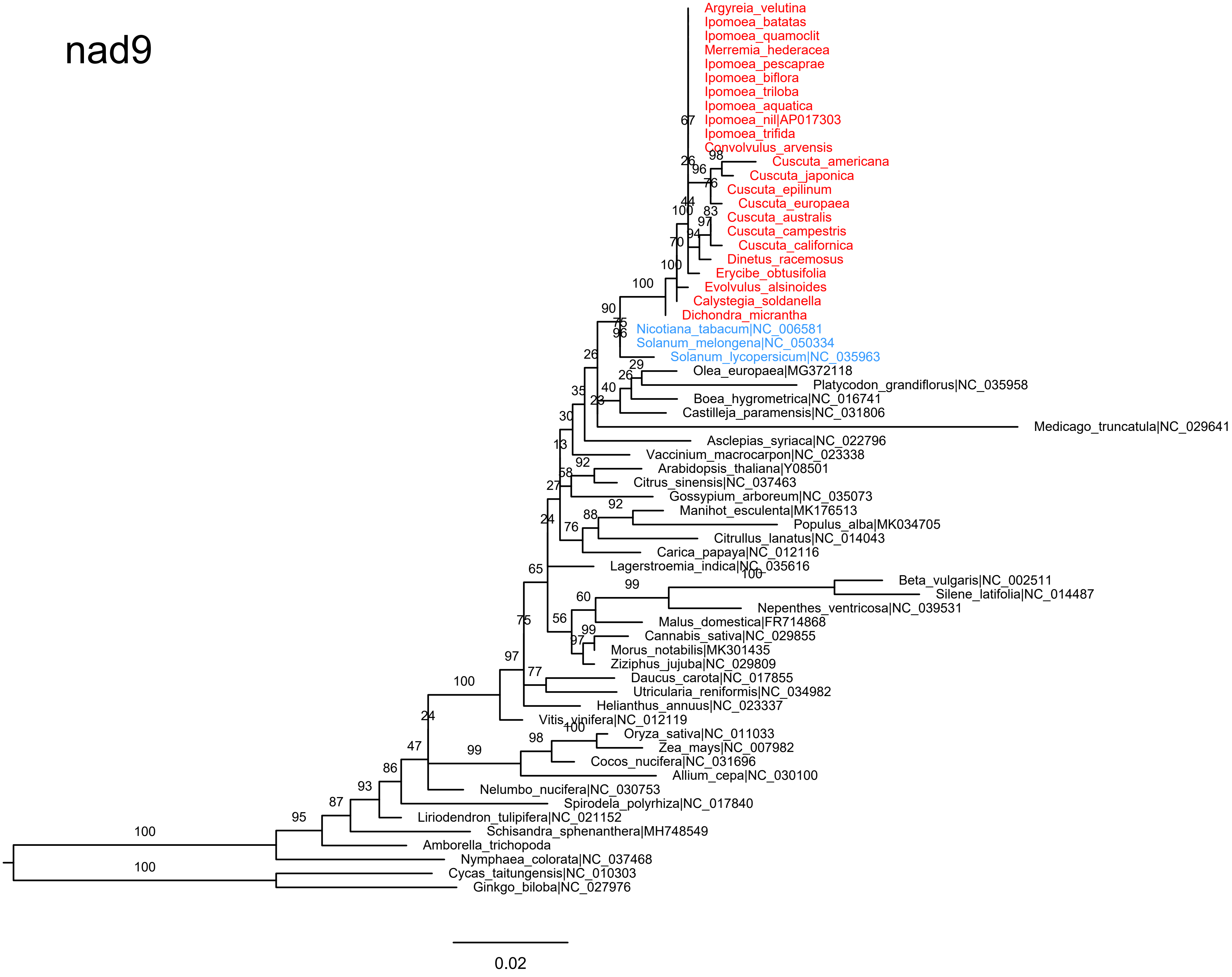
nad9
Argyreia_velutina
Ipomoea_batatas
Ipomoea_quamoclit
Merremia_hederacea
Ipomoea_pescaprae
Ipomoea_biflora
Ipomoea_triloba
Ipomoea_aquatica
Ipomoea_nil|AP017303
Ipomoea_trifida
Convolvulus_arvensis
Cuscuta_americana
Cuscuta_japonica
Cuscuta_epilinum
Cuscuta_europaea
Cuscuta_australis
Cuscuta_campestris
Cuscuta_californica
Dinetus_racemosus
Erycibe_obtusifolia
Evolvulus_alsinoides
Calystegia_soldanella
Dichondra_micrantha
Nicotiana_tabacum|NC_006581
Solanum_melongena|NC_050334
Solanum_lycopersicum|NC_035963
Olea_europaea|MG372118
Platycodon_grandiflorus|NC_035958
Boea_hygrometrica|NC_016741
Castilleja_paramensis|NC_031806
Medicago_truncatula|NC_029641
Asclepias_syriaca|NC_022796
Vaccinium_macrocarpon|NC_023338
Arabidopsis_thaliana|Y08501
Citrus_sinensis|NC_037463
Gossypium_arboreum|NC_035073
Manihot_esculenta|MK176513
Populus_alba|MK034705
Citrullus_lanatus|NC_014043
Carica_papaya|NC_012116
Lagerstroemia_indica|NC_035616
Beta_vulgaris|NC_002511
Silene_latifolia|NC_014487
Nepenthes_ventricosa|NC_039531
Malus_domestica|FR714868
Cannabis_sativa|NC_029855
Morus_notabilis|MK301435
Ziziphus_jujuba|NC_029809
Daucus_carota|NC_017855
Utricularia_reniformis|NC_034982
Helianthus_annuus|NC_023337
Vitis_vinifera|NC_012119
Oryza_sativa|NC_011033
Zea_mays|NC_007982
Cocos_nucifera|NC_031696
Allium_cepa|NC_030100
Nelumbo_nucifera|NC_030753
Spirodela_polyrhiza|NC_017840
Liriodendron_tulipifera|NC_021152
Schisandra_sphenanthera|MH748549
Amborella_trichopoda
Nymphaea_colorata|NC_037468
Cycas_taitungensis|NC_010303
Ginkgo_biloba|NC_027976
0.02

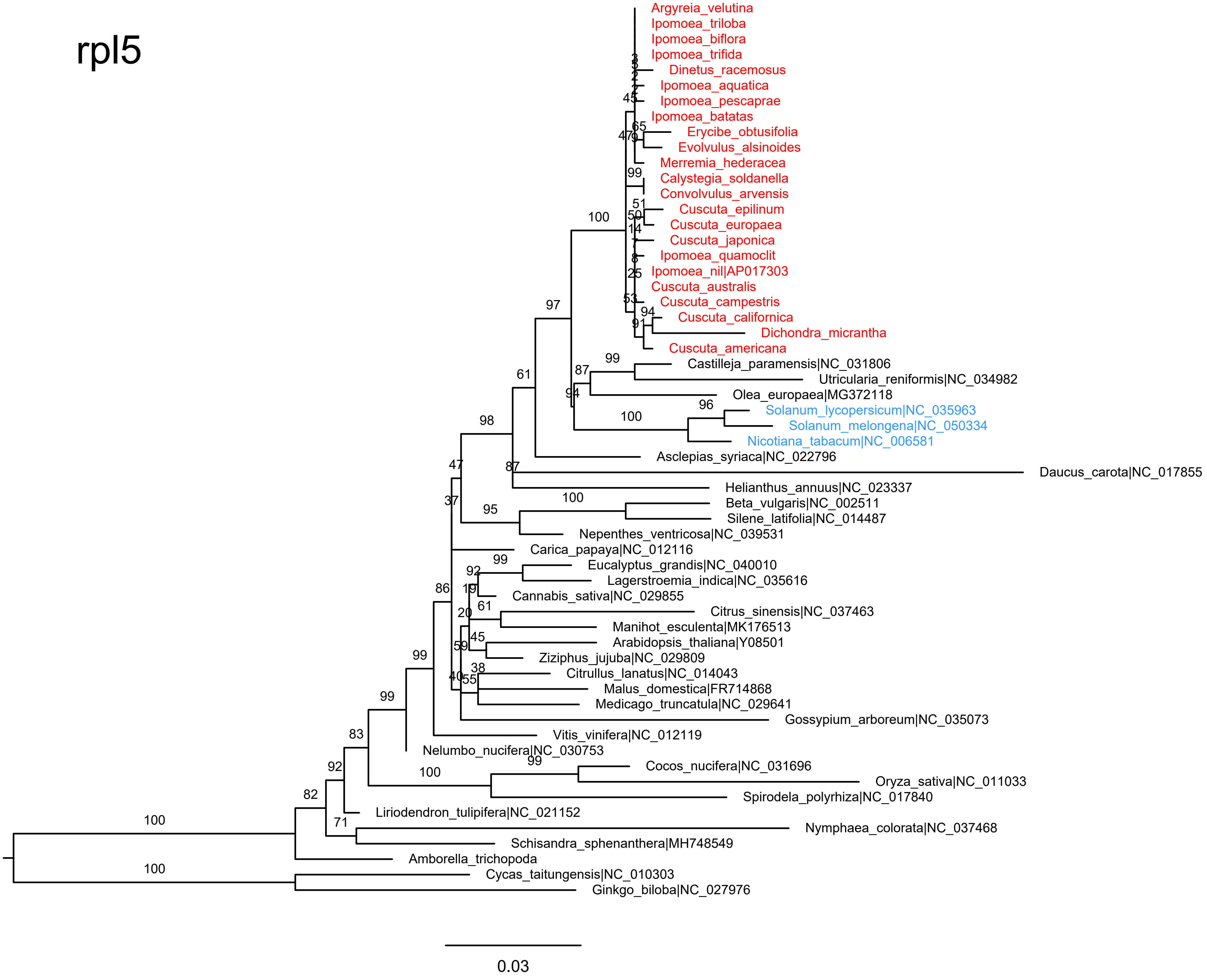
rpl5
Argyreia_velutina
Ipomoea_triloba
Ipomoea_biflora
Ipomoea_trifida
Dinetus_racemosus
Ipomoea_aquatica
Ipomoea_pescaprae
Ipomoea_batatas
Erycibe_obtusifolia
Evolvulus_alsinoides
Merremia_hederacea
Calystegia_soldanella
Convolvulus_arvensis
Cuscuta_epilinum
Cuscuta_europaea
Cuscuta_japonica
Ipomoea_quamoclit
Ipomoea_nil|AP017303
Cuscuta_australis
Cuscuta_campestris
Cuscuta_californica
Dichondra_micrantha
Cuscuta_americana
Castilleja_paramensis|NC_031806
Utricularia_reniformis|NC_034982
Olea_europaea|MG372118
Solanum_lycopersicum|NC_035963
Solanum_melongena|NC_050334
Nicotiana_tabacum|NC_006581
Asclepias_syriaca|NC_022796
Daucus_carota|NC_017855
Helianthus_annuus|NC_023337
Beta_vulgaris|NC_002511
Silene_latifolia|NC_014487
Nepenthes_ventricosa|NC_039531
Carica_papaya|NC_012116
Eucalyptus_grandis|NC_040010
Lagerstroemia_indica|NC_035616
Cannabis_sativa|NC_029855
Citrus_sinensis|NC_037463
Manihot_esculenta|MK176513
Arabidopsis_thaliana|Y08501
Ziziphus_jujuba|NC_029809
Citrullus_lanatus|NC_014043
Malus_domestica|FR714868
Medicago_truncatula|NC_029641
Gossypium_arboreum|NC_035073
Vitis_vinifera|NC_012119
Nelumbo_nucifera|NC_030753
Cocos_nucifera|NC_031696
Oryza_sativa|NC_011033
Spirodela_polyrhiza|NC_017840
Liriodendron_tulipifera|NC_021152
Nymphaea_colorata|NC_037468
Schisandra_sphenanthera|MH748549
Amborella_trichopoda
Cycas_taitungensis|NC_010303
Ginkgo_biloba|NC_027976
0.03

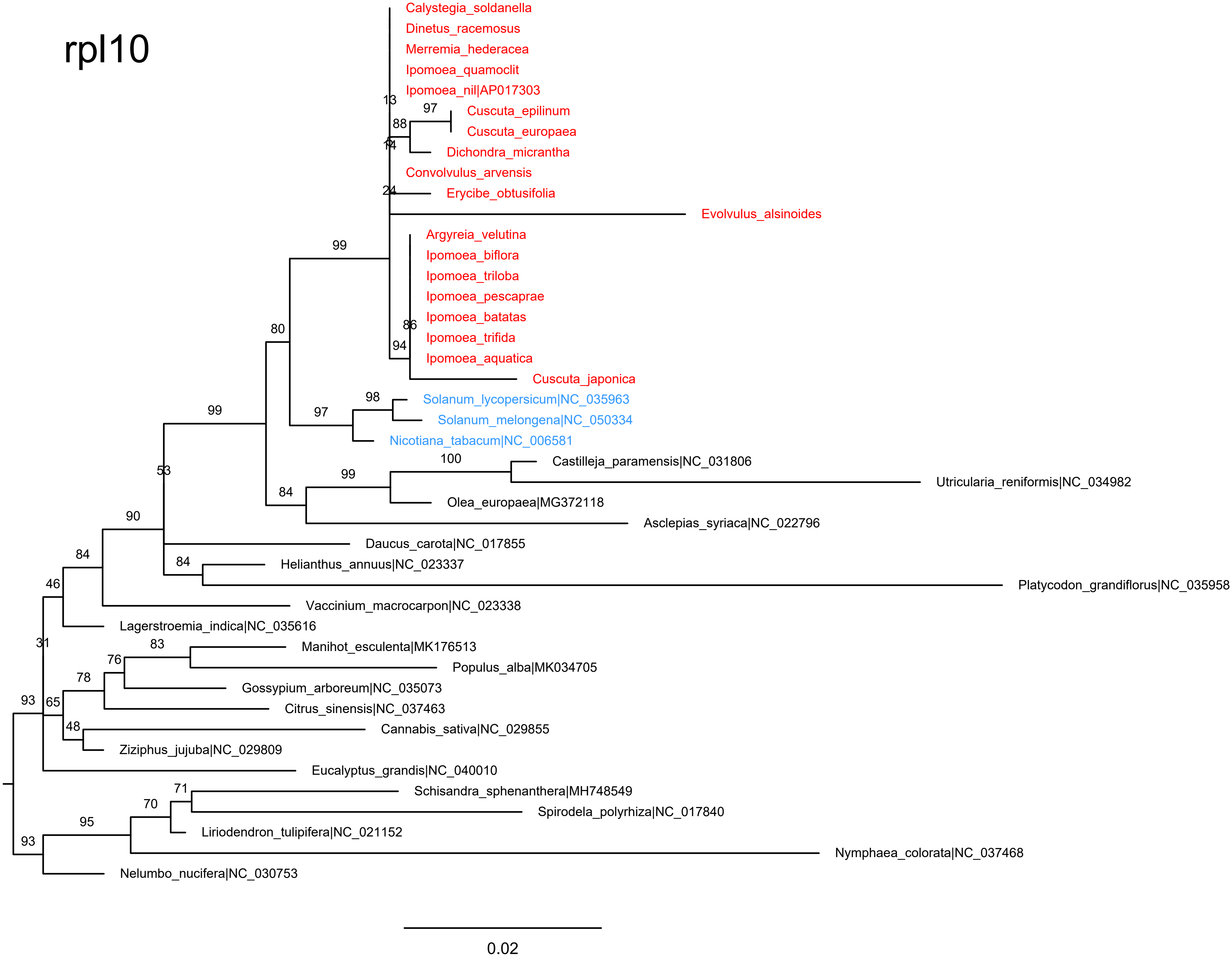

rpl10
Calystegia_soldanella
Dinetus_racemosus
Merremia_hederacea
Ipomoea_quamoclit
Ipomoea_nil|AP017303
Cuscuta_epilinum
Cuscuta_europaea
Dichondra_micrantha
Convolvulus_arvensis
Erycibe_obtusifolia
Evolvulus_alsinoides
Argyreia_velutina
Ipomoea_biflora
Ipomoea_triloba
Ipomoea_pescaprae
Ipomoea_batatas
Ipomoea_trifida
Ipomoea_aquatica
Cuscuta_japonica
Solanum_lycopersicum|NC_035963
Solanum_melongena|NC_050334
Nicotiana_tabacum|NC_006581
Castilleja_paramensis|NC_031806
Utricularia_reniformis|NC_034982
Olea_europaea|MG372118
Asclepias_syriaca|NC_022796
Daucus_carota|NC_017855
Helianthus_annuus|NC_023337
Platycodon_grandiflorus|NC_035958
Vaccinium_macrocarpon|NC_023338
Lagerstroemia_indica|NC_035616
Manihot_esculenta|MK176513
Populus_alba|MK034705
Gossypium_arboreum|NC_035073
Citrus_sinensis|NC_037463
Cannabis_sativa|NC_029855
Ziziphus_jujuba|NC_029809
Eucalyptus_grandis|NC_040010
Schisandra_sphenanthera|MH748549
Spirodela_polyrhiza|NC_017840
Liriodendron_tulipifera|NC_021152
Nymphaea_colorata|NC_037468
Nelumbo_nucifera|NC_030753
0.02

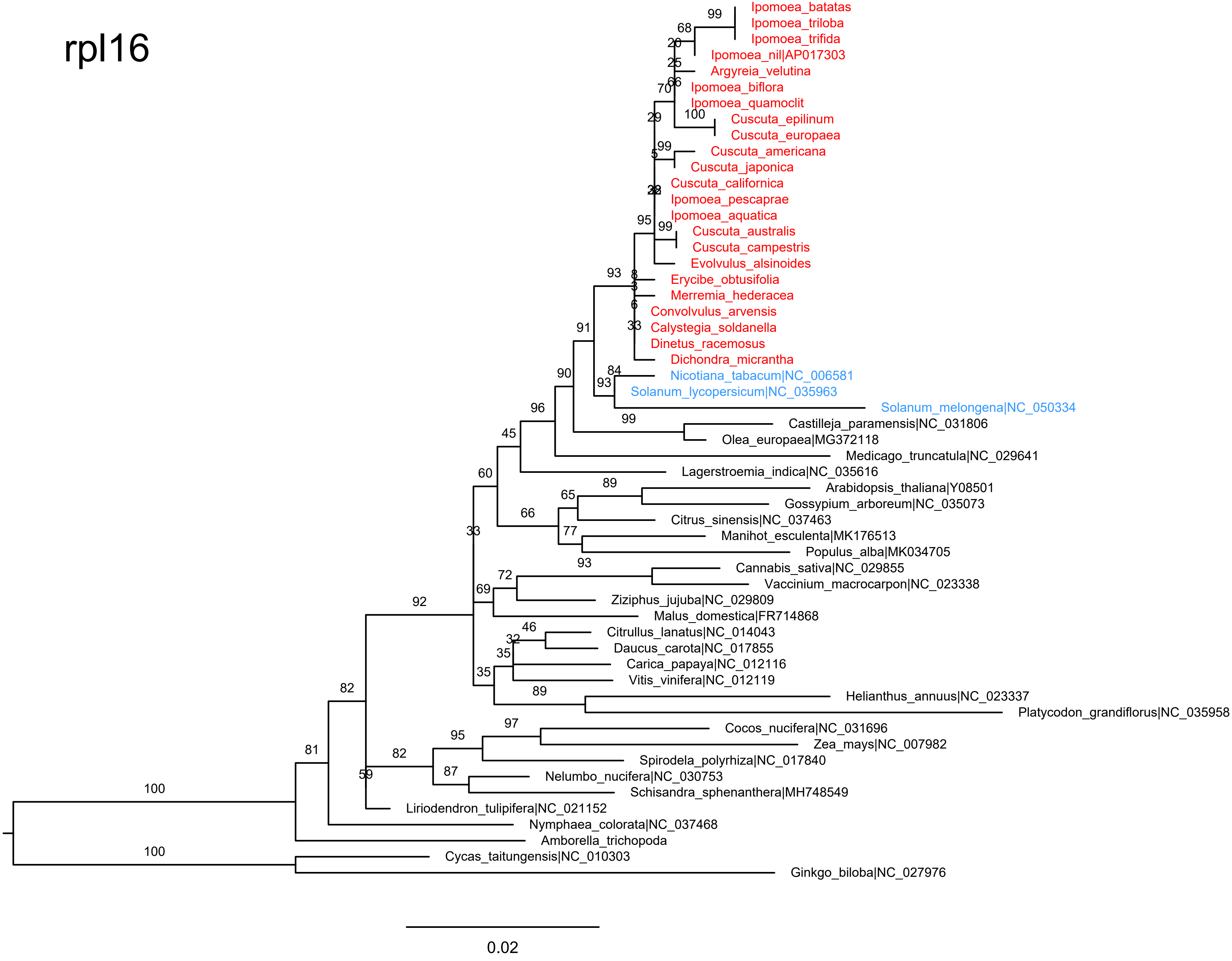
rpl16
Ipomoea_batatas
Ipomoea_triloba
Ipomoea_trifida
Ipomoea_nil|AP017303
Argyreia_velutina
Ipomoea_biflora
Ipomoea_quamoclit
Cuscuta_epilinum
Cuscuta_europaea
Cuscuta_americana
Cuscuta_japonica
Cuscuta_californica
Ipomoea_pescaprae
Ipomoea_aquatica
Cuscuta_australis
Cuscuta_campestris
Evolvulus_alsinoides
Erycibe_obtusifolia
Merremia_hederacea
Convolvulus_arvensis
Calystegia_soldanella
Dinetus_racemosus
Dichondra_micrantha
Nicotiana_tabacum|NC_006581
Solanum_lycopersicum|NC_035963
Solanum_melongena|NC_050334
Castilleja_paramensis|NC_031806
Olea_europaea|MG372118
Medicago_truncatula|NC_029641
Lagerstroemia_indica|NC_035616
Arabidopsis_thaliana|Y08501
Gossypium_arboreum|NC_035073
Citrus_sinensis|NC_037463
Manihot_esculenta|MK176513
Populus_alba|MK034705
Cannabis_sativa|NC_029855
Vaccinium_macrocarpon|NC_023338
Ziziphus_jujuba|NC_029809
Malus_domestica|FR714868
Citrullus_lanatus|NC_014043
Daucus_carota|NC_017855
Carica_papaya|NC_012116
Vitis_vinifera|NC_012119
Helianthus_annuus|NC_023337
Platycodon_grandiflorus|NC_035958
Cocos_nucifera|NC_031696
Zea_mays|NC_007982
Spirodela_polyrhiza|NC_017840
Nelumbo_nucifera|NC_030753
Schisandra_sphenanthera|MH748549
Liriodendron_tulipifera|NC_021152
Nymphaea_colorata|NC_037468
Amborella_trichopoda
Cycas_taitungensis|NC_010303
Ginkgo_biloba|NC_027976
0.02

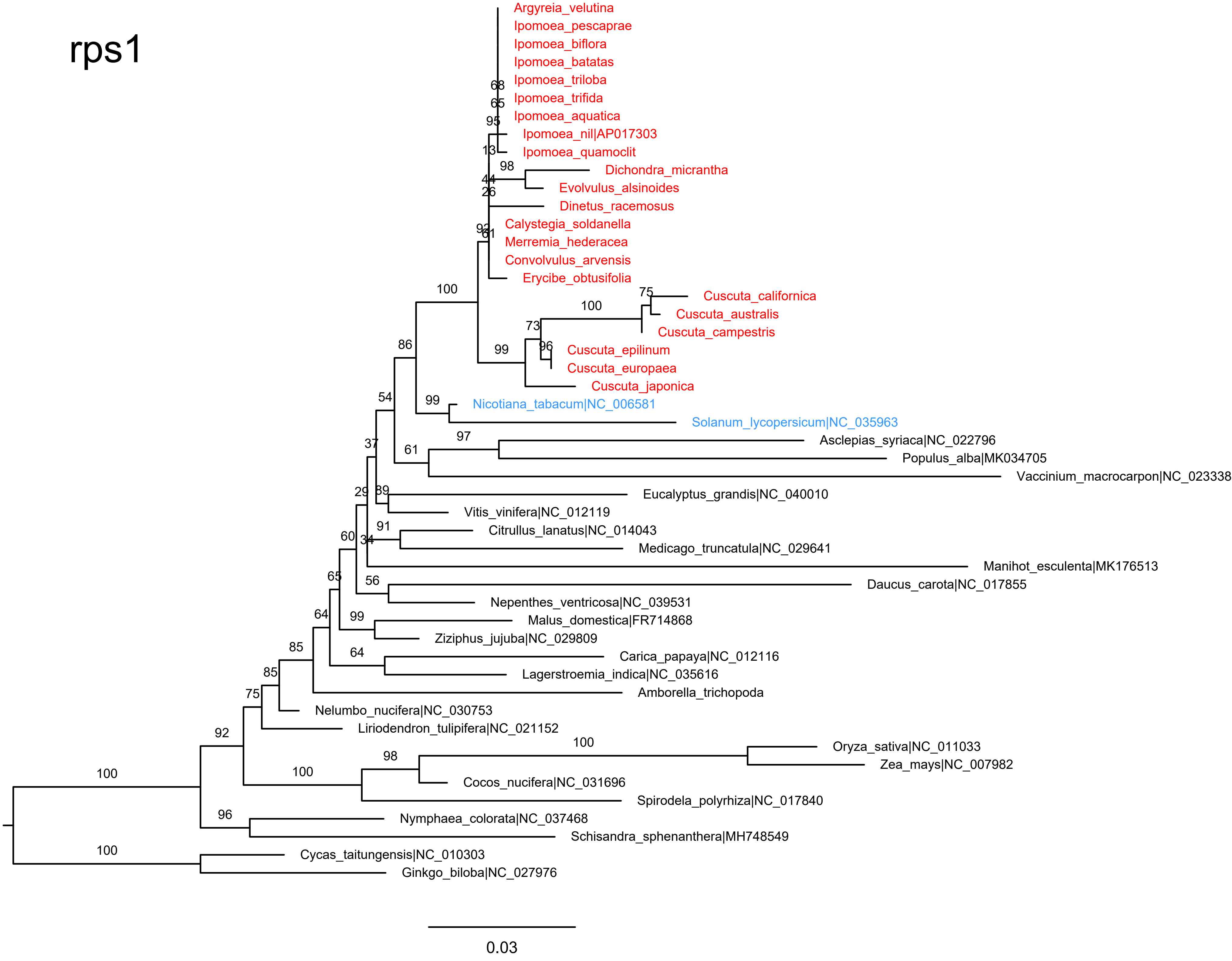

rps1
Argyreia_velutina
Ipomoea_pescaprae
Ipomoea_biflora
Ipomoea_batatas
Ipomoea_triloba
Ipomoea_trifida
Ipomoea_aquatica
Ipomoea_nil|AP017303
Ipomoea_quamoclit
Dichondra_micrantha
Evolvulus_alsinoides
Dinetus_racemosus
Calystegia_soldanella
Merremia_hederacea
Convolvulus_arvensis
Erycibe_obtusifolia
Cuscuta_californica
Cuscuta_australis
Cuscuta_campestris
Cuscuta_epilinum
Cuscuta_europaea
Cuscuta_japonica
Nicotiana_tabacum|NC_006581
Solanum_lycopersicum|NC_035963
Asclepias_syriaca|NC_022796
Populus_alba|MK034705
Vaccinium_macrocarpon|NC_023338
Eucalyptus_grandis|NC_040010
Vitis_vinifera|NC_012119
Citrullus_lanatus|NC_014043
Medicago_truncatula|NC_029641
Manihot_esculenta|MK176513
Daucus_carota|NC_017855
Nepenthes_ventricosa|NC_039531
Malus_domestica|FR714868
Ziziphus_jujuba|NC_029809
Carica_papaya|NC_012116
Lagerstroemia_indica|NC_035616
Amborella_trichopoda
Nelumbo_nucifera|NC_030753
Liriodendron_tulipifera|NC_021152
Oryza_sativa|NC_011033
Zea_mays|NC_007982
Cocos_nucifera|NC_031696
Spirodela_polyrhiza|NC_017840
Nymphaea_colorata|NC_037468
Schisandra_sphenanthera|MH748549
Cycas_taitungensis|NC_010303
Ginkgo_biloba|NC_027976
68
65
95
13
98
44
26
92
61
100
75
100
73
99
96
86
54
99
37
61
97
29
89
91
60
34
65
56
64
99
85
64
85
75
92
100
98
100
100
96
100
0.03

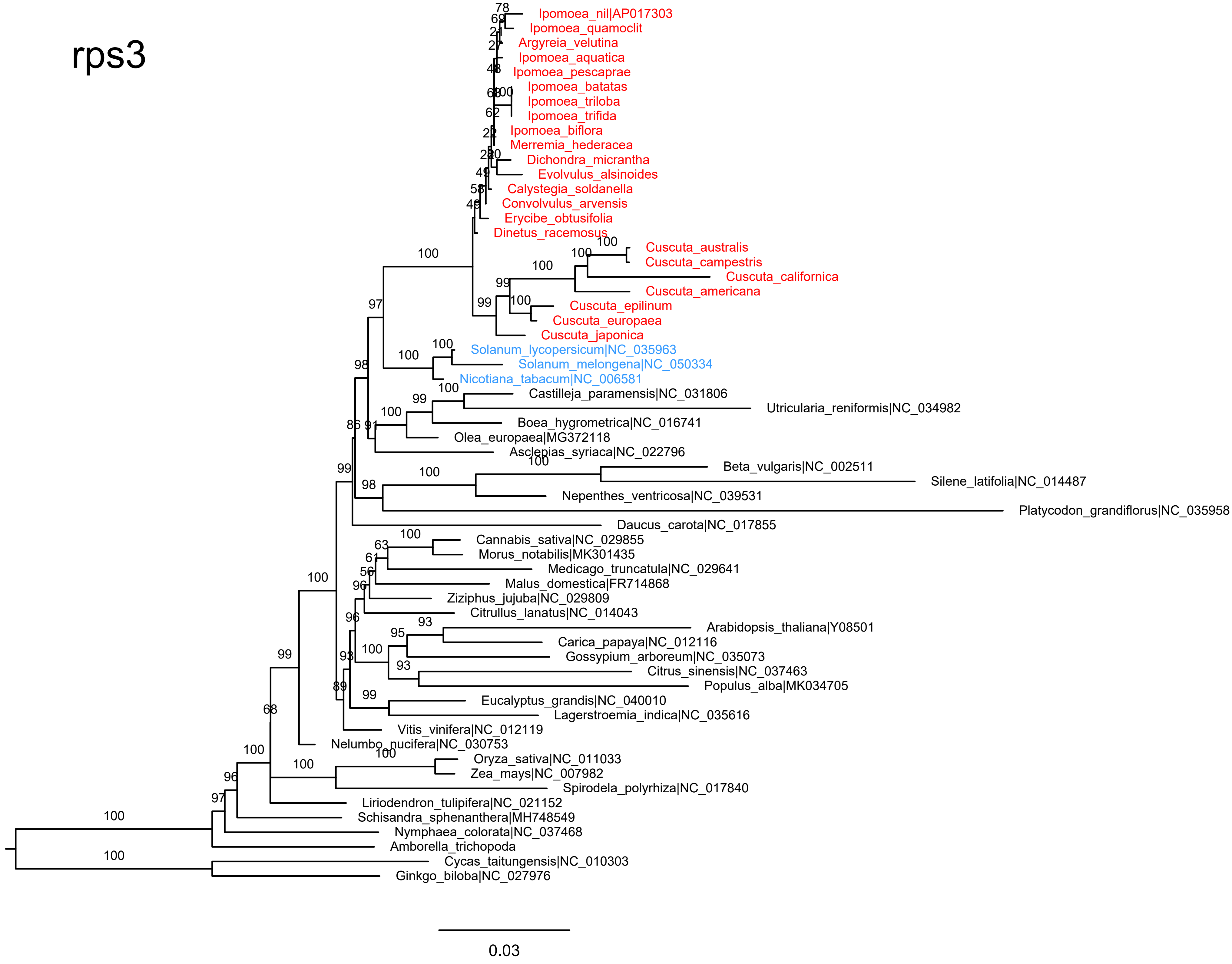
rps3
Ipomoea_nil|AP017303
Ipomoea_quamoclit
Argyreia_velutina
Ipomoea_aquatica
Ipomoea_pescaprae
Ipomoea_batatas
Ipomoea_triloba
Ipomoea_trifida
Ipomoea_biflora
Merremia_hederacea
Dichondra_micrantha
Evolvulus_alsinoides
Calystegia_soldanella
Convolvulus_arvensis
Erycibe_obtusifolia
Dinetus_racemosus
Cuscuta_australis
Cuscuta_campestris
Cuscuta_californica
Cuscuta_americana
Cuscuta_epilinum
Cuscuta_europaea
Cuscuta_japonica
Solanum_lycopersicum|NC_035963
Solanum_melongena|NC_050334
Nicotiana_tabacum|NC_006581
Castilleja_paramensis|NC_031806
Utricularia_reniformis|NC_034982
Boea_hygrometrica|NC_016741
Olea_europaea|MG372118
Asclepias_syriaca|NC_022796
Beta_vulgaris|NC_002511
Silene_latifolia|NC_014487
Nepenthes_ventricosa|NC_039531
Platycodon_grandiflorus|NC_035958
Daucus_carota|NC_017855
Cannabis_sativa|NC_029855
Morus_notabilis|MK301435
Medicago_truncatula|NC_029641
Malus_domestica|FR714868
Ziziphus_jujuba|NC_029809
Citrullus_lanatus|NC_014043
Arabidopsis_thaliana|Y08501
Carica_papaya|NC_012116
Gossypium_arboreum|NC_035073
Citrus_sinensis|NC_037463
Populus_alba|MK034705
Eucalyptus_grandis|NC_040010
Lagerstroemia_indica|NC_035616
Vitis_vinifera|NC_012119
Nelumbo_nucifera|NC_030753
Oryza_sativa|NC_011033
Zea_mays|NC_007982
Spirodela_polyrhiza|NC_017840
Liriodendron_tulipifera|NC_021152
Schisandra_sphenanthera|MH748549
Nymphaea_colorata|NC_037468
Amborella_trichopoda
Cycas_taitungensis|NC_010303
Ginkgo_biloba|NC_027976
0.03

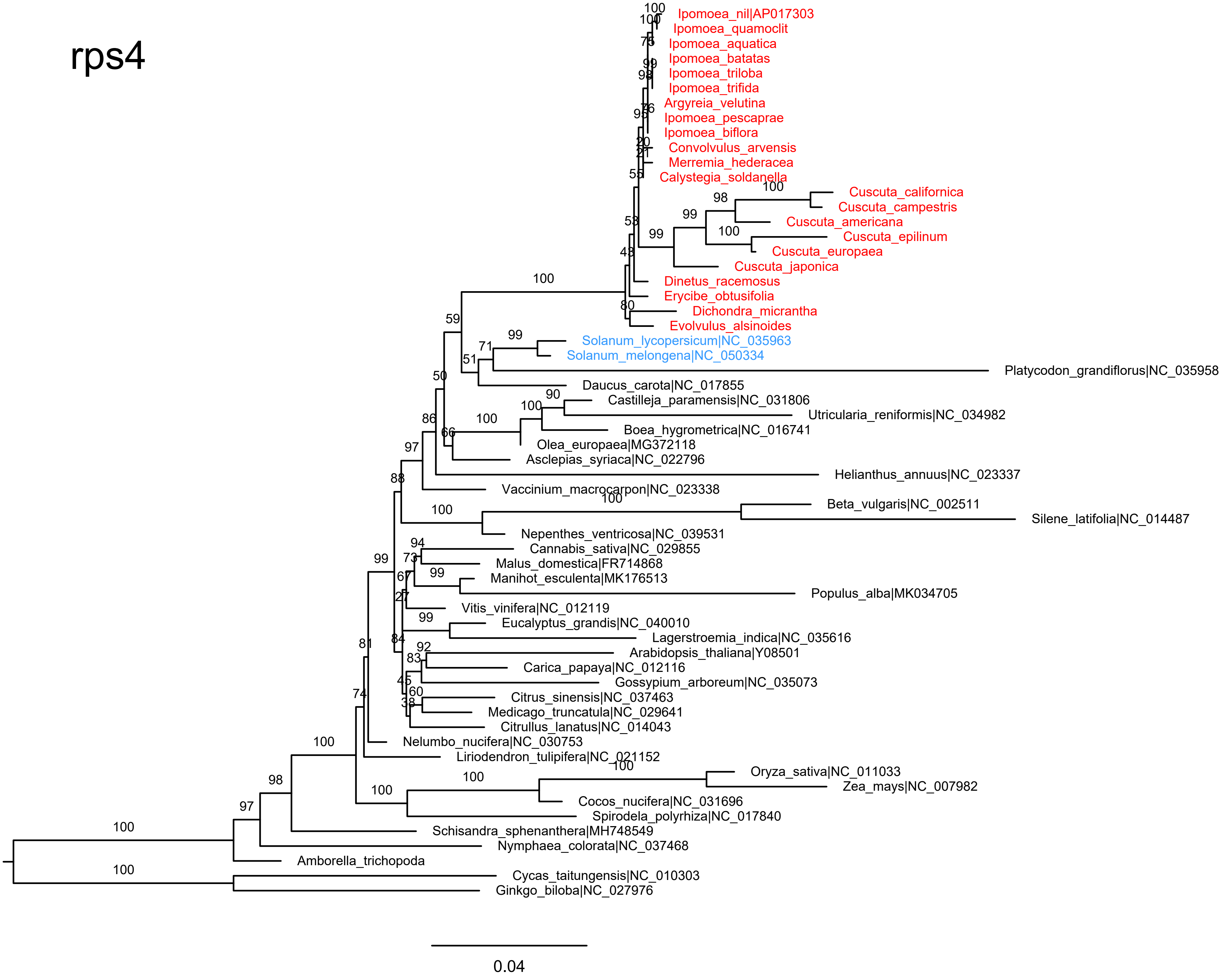

rps4
Ipomoea_nil|AP017303
Ipomoea_quamoclit
Ipomoea_aquatica
Ipomoea_batatas
Ipomoea_triloba
Ipomoea_trifida
Argyreia_velutina
Ipomoea_pescaprae
Ipomoea_biflora
Convolvulus_arvensis
Merremia_hederacea
Calystegia_soldanella
Cuscuta_californica
Cuscuta_campestris
Cuscuta_americana
Cuscuta_epilinum
Cuscuta_europaea
Cuscuta_japonica
Dinetus_racemosus
Erycibe_obtusifolia
Dichondra_micrantha
Evolvulus_alsinoides
Solanum_lycopersicum|NC_035963
Solanum_melongena|NC_050334
Platycodon_grandiflorus|NC_035958
Daucus_carota|NC_017855
Castilleja_paramensis|NC_031806
Utricularia_reniformis|NC_034982
Boea_hygrometrica|NC_016741
Olea_europaea|MG372118
Asclepias_syriaca|NC_022796
Helianthus_annuus|NC_023337
Vaccinium_macrocarpon|NC_023338
Beta_vulgaris|NC_002511
Silene_latifolia|NC_014487
Nepenthes_ventricosa|NC_039531
Cannabis_sativa|NC_029855
Malus_domestica|FR714868
Manihot_esculenta|MK176513
Populus_alba|MK034705
Vitis_vinifera|NC_012119
Eucalyptus_grandis|NC_040010
Lagerstroemia_indica|NC_035616
Arabidopsis_thaliana|Y08501
Carica_papaya|NC_012116
Gossypium_arboreum|NC_035073
Citrus_sinensis|NC_037463
Medicago_truncatula|NC_029641
Citrullus_lanatus|NC_014043
Nelumbo_nucifera|NC_030753
Liriodendron_tulipifera|NC_021152
Oryza_sativa|NC_011033
Zea_mays|NC_007982
Cocos_nucifera|NC_031696
Spirodela_polyrhiza|NC_017840
Schisandra_sphenanthera|MH748549
Nymphaea_colorata|NC_037468
Amborella_trichopoda
Cycas_taitungensis|NC_010303
Ginkgo_biloba|NC_027976
0.04

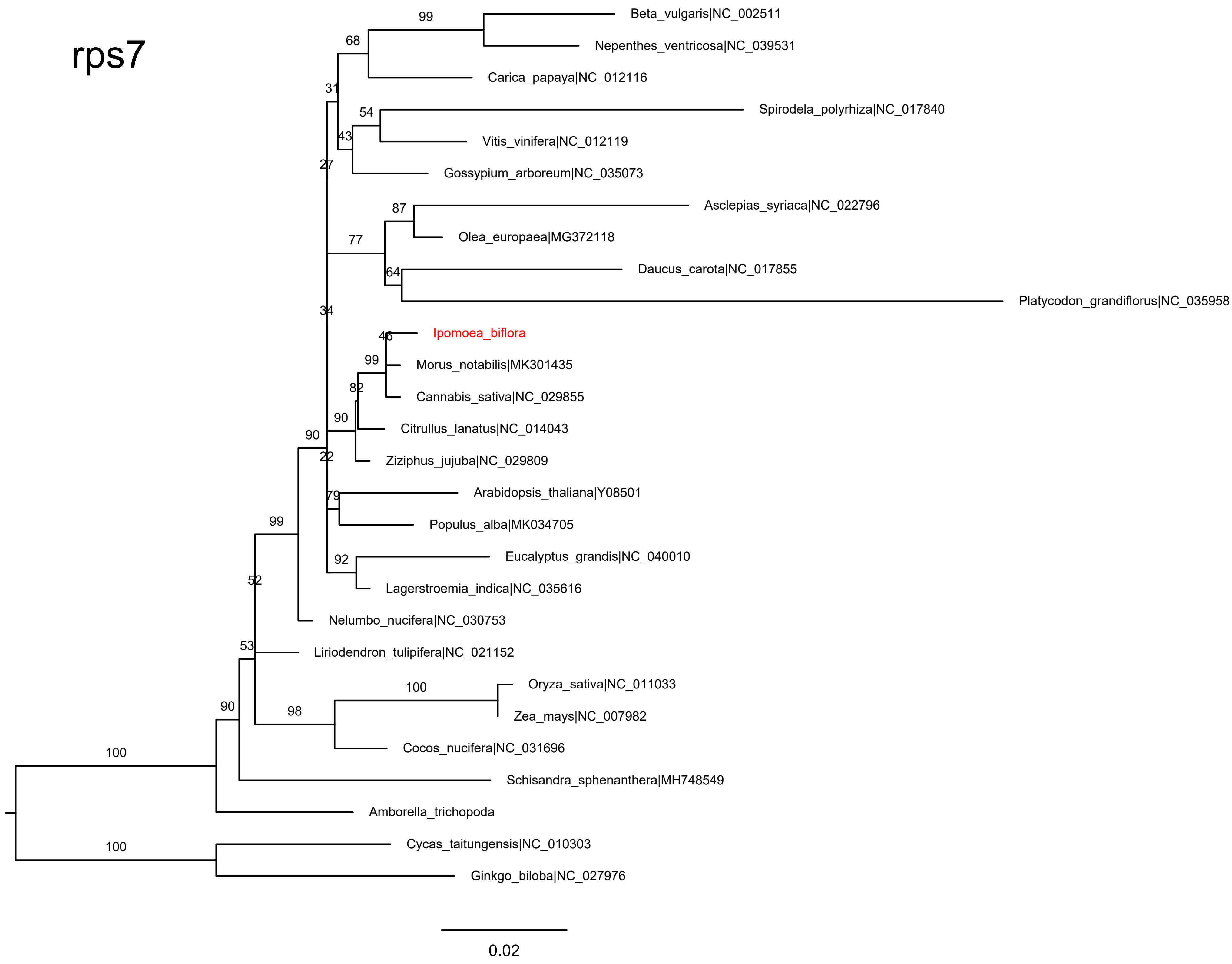

rps7
Beta_vulgaris|NC_002511
Nepenthes_ventricosa|NC_039531
Carica_papaya|NC_012116
Spirodela_polyrhiza|NC_017840
Vitis_vinifera|NC_012119
Gossypium_arboreum|NC_035073
Asclepias_syriaca|NC_022796
Olea_europaea|MG372118
Daucus_carota|NC_017855
Platycodon_grandiflorus|NC_035958
Ipomoea_biflora
Morus_notabilis|MK301435
Cannabis_sativa|NC_029855
Citrullus_lanatus|NC_014043
Ziziphus_jujuba|NC_029809
Arabidopsis_thaliana|Y08501
Populus_alba|MK034705
Eucalyptus_grandis|NC_040010
Lagerstroemia_indica|NC_035616
Nelumbo_nucifera|NC_030753
Liriodendron_tulipifera|NC_021152
Oryza_sativa|NC_011033
Zea_mays|NC_007982
Cocos_nucifera|NC_031696
Schisandra_sphenanthera|MH748549
Amborella_trichopoda
Cycas_taitungensis|NC_010303
Ginkgo_biloba|NC_027976
99
68
31
54
43
27
87
77
64
34
46
99
82
90
90
22
79
99
92
52
53
100
90
98
100
100
0.02

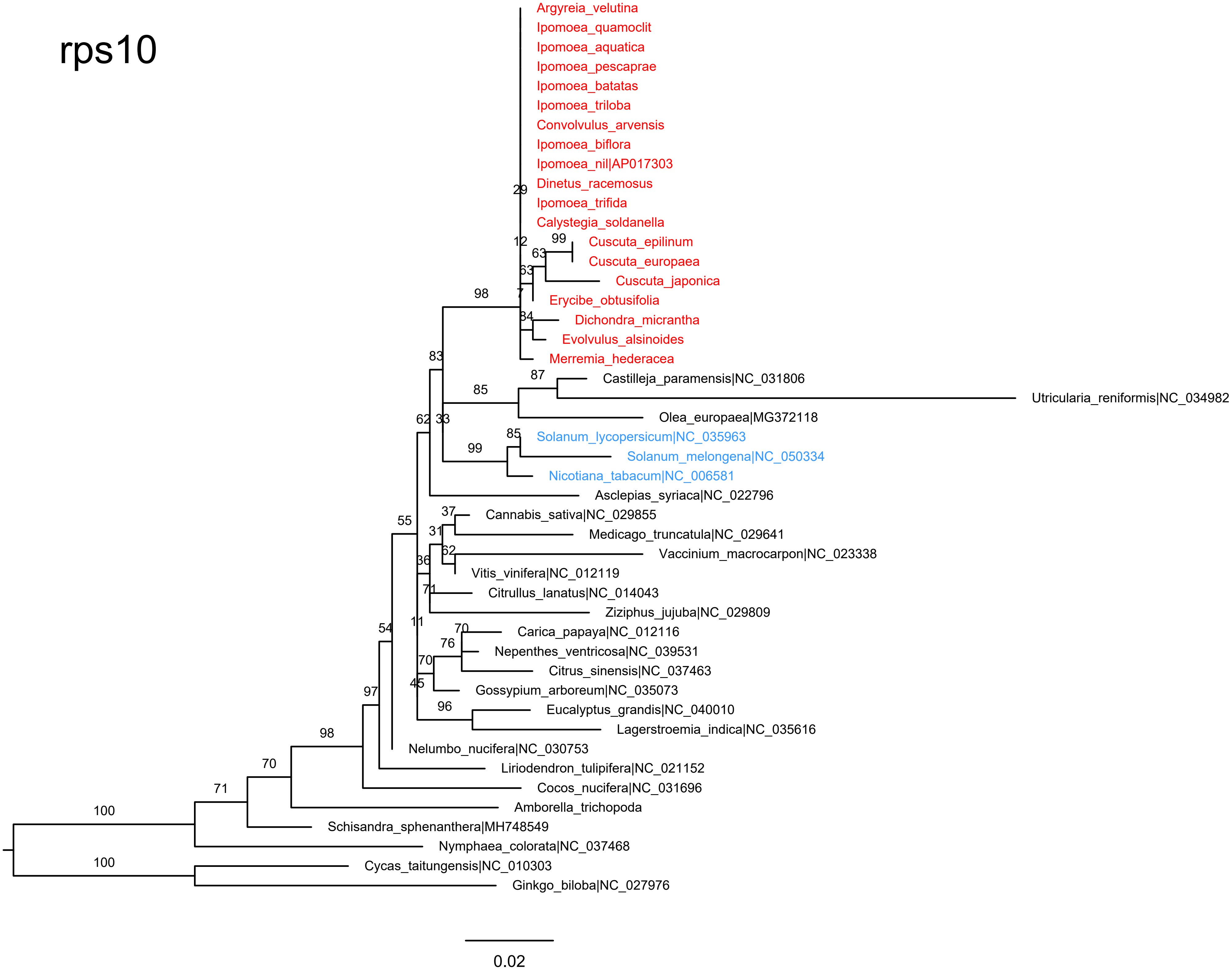
rps10
Argyreia_velutina
Ipomoea_quamoclit
Ipomoea_aquatica
Ipomoea_pescaprae
Ipomoea_batatas
Ipomoea_triloba
Convolvulus_arvensis
Ipomoea_biflora
Ipomoea_nil|AP017303
Dinetus_racemosus
Ipomoea_trifida
Calystegia_soldanella
Cuscuta_epilinum
Cuscuta_europaea
Cuscuta_japonica
Erycibe_obtusifolia
Dichondra_micrantha
Evolvulus_alsinoides
Merremia_hederacea
Castilleja_paramensis|NC_031806
Utricularia_reniformis|NC_034982
Olea_europaea|MG372118
Solanum_lycopersicum|NC_035963
Solanum_melongena|NC_050334
Nicotiana_tabacum|NC_006581
Asclepias_syriaca|NC_022796
Cannabis_sativa|NC_029855
Medicago_truncatula|NC_029641
Vaccinium_macrocarpon|NC_023338
Vitis_vinifera|NC_012119
Citrullus_lanatus|NC_014043
Ziziphus_jujuba|NC_029809
Carica_papaya|NC_012116
Nepenthes_ventricosa|NC_039531
Citrus_sinensis|NC_037463
Gossypium_arboreum|NC_035073
Eucalyptus_grandis|NC_040010
Lagerstroemia_indica|NC_035616
Nelumbo_nucifera|NC_030753
Liriodendron_tulipifera|NC_021152
Cocos_nucifera|NC_031696
Amborella_trichopoda
Schisandra_sphenanthera|MH748549
Nymphaea_colorata|NC_037468
Cycas_taitungensis|NC_010303
Ginkgo_biloba|NC_027976
0.02

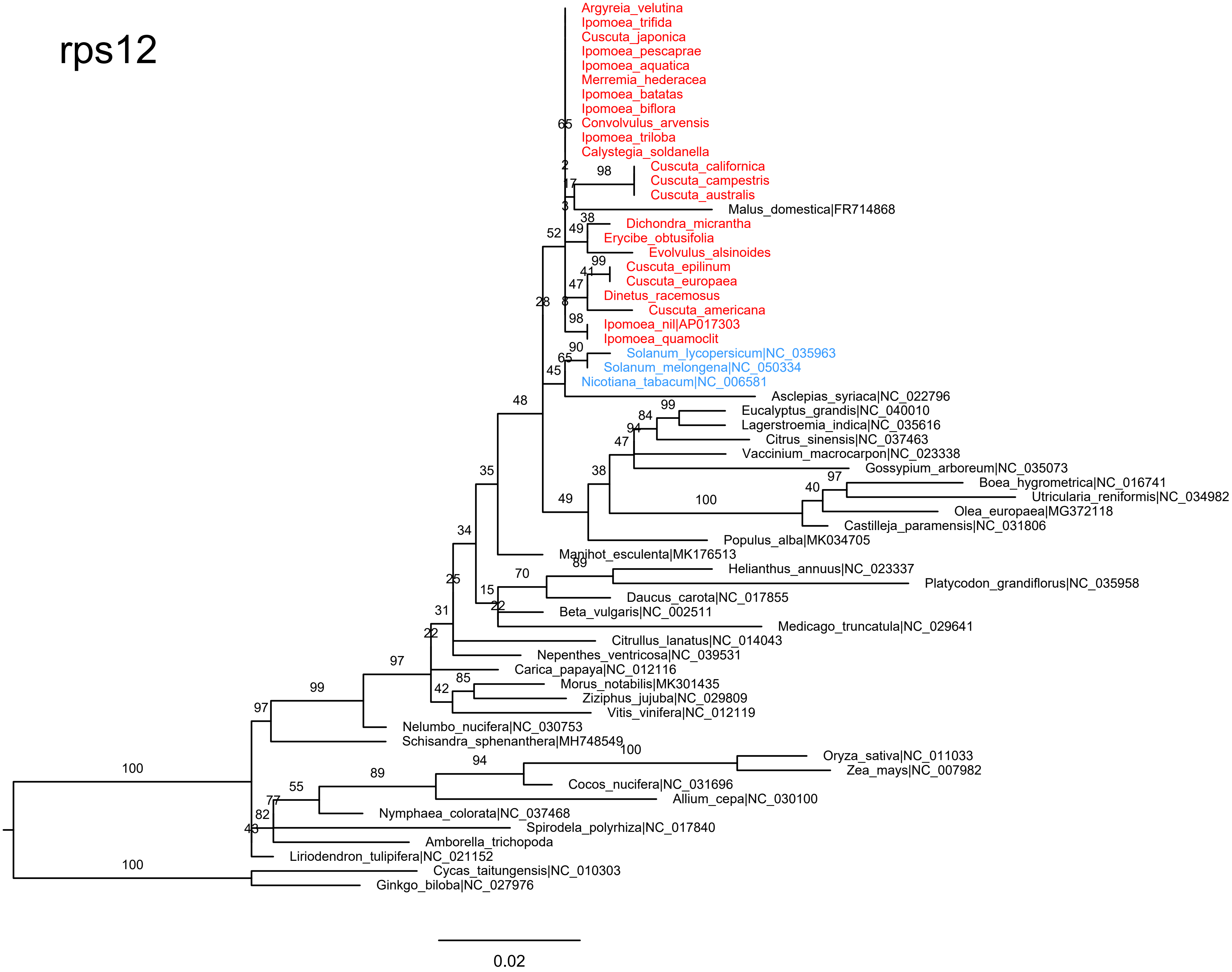

rps12
Argyreia_velutina
Ipomoea_trifida
Cuscuta_japonica
Ipomoea_pescaprae
Ipomoea_aquatica
Merremia_hederacea
Ipomoea_batatas
Ipomoea_biflora
Convolvulus_arvensis
Ipomoea_triloba
Calystegia_soldanella
Cuscuta_californica
Cuscuta_campestris
Cuscuta_australis
Malus_domestica|FR714868
Dichondra_micrantha
Erycibe_obtusifolia
Evolvulus_alsinoides
Cuscuta_epilinum
Cuscuta_europaea
Dinetus_racemosus
Cuscuta_americana
Ipomoea_nil|AP017303
Ipomoea_quamoclit
Solanum_lycopersicum|NC_035963
Solanum_melongena|NC_050334
Nicotiana_tabacum|NC_006581
Asclepias_syriaca|NC_022796
Eucalyptus_grandis|NC_040010
Lagerstroemia_indica|NC_035616
Citrus_sinensis|NC_037463
Vaccinium_macrocarpon|NC_023338
Gossypium_arboreum|NC_035073
Boea_hygrometrica|NC_016741
Utricularia_reniformis|NC_034982
Olea_europaea|MG372118
Castilleja_paramensis|NC_031806
Populus_alba|MK034705
Manihot_esculenta|MK176513
Helianthus_annuus|NC_023337
Platycodon_grandiflorus|NC_035958
Daucus_carota|NC_017855
Beta_vulgaris|NC_002511
Medicago_truncatula|NC_029641
Citrullus_lanatus|NC_014043
Nepenthes_ventricosa|NC_039531
Carica_papaya|NC_012116
Morus_notabilis|MK301435
Ziziphus_jujuba|NC_029809
Vitis_vinifera|NC_012119
Nelumbo_nucifera|NC_030753
Schisandra_sphenanthera|MH748549
Oryza_sativa|NC_011033
Zea_mays|NC_007982
Cocos_nucifera|NC_031696
Allium_cepa|NC_030100
Nymphaea_colorata|NC_037468
Spirodela_polyrhiza|NC_017840
Amborella_trichopoda
Liriodendron_tulipifera|NC_021152
Cycas_taitungensis|NC_010303
Ginkgo_biloba|NC_027976
0.02

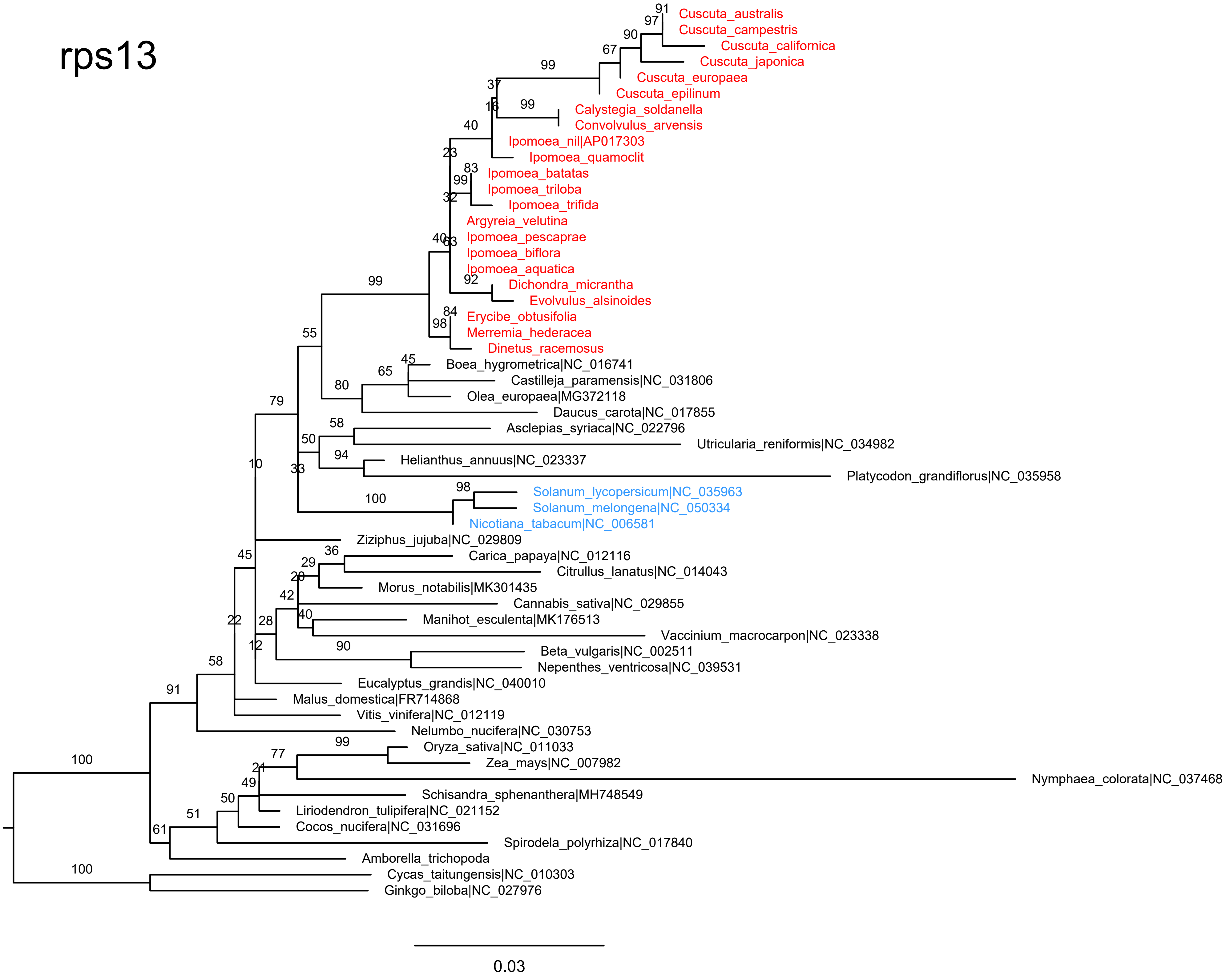
rps13
Cuscuta_australis
Cuscuta_campestris
Cuscuta_californica
Cuscuta_japonica
Cuscuta_europaea
Cuscuta_epilinum
Calystegia_soldanella
Convolvulus_arvensis
Ipomoea_nil|AP017303
Ipomoea_quamoclit
Ipomoea_batatas
Ipomoea_triloba
Ipomoea_trifida
Argyreia_velutina
Ipomoea_pescaprae
Ipomoea_biflora
Ipomoea_aquatica
Dichondra_micrantha
Evolvulus_alsinoides
Erycibe_obtusifolia
Merremia_hederacea
Dinetus_racemosus
Boea_hygrometrica|NC_016741
Castilleja_paramensis|NC_031806
Olea_europaea|MG372118
Daucus_carota|NC_017855
Asclepias_syriaca|NC_022796
Utricularia_reniformis|NC_034982
Helianthus_annuus|NC_023337
Platycodon_grandiflorus|NC_035958
Solanum_lycopersicum|NC_035963
Solanum_melongena|NC_050334
Nicotiana_tabacum|NC_006581
Ziziphus_jujuba|NC_029809
Carica_papaya|NC_012116
Citrullus_lanatus|NC_014043
Morus_notabilis|MK301435
Cannabis_sativa|NC_029855
Manihot_esculenta|MK176513
Vaccinium_macrocarpon|NC_023338
Beta_vulgaris|NC_002511
Nepenthes_ventricosa|NC_039531
Eucalyptus_grandis|NC_040010
Malus_domestica|FR714868
Vitis_vinifera|NC_012119
Nelumbo_nucifera|NC_030753
Oryza_sativa|NC_011033
Zea_mays|NC_007982
Nymphaea_colorata|NC_037468
Schisandra_sphenanthera|MH748549
Liriodendron_tulipifera|NC_021152
Cocos_nucifera|NC_031696
Spirodela_polyrhiza|NC_017840
Amborella_trichopoda
Cycas_taitungensis|NC_010303
Ginkgo_biloba|NC_027976
0.03

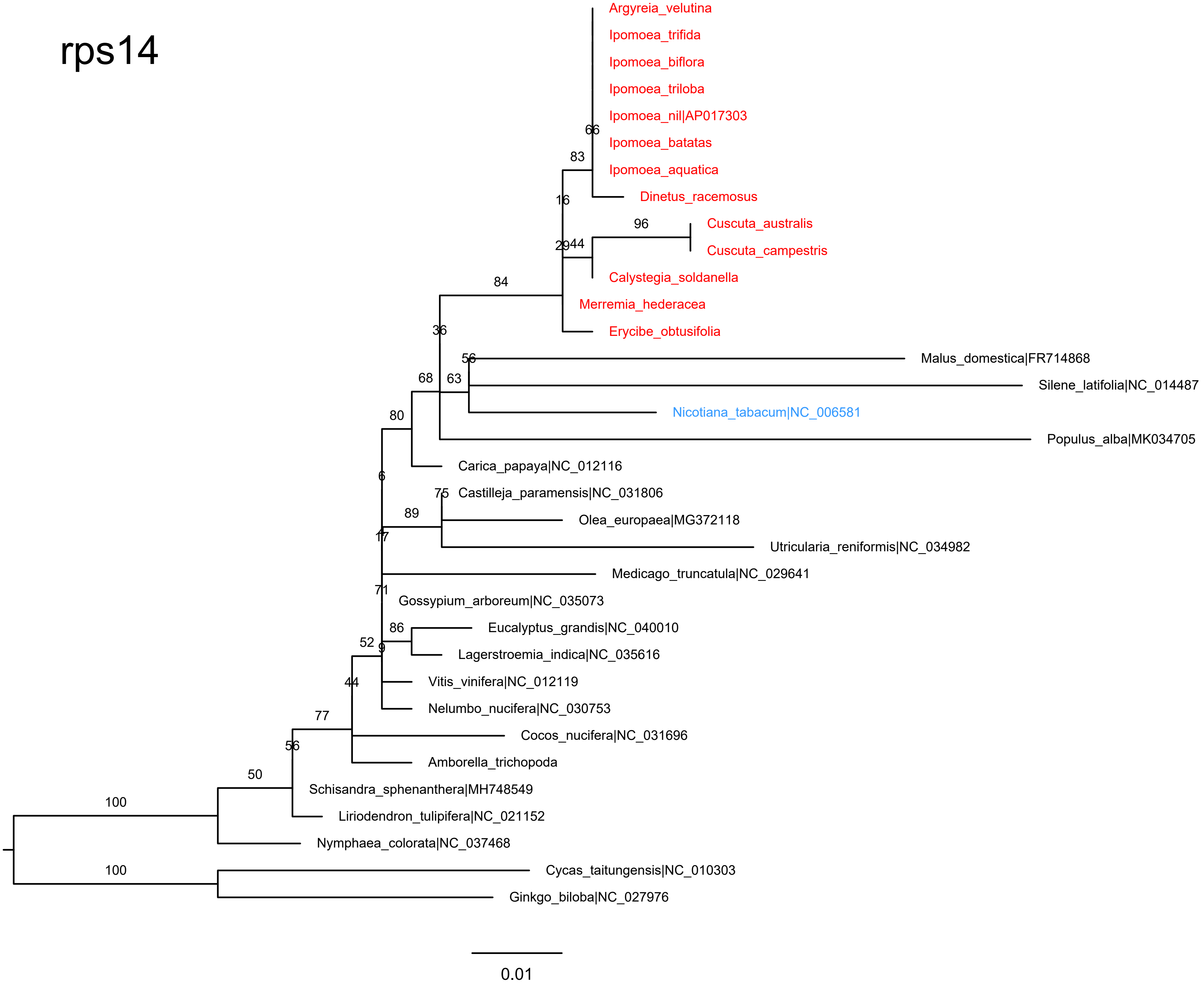
rps14
Argyreia_velutina
Ipomoea_trifida
Ipomoea_biflora
Ipomoea_triloba
Ipomoea_nil|AP017303
Ipomoea_batatas
Ipomoea_aquatica
Dinetus_racemosus
Cuscuta_australis
Cuscuta_campestris
Calystegia_soldanella
Merremia_hederacea
Erycibe_obtusifolia
Malus_domestica|FR714868
Silene_latifolia|NC_014487
Nicotiana_tabacum|NC_006581
Populus_alba|MK034705
Carica_papaya|NC_012116
Castilleja_paramensis|NC_031806
Olea_europaea|MG372118
Utricularia_reniformis|NC_034982
Medicago_truncatula|NC_029641
Gossypium_arboreum|NC_035073
Eucalyptus_grandis|NC_040010
Lagerstroemia_indica|NC_035616
Vitis_vinifera|NC_012119
Nelumbo_nucifera|NC_030753
Cocos_nucifera|NC_031696
Amborella_trichopoda
Schisandra_sphenanthera|MH748549
Liriodendron_tulipifera|NC_021152
Nymphaea_colorata|NC_037468
Cycas_taitungensis|NC_010303
Ginkgo_biloba|NC_027976
66
83
16
96
29
44
84
36
56
68
63
80
6
75
89
17
71
86
52
9
44
77
56
50
100
100
0.01

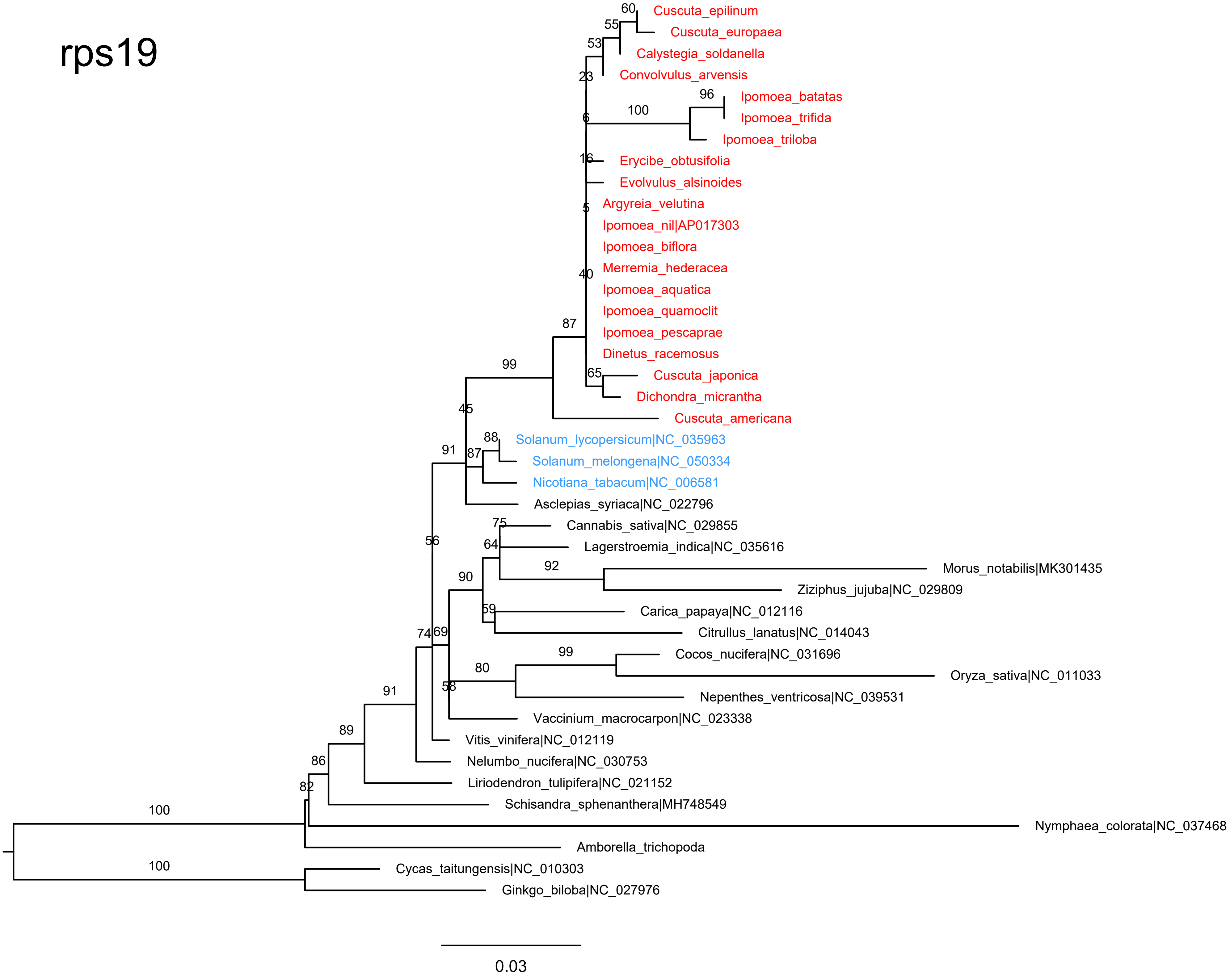

rps19
Cuscuta_epilinum
Cuscuta_europaea
Calystegia_soldanella
Convolvulus_arvensis
Ipomoea_batatas
Ipomoea_trifida
Ipomoea_triloba
Erycibe_obtusifolia
Evolvulus_alsinoides
Argyreia_velutina
Ipomoea_nil|AP017303
Ipomoea_biflora
Merremia_hederacea
Ipomoea_aquatica
Ipomoea_quamoclit
Ipomoea_pescaprae
Dinetus_racemosus
Cuscuta_japonica
Dichondra_micrantha
Cuscuta_americana
Solanum_lycopersicum|NC_035963
Solanum_melongena|NC_050334
Nicotiana_tabacum|NC_006581
Asclepias_syriaca|NC_022796
Cannabis_sativa|NC_029855
Lagerstroemia_indica|NC_035616
Morus_notabilis|MK301435
Ziziphus_jujuba|NC_029809
Carica_papaya|NC_012116
Citrullus_lanatus|NC_014043
Cocos_nucifera|NC_031696
Oryza_sativa|NC_011033
Nepenthes_ventricosa|NC_039531
Vaccinium_macrocarpon|NC_023338
Vitis_vinifera|NC_012119
Nelumbo_nucifera|NC_030753
Liriodendron_tulipifera|NC_021152
Schisandra_sphenanthera|MH748549
Nymphaea_colorata|NC_037468
Amborella_trichopoda
Cycas_taitungensis|NC_010303
Ginkgo_biloba|NC_027976
0.03

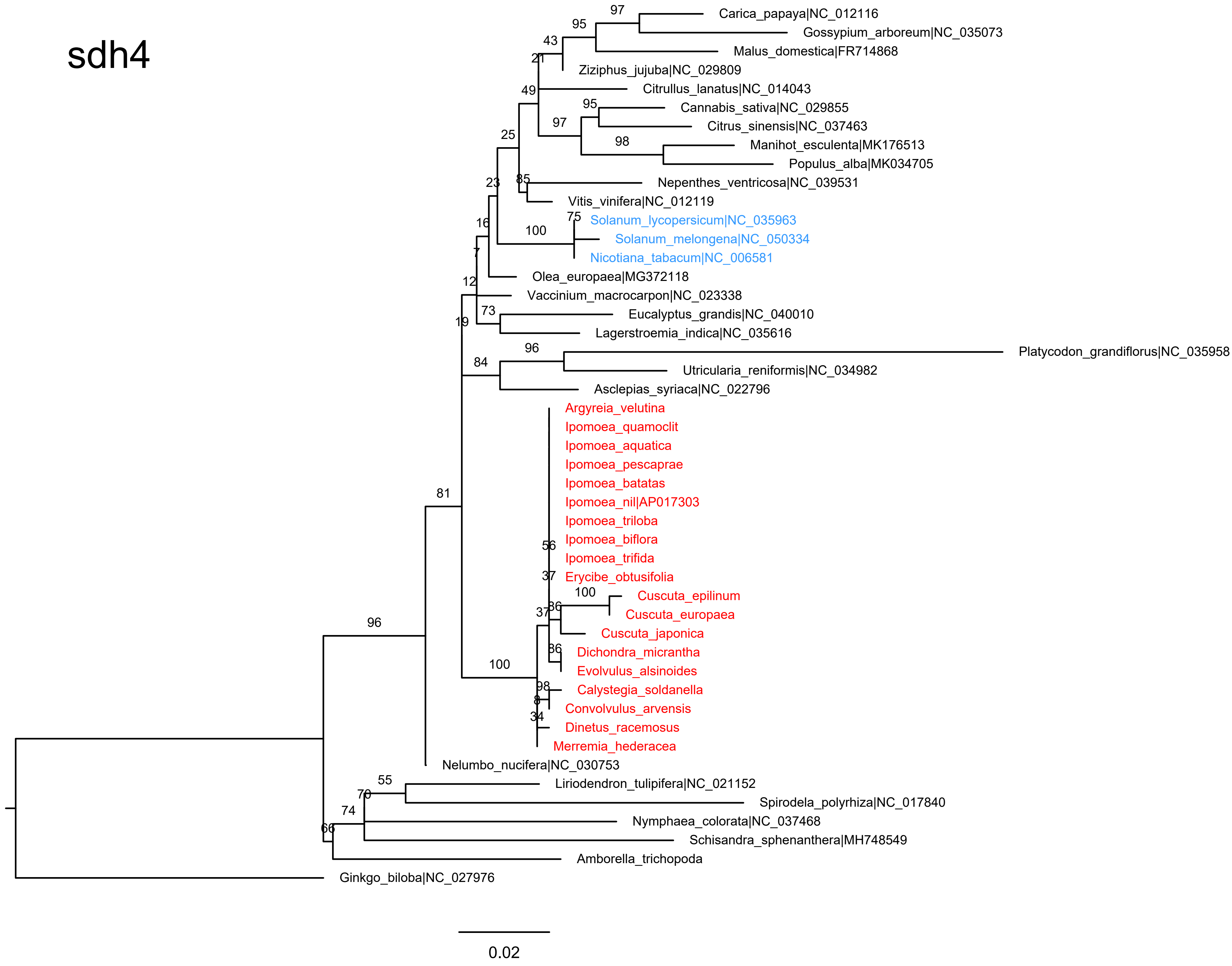
sdh4
Carica_papaya|NC_012116
Gossypium_arboreum|NC_035073
Malus_domestica|FR714868
Ziziphus_jujuba|NC_029809
Citrullus_lanatus|NC_014043
Cannabis_sativa|NC_029855
Citrus_sinensis|NC_037463
Manihot_esculenta|MK176513
Populus_alba|MK034705
Nepenthes_ventricosa|NC_039531
Vitis_vinifera|NC_012119
Solanum_lycopersicum|NC_035963
Solanum_melongena|NC_050334
Nicotiana_tabacum|NC_006581
Olea_europaea|MG372118
Vaccinium_macrocarpon|NC_023338
Eucalyptus_grandis|NC_040010
Lagerstroemia_indica|NC_035616
Platycodon_grandiflorus|NC_035958
Utricularia_reniformis|NC_034982
Asclepias_syriaca|NC_022796
Argyreia_velutina
Ipomoea_quamoclit
Ipomoea_aquatica
Ipomoea_pescaprae
Ipomoea_batatas
Ipomoea_nil|AP017303
Ipomoea_triloba
Ipomoea_biflora
Ipomoea_trifida
Erycibe_obtusifolia
Cuscuta_epilinum
Cuscuta_europaea
Cuscuta_japonica
Dichondra_micrantha
Evolvulus_alsinoides
Calystegia_soldanella
Convolvulus_arvensis
Dinetus_racemosus
Merremia_hederacea
Nelumbo_nucifera|NC_030753
Liriodendron_tulipifera|NC_021152
Spirodela_polyrhiza|NC_017840
Nymphaea_colorata|NC_037468
Schisandra_sphenanthera|MH748549
Amborella_trichopoda
Ginkgo_biloba|NC_027976
0.02
