# Supplementary figures and images for: Unprecedented organelle genomic variations in morning glories reveal independent evolutionary scenarios of parasitic plants and the diversification of plant mitochondrial complexes
Source: BMC Biol. 2022 Feb 16;20:49. doi: 10.1186/s12915-022-01250-1 (PMC8851834; doi:10.1186/s12915-022-01250-1)

Plastid CDS

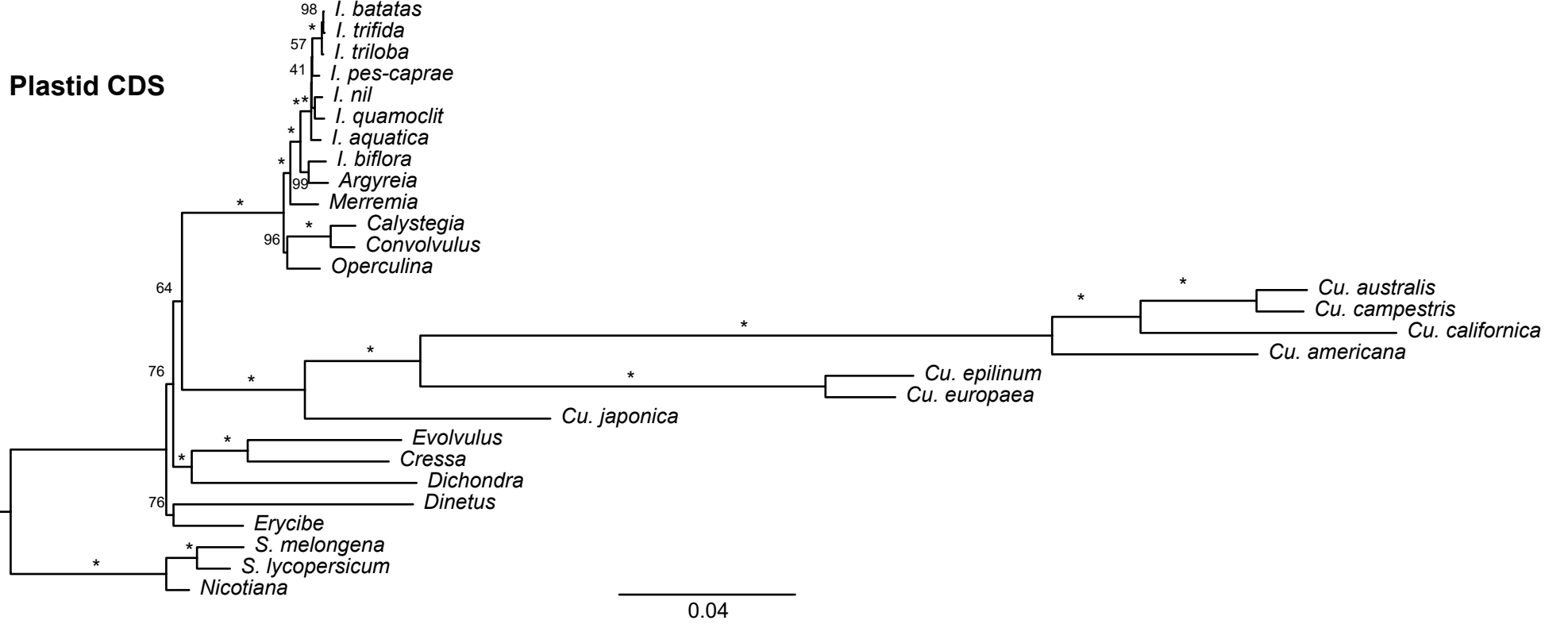

Mitochondrial CDS

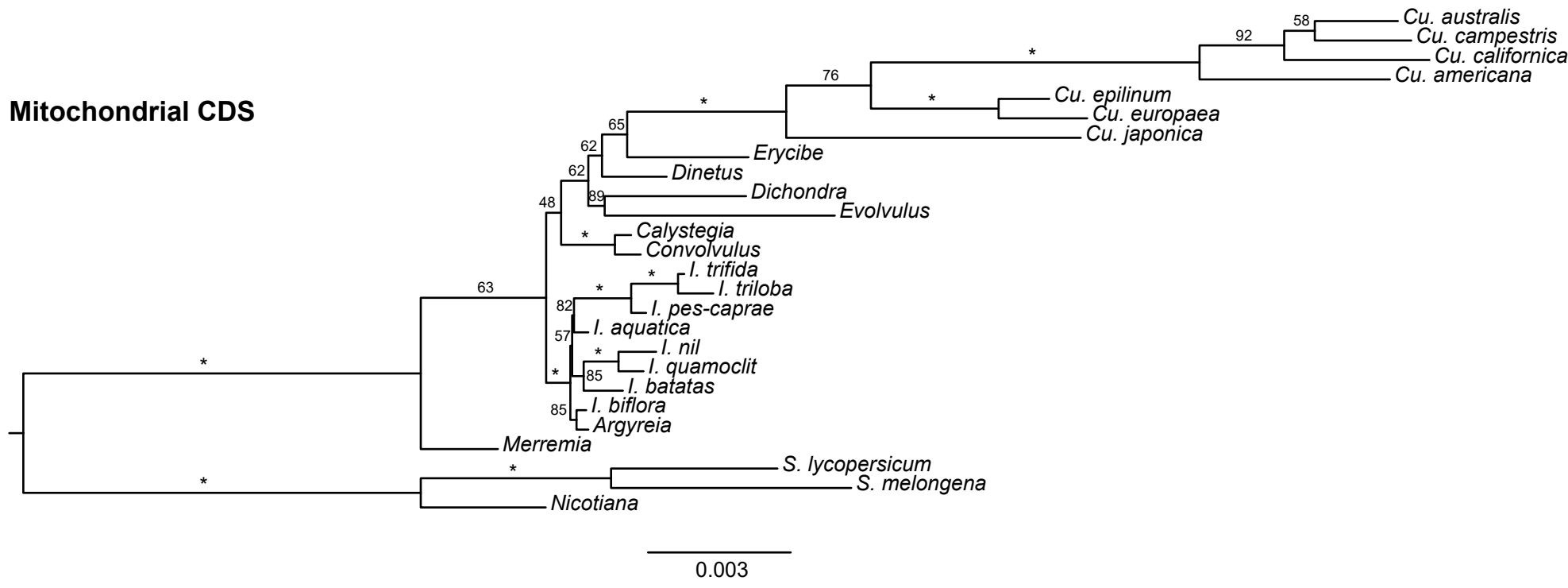

Nuclear 45S

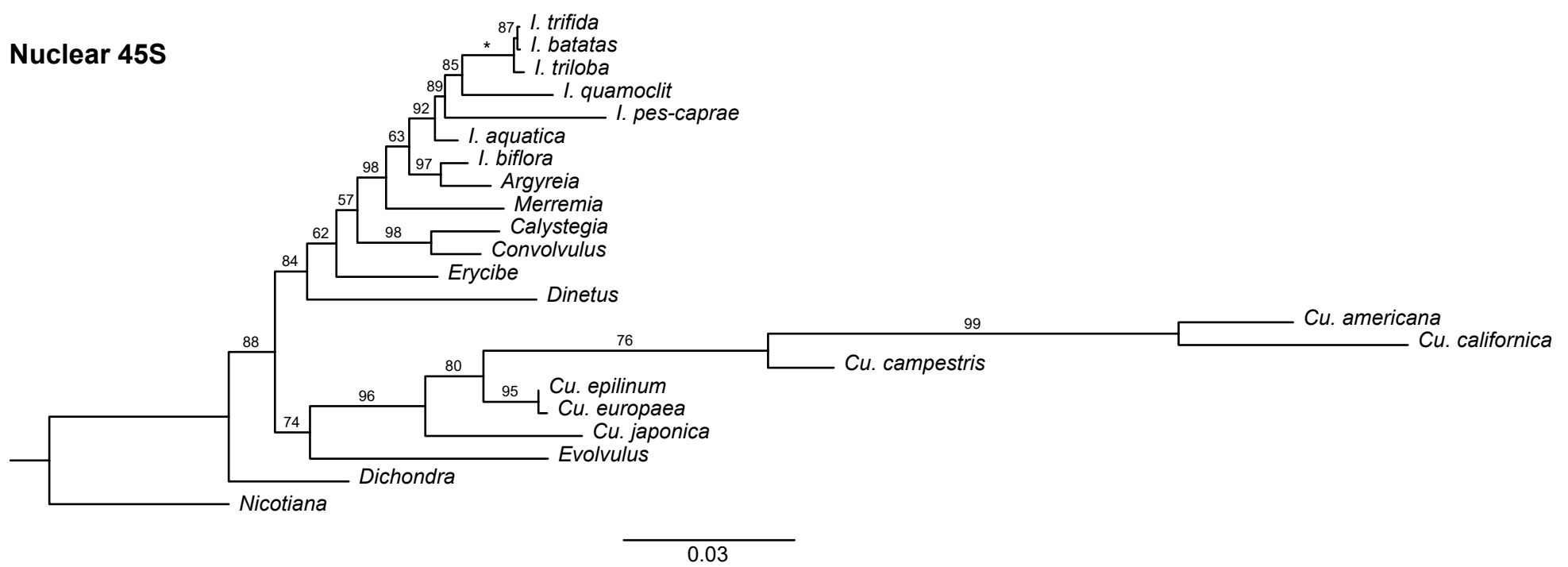

Supplement: Supplementary file 5 — Additional file 5. Plastid CDS, Mitochondrial CDS, and Nuclear 45S. [file 12915_2022_1250_MOESM5_ESM.pdf]

(a)

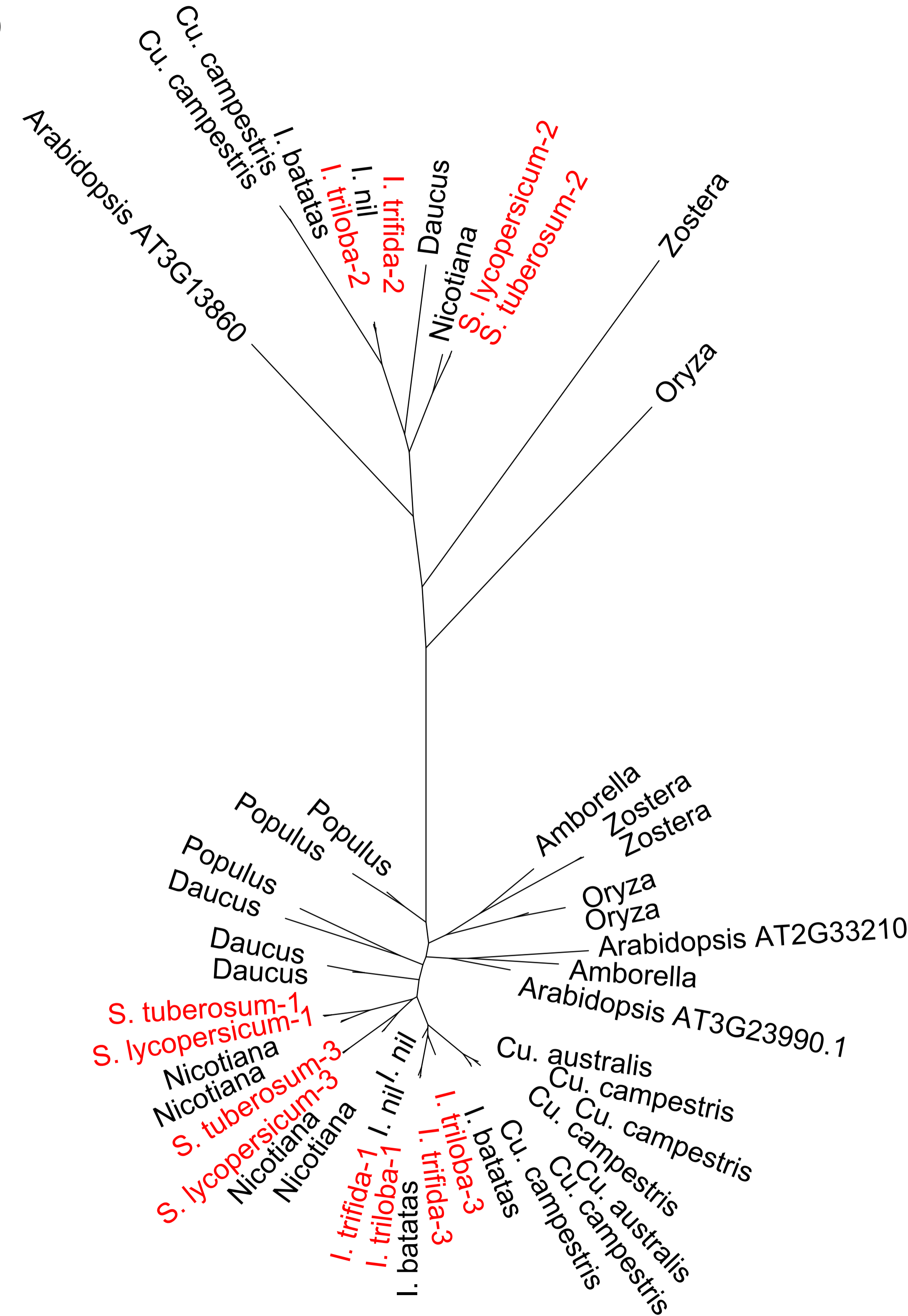

(b)

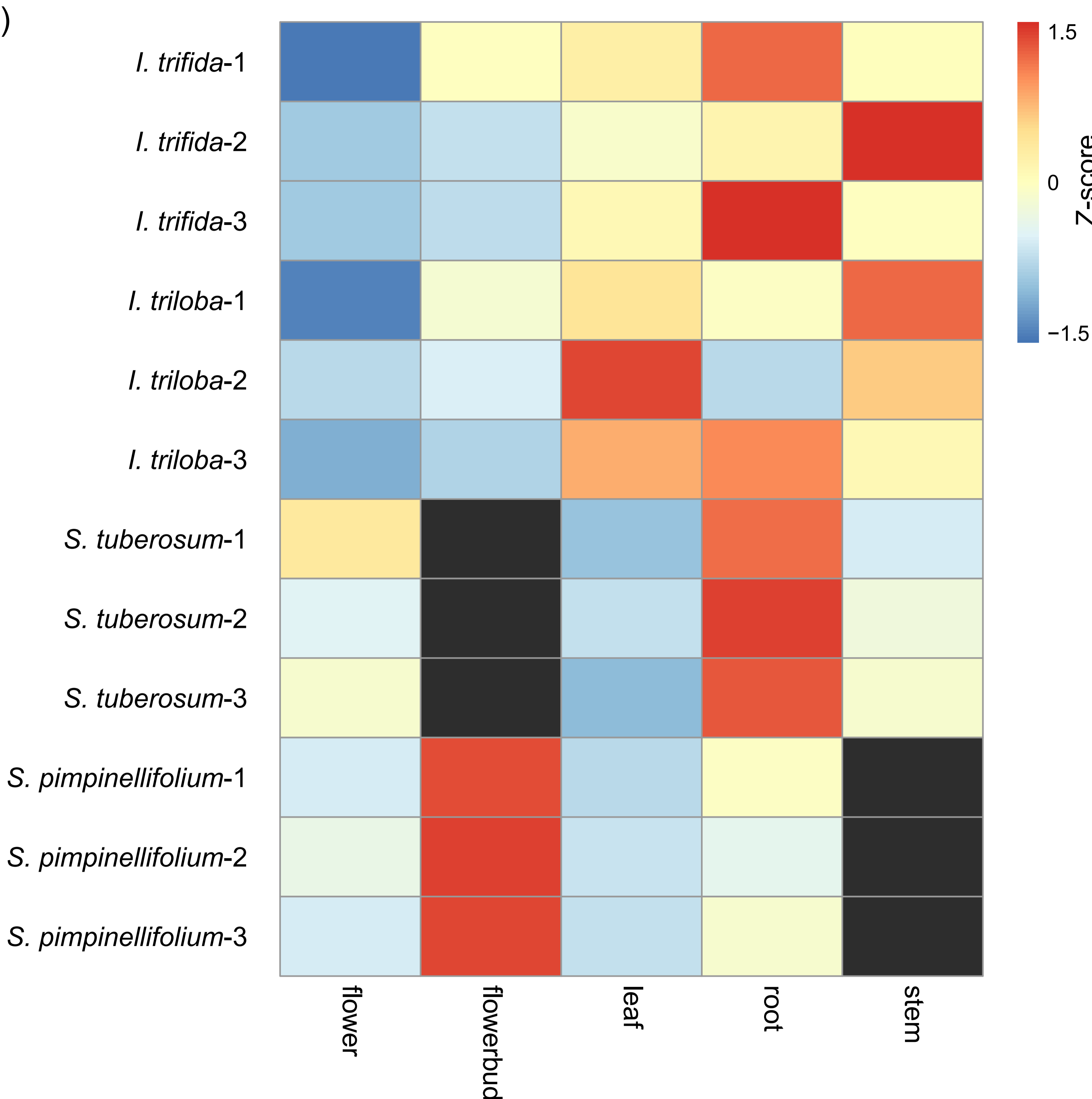

Supplement: Supplementary file 6 — Additional file 6 Similar situation observed for HSP60. [file 12915_2022_1250_MOESM6_ESM.pdf]
